# Conservation opportunities and challenges in Brazil’s roadless and railroad-less areas

**DOI:** 10.1126/sciadv.abi5548

**Published:** 2022-03-04

**Authors:** Trevor R. Tisler, Fernanda Z. Teixeira, Rodrigo A.A. Nóbrega

**Affiliations:** 1 Programa de Pós-Graduação em Análise e Modelagem de Sistemas Ambientais, Universidade Federal de Minas Gerais, Belo Horizonte, Minas Gerais, Brazil.; 2 Transportation Research and Environmental Modeling (TREM) Group, Universidade Federal de Minas Gerais, Belo Horizonte, Minas Gerais, Brazil.; 3 Programa de Pós-Graduação em Ecologia, Universidade Federal do Rio Grande do Sul, Porto Alegre, Rio Grande do Sul, Brazil.; 4 Núcleo de Ecologia de Rodovias e Ferrovias, Universidade Federal do Rio Grande do Sul, Porto Alegre, Rio Grande do Sul, Brazil.

## Abstract

Policy and legislation rarely acknowledge the importance of keeping intact ecosystems road- and railroad-free. By modeling Brazil’s remaining roadless and railroad-less (RLRL) areas, we found that, although they hold the vast majority of the country’s remaining native vegetation (81.5%), because of their limited protection status, only 38% of Brazil’s remaining native vegetation is both protected and in RLRL areas. Current federal policy aims to develop transportation infrastructure designed with antiquated planning methods that threaten remaining intact ecosystems, while concurrently weakening the country’s hallmark environmental protections and commitments. Where Brazil builds its new roads and railroads matters for conservation planning. The occurrence of native vegetation and anthropic land use is associated, at varying degrees, to transportation infrastructure throughout most of Brazil. We highlight that by pursuing conservation opportunities in RLRL areas, Brazil could instead make impactful steps for conservation, restoration planning, and tangible progress toward achieving national and international environmental and conservation commitments.

## Teaser

Modeling Brazil’s roadless and railroad-free areas can guide policy to protect its environmental commitments.

## INTRODUCTION

Terrestrial transportation infrastructure, such as roads and railroads, provoke diverse environmental impacts on ecosystems, including the flora and fauna that compose them. A broad review of worldwide road impacts identified that the vast majority are felt within 1 km of road shoulders, while a noticeable portion are felt as far as 5 km away (*1*). Research in the nascent Railway Ecology field indicates that railroads likely cause many of the same impacts as roads aside from additional identified unique impacts; however, studies are still too few in number to draw firm conclusions on possible differences of impact intensities and spatial reaches to those of roads (*2*–*4*). Nevertheless, the construction of new roads and railroads is considered a driver of land use change, consequently linked to increased deforestation and native vegetation suppression (NVS) (*5*, *6*). What is more, there is consensus that the most environmentally damaging roads and railroads are those that penetrate previously intact and remote ecosystems (*7*).

Worldwide, road and railroad networks are expected to expand by 36 and 45% of their 2010 extents by 2050 (*8*, *9*). A substantial portion of this development (~90%) will occur in tropical countries and will intersect many of the world’s hitherto relatively undisturbed, infrastructure-free, high ecological integrity, and highly biodiverse ecosystems (*10*–*12*). In countries with weak environmental law enforcement, transportation network expansion into these previously inaccessible ecosystems increases their vulnerability to illegal exploitation by hunters, miners, loggers, and colonists (*6*, *7*). A positive feedback cycle of “contagious development,” leading to more roads, more settlers, increased resource extraction, and devastating deforestation and NVS, is commonplace (*1*). Brazil is no exception to this reality and is at the heart of this development pressure.

In the Amazon, a biome of global importance (*13*), infrastructure-induced environmental impacts are well documented; for example, deforestation and NVS overwhelmingly occur within 5.5 km of roads (*8*) and were likely influenced by past railway expansion (*9*). Moreover, roads are suggested links to historic deforestation and NVS in some of Brazil’s other biomes, such as the Caatinga and Atlantic Forest (*14*, *15*). Numerous inventory quantifications related to deforested landscapes and roads exist in the literature, including for the Brazilian Amazon. However, there is a gap in the literature of high-resolution, biome-wide extent statistical analyses of the relationship between roads and deforestation for Brazil’s other biomes. Moreover, we are unaware of any example from the literature that statistically analyzes the relationship of railroad infrastructure to deforestation in any of Brazil’s six biomes, thus indicating a gap in the literature that has timely relevance.

Concurrently, there are calls to expand and improve Brazil’s transportation network to encourage growth of export-oriented commodities sectors, such as soy cultivation, cattle ranching, and mining. These activities heavily rely on environmentally degrading land use practices such as monoculture or strip-mined land covers, topsoil alteration or removal, and deforestation or NVS, and are evermore present in official federal transportation policy for the Amazon and the rest of Brazil’s biomes (*16*). This policy has historic roots in Brazil’s National Transportation System (*Sistema Nacional de Viação*), which consists of nationwide highway and railroad plans that have remained relatively unchanged since the 1970s and still mandates the construction of more than 17,000 km of highways and 19,000 km of railroads that would partially stretch into remote regions of the country (*17*). Conversely, most of Brazil’s environmental protection policies and licensing laws, arguably advanced for international standards, have only been formulated since the 1980s (*18*). Moreover, as the world’s most biodiverse country (*19*), Brazil has also ratified many Multilateral Environmental Agreements (MEAs) since the 1990s, such as the Convention on Biological Diversity (CBD) (*20*), the United Nations Framework Convention on Climate Change (*21*), and the 2015 Paris Agreement (*22*), as well as supported the United Nations 2030 Agenda for Sustainable Development (*23*) and has made deforestation reduction and forest landscape restoration pledges to the Bonn Challenge (*24*) and in its original Nationally Determined Contribution (NDC) to the 2015 Paris Agreement (*25*).

Elements of Brazil’s planned transportation future represent a threat to global biodiversity, carbon sequestration, and ecosystem services conservation (*1*, *18*). Although Brazil has a comprehensive environmental legal framework and has officially incorporated its MEAs into domestic environmental policy, recent environmental catastrophes and environmental regulatory dismantling (*26*), as well as backtracking on commitments to the 2015 Paris Agreement, highlight that there is a stark dichotomy between de jure environmental standards and de facto environmental realities in Brazil (*16*, *25*, *27*–*31*). Besides this, the acknowledged importance of infrastructure-free areas and, moreover, the importance of their size in relation to carbon storage and biodiversity conservation value (*32*) are not present in national environmental law and policy, nor are they commonplace in MEAs (*1*, *10*).

On the flip side, Brazil’s transportation decision-makers do not adequately consider, and sometimes even ignore, the country’s environmental and conservation goals during transportation infrastructure planning (*33*, *34*). Furthermore, MEA commitments have not been appropriately incorporated into transportation policies to ensure that tangible impact reductions and avoiding counteracting plans to “official” environmental policy are achieved. Where and how transportation infrastructure is improved or expanded matters for Brazil’s environmental future and for making progress in meeting its national and international commitments to protecting biodiversity, the environment, and restoring degraded landscapes.

Our study examines Brazil’s roadless and railroad-less (RLRL) areas to investigate overlooked and ignored conservation and restoration opportunities as well as to identify their synergies with domestic environmental law, policy, and MEA commitments. We aim to demonstrate to transportation policymakers and planners the importance of incorporating RLRL areas into planning so as to uphold domestic environmental rule of law and adherence to official national policy, as well as to ensure compliance to Brazil’s international commitments. We modeled Brazil’s RLRL areas by adapting the Ibisch *et al.* (*1*) approach to modeling global roadless areas to include rail infrastructure and country-specific datasets mapped at detailed scales to identify areas that exist at least 1 and 5 km from any documented road, railroad, or pathway in Brazil (Materials and Methods, text S1, figs. S4 and S8, and table S5).

Although the drivers of biodiversity degradation are globally reaching, and global cooperation is needed to confront global environmental change (*35*), national, subnational, and local policymakers and conservation stakeholders have to contend with multiscalar and nuanced local-level realities when implementing conservation programs (*36*). In this study, we present spatially explicit information—which is important for guiding conservation efforts related to the majority of national conservation strategies (*37*)—concerning road and railroad-less areas in Brazil. This study delves deeper than previous roadless area assessments because of our context-specific analysis of the status of Brazil’s RLRL areas at both national and biome-specific levels as well as their synergies with (i) legally protected areas (LPAs), which include all of Brazil’s protected areas in the National System of Conservation Units, titled Indigenous Territories and Maroon Community Lands (*38*–*40*), (ii) the Brazilian Ministry of the Environment’s Priority Areas for Biodiversity Conservation (PABCs) (*41*), and (iii) remaining native vegetation cover identified by Project MapBiomas (text S1 and table S6) (*42*). By identifying Brazil’s RLRL areas and integrating them with the aforementioned variables, we statistically analyze the relationship of deforestation and NVS to distance from roads, railroads, and pathways; we spatially show where future transportation development should be avoided or very carefully considered so as not to contradict national environmental law and conservation policy; we identify where priority conservation efforts should likely be focused so as to make meaningful and rapid impact to ensure ecological integrity; and last, we highlight where potential restoration opportunities should be investigated (figs. S9 to S41).

## RESULTS

### Impacts of transportation infrastructure on deforestation and NVS in Brazil’s six biomes

**Fig. 1.**
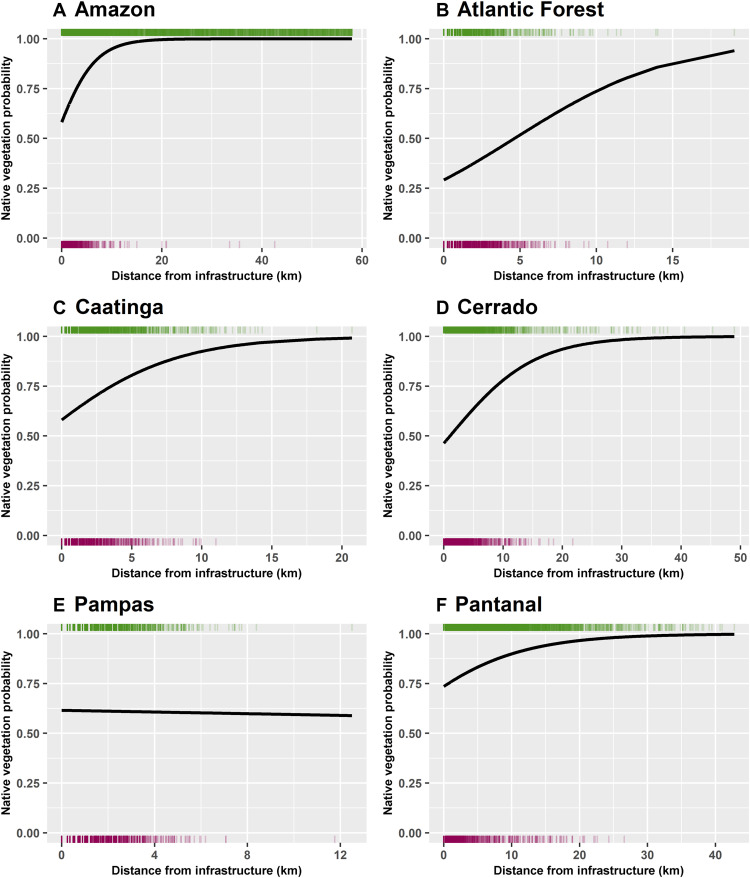
Probability of encountering native vegetation. Native vegetation (green) compared to anthropic land use (purple) as distance increases from transportation infrastructure throughout the entire biome. For all biomes (**A** to **D** and **F**), except the Pampas (**E**), pixels that are farther away from infrastructure (roads, railroads, and pathways) have a higher probability of being covered by native vegetation.

We investigated the relationship between native vegetation versus anthropic dominated land use to distance from transportation infrastructure through running a series of biome-wide and stratified logistic regression analyses. We found that for all biomes, except the Pampas, the probability of encountering native vegetation increases with the distance from transportation infrastructure (Fig. 1 and table S61). In applying three stratified logit models for each infrastructure type (roads, railroads, and pathways), the same general pattern was observed throughout all biomes and all infrastructure, except for roads in the Pampas (see Materials and Methods, figs. S44 to S46, and tables S64 to S66). All resulting model coefficient estimates for the explanatory variable (distance from infrastructure) were statistically significant with *P* values <0.001 and acceptable confidence intervals (tables S61 and S64 to S66). However, the degree to which distance from transportation infrastructure, as the sole explanatory variable, contributed to explaining the total variability within each of these four models for each biome ranged from ~30% explained deviance in the Amazon to less than 1% in the Pampas (tables S61 and S64 to S66).

Consideration of protection status (LPAs) proved to be important for highlighting a bigger picture of where transportation infrastructure affects native vegetation cover and facilitates anthropic land use. We implemented two more stratified logistic regression models, one analyzing the relationship between native vegetation and distance from transportation infrastructure in unprotected portions of each biome, and a second model analyzing this relationship in the protected portions (inside LPAs) of each biome (see Materials and Methods, figs. S42 and S43, and tables S62 and S63). For all biomes, there was noticeable increase in the initial probability of encountering native vegetation in the protected areas–only model when compared to the biome-wide model’s initial probability. These increases of the initial probability for encountering native vegetation within LPAs ranged from a 34.1% increase in the Amazon to a 19.2% increase in the Atlantic Forest (compare Fig. 1 to fig. S43 and table S67). Conversely, the results from the unprotected areas–only model of all biomes demonstrate that there was a slight decrease in the initial probability of encountering native vegetation from this stratified model compared to all biome-wide model’s initial probabilities, which range in a decrease of 3.17% for the Amazon to a decrease of 0.42% in the Pampas areas (compare Fig. 1 to fig. S42 and table S67). The degree to which distance from transportation infrastructure contributed to explaining the total variability, as the sole explanatory variable, was higher for each biome’s stratified protected model than for their stratified unprotected models (tables S62 and S63).

Our model indicates that the most distant locations from a road, railroad, or pathway in each biome are as follows: for the Amazon, 168.05 km; for the Atlantic Forest, 19.04 km; for the Caatinga, 20.72 km; for the Cerrado, 48.97 km; for the Pampas, 12.56 km; and for the Pantanal, 42.78 km (data S3).

### Brazil’s RLRL areas and their protection status

Although a large part of Brazil’s continental territory is 1 km or more away from any road and railroad (71.4%, 607 Mha), and all of Brazil’s biomes hold measurable extensions of these 1-km RLRL areas (table S8 and data S1), a much smaller part of the country is 5 km or more away from the closest road and railroad infrastructure (39.2%, 333 Mha) (table S8 and data S1). When we disentangle the impacts of roads, railways, and pathways on identified RLRL areas, roads are predominantly the closest infrastructure class to most portions of RLRL areas. Pathways are the second most dominant infrastructure class nearest to portions of RLRL areas, and railroads are the least dominant infrastructure class (figs. S47 to S64 and tables S68 to S70).

**Fig. 2.**
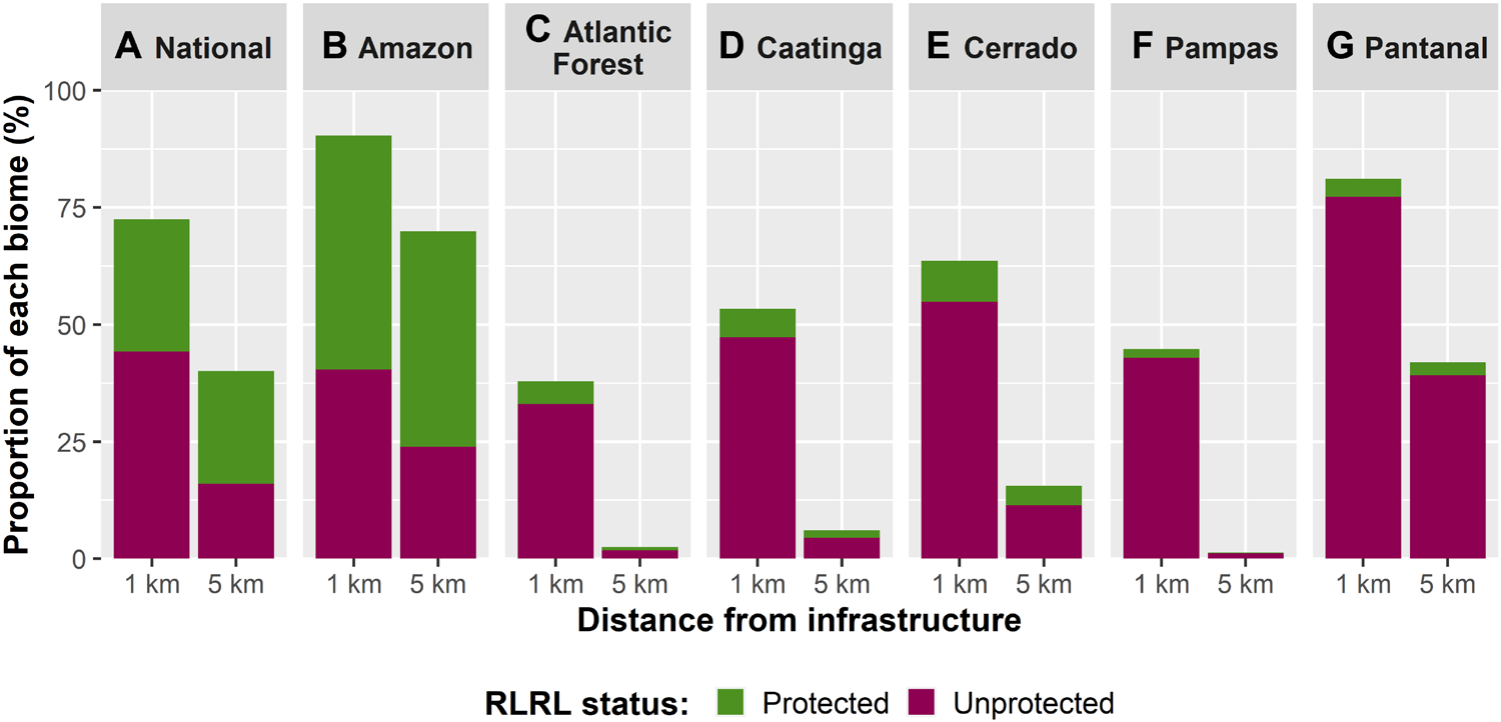
Brazil’s RLRL area coverage and protection status. At the national scale (**A**), Brazil’s 1- and 5-km RLRL areas cover large parts of national territory and have notable levels of protection. However, the sheer size of (**B**) the Amazon, which covers 49.6% of national territory, and the overall coverage and protection status of that biome’s RLRL areas skew the national level statistics. While the overall territorial coverage of RLRL areas in Brazil’s other five biomes vary (**C** to **G**), in each of these biomes, their 1- and 5-km RLRL areas are all overwhelmingly unprotected.

**Fig. 3.**
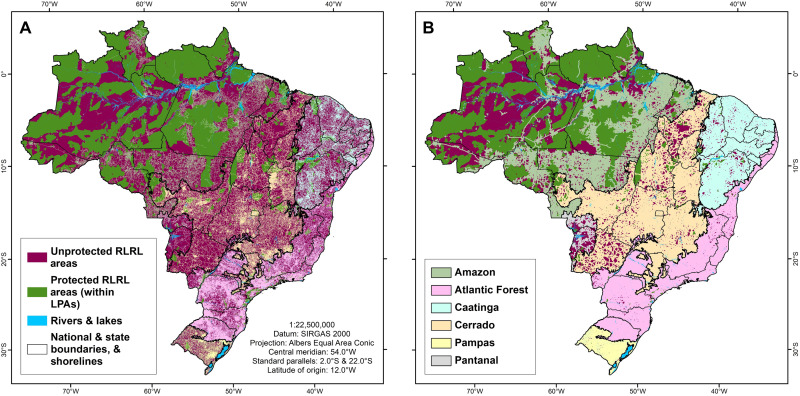
Spatial distribution of Brazil’s protected and unprotected RLRL areas. (**A**) Brazil’s 1-km RLRL areas are found in high area coverage in all six biomes. However, only protected 1-km RLRL areas account for a high proportion of overall RLRL areas and cover large portions of the Amazon biome, whereas unprotected 1-km RLRL areas dominate the RLRL area status in Brazil’s five other biomes and cover those biomes’ areas at varied proportions. (**B**) Brazil’s 5-km RLRL areas are not evenly distributed among Brazil’s biomes. While found in abundance and highly protected in the Amazon and in abundance but unprotected in the Pantanal, Brazil’s four other biomes hold much lower proportional 5-km RLRL area coverage. Consequently, the lower the proportional 5-km RLRL coverage reflects, the earlier a biome witnessed its initial colonization and settlement since Brazil’s colonial era.

Overall, total RLRL area coverage is not evenly spread across Brazil’s six biomes, especially for 5-km RLRL areas (Figs. 2 and 3). In consideration of the protection status of RLRL areas, the Amazon is the only biome holding more protected RLRL areas, either 1 or 5 km and overlapped by LPAs, than unprotected RLRL areas. RLRL areas in Brazil’s five other biomes have overwhelmingly been left unprotected (Figs. 2 and 3 and tables S9 to S10), which are predominantly on private lands, government-owned lands that are not conservation-focused, or lands of unknown tenure status.

### Native vegetation in Brazil’s RLRL areas

**Fig. 4.**
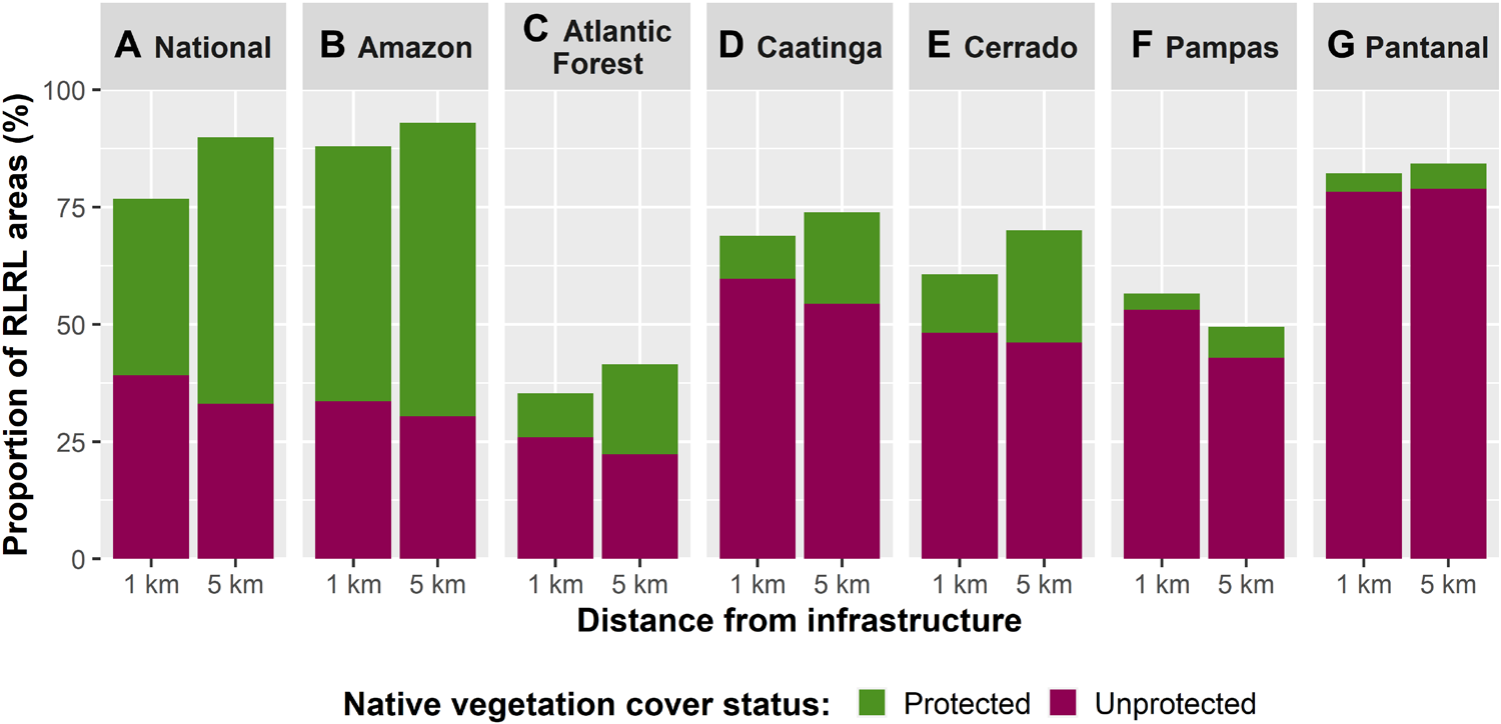
RLRL areas covered with remaining native vegetation and its protection status. At the (**A**) national level, native vegetation covers most of Brazil’s 1-km (76.4%) and 5-km (90.1%) RLRL areas, with 35.6% of 1-km RLRL areas and 56.8% of 5-km RLRL areas covered by protected native vegetation. However, the level of (**B**) the Amazon’s native vegetation cover of its 1- and 5-km RLRL areas as well as their high protection levels skew the (A) national statistics. While the native vegetation coverage of the RLRL areas varies for each of Brazil’s other five biomes (**C** to **G**), the native vegetation that exists in their RLRL areas is left overwhelmingly unprotected (tables S33 to S44).

**Fig. 5.**
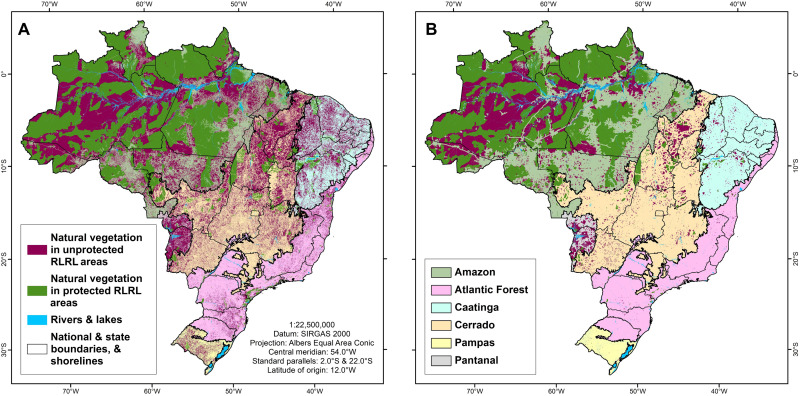
RLRL areas remaining native vegetation cover and its protection status. For 1-km RLRL areas (**A**), one can see that native vegetation covers most of these areas in the Amazon and Pantanal. However, for the Caatinga and Cerrado biomes, native vegetation cover within 1-km RLRL areas is noticeably “thinner” in comparison to those biomes’ overall 1-km RLRL area coverages [visually compare (A) to Fig. 3A]. As for the Atlantic Forest and Pampas, native vegetation covers low amounts of the biomes’ 1-km RLRL areas [visually compare (A) to Fig. 3A]. For 5-km RLRL areas (**B**), the same trend is noticeable for the Amazon and Pantanal biomes, native vegetation covers the vast majority of those biome’s 5-km RLRL areas. As for the Caatinga and Cerrado biomes, there are areas of noticeably “thin” native vegetation coverage of each biome’s 5-km RLRL areas [visually compare (B) to Fig. 3B]. For the Atlantic Forest and Pampas biomes, there is exceptionally low native vegetation cover for each biome’s already small and limited 5-km RLRL areas [visually compare (B) to Fig. 3B].

When native vegetation cover is added to the picture, Brazil’s 1- and 5-km RLRL areas contain 81.5% (43 Mha) and 52.8% (300 Mha) of the country’s total remaining native vegetation (568 Mha as of 2017). Conversely, with a focus on how much of the RLRL areas are vegetated or not, at the national level, 76.4% of 1-km and 90.1% of 5-km RLRL areas are covered by the remaining native vegetation (Fig. 4 and tables S33 to S44). However, both of these national-level results do not represent an even spread across Brazil’s biomes, and individual biome-level results seem to reflect each biome’s different state of overall degradation from deforestation and NVS (Figs. 4 and 5).

Regarding the protection status of Brazil’s remaining native vegetation that also lies within RLRL areas, at the national level, 38% (216 Mha) and 33.2% (189 Mha) of the country’s remaining native vegetation are both protected by an LPA and found within a 1- or 5-km RLRL area (Fig. 5 and tables S54 and S55). Conversely, 43.5% (247 Mha) and 19.5% (111 Mha) of Brazil’s remaining native vegetation are found in unprotected portions of 1- or 5-km RLRL areas (Fig. 5 and tables S54 and S55). However, these national averages are heavily distorted by the Amazon, as it alone holds 86% (197 Mha) of the country’s remaining protected vegetation (228 Mha) as well as 44.9% (152 Mha) of the country’s total unprotected vegetation (340 Mha) (Fig. 5 and table S58).

### RLRL area synergies with environmental policy

The Brazilian Ministry of the Environment has identified PABCs in all of Brazil’s six biomes to inform and guide conservation policy and programs (*41*, *43*). PABCs are one of Brazil’s key strategy tools for working toward achieving Aichi Biodiversity Targets 2, 7, 11, 14, and 15 and as part of the country’s action plan for achieving the United Nations Sustainable Development Goals, specifically goal 15 (text S1) (*23*, *43*). Moreover, these PABCs could serve as opportune areas for restoration activities as a means to fulfill ecosystem restoration commitments and pledges. When simultaneously combining Brazil’s identified but still unprotected PABCs (not protected by LPAs), remaining native vegetation cover, and their resulting overlays within RLRL areas, important conservation and restoration priority estimates become apparent.

**Fig. 6.**
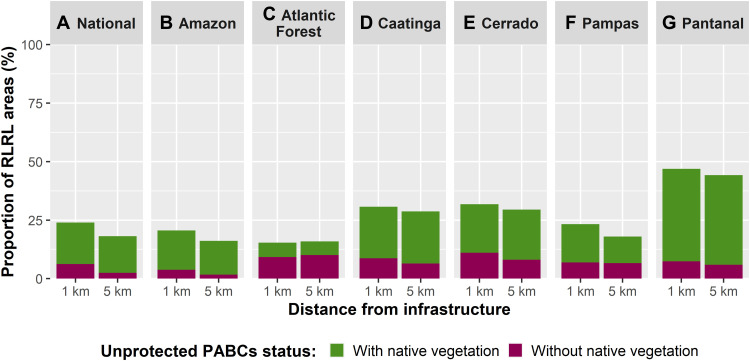
Opportunities for conservation and restoration. Unprotected PABCs that overlap with RLRL areas are opportune starting points to make urgently needed progress toward achieving Brazil’s conservation goals and commitments. Specifically, those overlapping PABCs and RLRL areas at the national level (**A**) and individual biome levels (**B** to **G**) that currently hold native vegetation (green) would be immediate gains, whereas those areas without native vegetation cover (purple) may be prime candidates for restoration in the case of anthropogenic land cover or may be synergistic conservation opportunities, in the case of abiotic land cover, when paired with native vegetation conservation.

Nationally, 17.7% (107 Mha) and 15.7% (52 Mha) of 1- and 5-km RLRL areas are jointly covered by native vegetation and in unprotected PABCs, representing key conservation priorities (Fig. 6, figs. S11 and S12, and tables S47 and S50). Moreover, a small but important portion of abiotic dominated land cover (e.g., rivers and lakes, rocky outcrops, and sand dunes) is found in unprotected PABC overlaps with RLRL areas, accounting for 1.1% (6.5 Mha) of 1-km and 1.2% (4 Mha) of 5-km RLRL areas nationwide (tables S48 and S51). These abiotic natural landscape features are also important conservation priorities alongside areas with native vegetation, as they commonly contain species with habitat requirements that are restricted to these naturally unvegetated areas. Moreover, abiotic landscapes, and their species, commonly provide supporting functions to the surrounding and unrestricted biodiversity. Conversely, in focusing on potential restoration opportunities, there are some areas that may serve as initial starting points. At the national level, 5.5% (33 Mha) of 1-km RLRL and 1.6% (5.4 Mha) of 5-km RLRL areas fall within unprotected PABCs that have predominantly anthropogenic land covers and little to no remaining native vegetation (Fig. 6, figs. S11 to S12, and tables S49 and S52). At the biome-specific level, areas of anthropic land use found in overlapping unprotected PABCs and RLRL area regions account for a notable proportion of both 1- and 5-km RLRL areas that remain in the Atlantic Forest and Cerrado, the former being the country’s most degraded biome and the latter being highly threatened. In addition, anthropic land use in overlapping portions of RLRL areas and unprotected PABCs account to discernible portions of the remaining 1- and 5-km RLRL areas in the Caatinga and Pampas, which are biomes that have also suffered high to severe levels of overall degradation. If these areas were to undergo restoration activities, measurable positive environmental impacts could be achieved. Moreover, these areas would need to remain infrastructure free to aid in achieving success under such programs.

## DISCUSSION

### Brazil’s environmental (dis)governance

Brazil’s commitments to international MEAs include reducing deforestation rates in the Amazon to net 0% by 2030 as promised in its original NDC (*25*), as well as conserving 30% of the Amazon and 17% of each of its five other biomes and restoring severely degraded ecosystems under the CBD (*43*). Moreover, Brazil pledged to actively reforest 12 Mha of deforested and degraded land under commitments made both in the country’s original NDC and to the Bonn challenge (*25*, *44*). Currently, only de jure conservation in the Amazon meets CBD goals, whereas Brazil’s five other biomes show de jure conservation deficits (tables S59 and S60).

Yet, as recent events demonstrate the failure, unwillingness, and even antipathy of the current government for protecting the country’s environmental patrimony against the direct and indirect degradative impacts of unchecked environmental crimes and illegal activities (*45*–*48*), the de facto reality is that none of Brazil’s biomes are securely conserved and protected (*26*). However, vegetated RLRL areas in these biomes are ample enough that if they were turned into de jure LPAs as well as actually (de facto) protected—as opposed to the current trend of enfeebling LPA management and the law enforcement agencies charged with protecting them (*26*)—they would be sufficient enough to achieve or make significant progress toward achieving CBD goals in all biomes as well as to make progress toward achieving Brazil’s other MEA commitments without the need to backtrack.

Brazil’s federal government claims that the country is ahead of the global conservation curve as it implements its nationwide Rural Environmental Registry (CAR, in Portuguese), mandated by the country’s extensively studied 2012 Forest Code (FC) (*49*). Brazil’s CAR is a land registry system specifically mandated by the FC for the purpose of maintaining and enforcing native vegetation cover minimums on rural private properties. With the exception of a few states, progress in implementing the FC has been slow (*50*) and is only mandated to be completed by 2032 (*51*); it has repeatedly come under political attack (*52*) and depends on property holders (~50% of whom in the Amazon and Cerrado are already noncompliant) to design and implement their own conservation and restoration plans (*28*). These plans will be regulated and enforced by state-level environmental agencies that were left with no guidance from the federal government on how to appropriately regulate the FC (*53*). The efficacy and realization of a fully implemented FC that achieves high value conservation by 2032 is bleak. Our stratified logistic regression models show that the areas of Brazil where CAR is the principal conservation tool with legal standing—unprotected areas outside of LPAs—have higher probabilities to suffer from transportation infrastructure related deforestation and NVS.

Our results highlight that more needs to be conserved in Brazil and recurring attempts of protected areas downgrading, downsizing, and degazettement (PADDD) are moving policy in the wrong direction (see https://padddtracker.org/). Our results indicate that protection status is important for reducing the levels of deforestation and NVS that occur in relation to infrastructure location; however, this does not mean that planning infrastructure in LPAs is a way to reduce their environmental impact. Instead, new protected areas can be created and actually managed as a strategy to contain deforestation. Considering that the construction of transport infrastructure is used as a justification to downgrade, downsize, and degazette protected areas (*54*, *55*), future planning efforts should spatially identify where PADDD attempts would likely occur for planned infrastructure development to continue and instead propose alternative routing options that do not pose PADDD threats (*56*). The protected versus unprotected RLRL areas identified here could serve as starting points to identify which protected areas should remain road-free, railroad-free, and defended against PADDD attempts as well as to identify where new protected areas could be created. Other types of linear infrastructure, such as pipelines and transmission lines—while holding some differing environmental impacts to those caused by roads and railroads—may also pose PADDD threats and should also be planned to avoid protected areas and their RLRL areas.

### The ways forward for Brazil’s biomes

Our biome-specific analyses of RLRL areas and their synergies with key conservation factors allowed for the emergence of three distinct trends that differentiate the context-specific realities for each of Brazil’s six biomes. These differences are important for both conservation planning and transportation planning to consider when formulating policies that could have impacts within specific biomes as well as produce impacts that span across multiple biomes. This strategy is well suited to complement Brazil’s biome-oriented environmental legislation while also avoiding contradiction with nationwide blanket environmental laws.

The first trend includes the Amazon and Pantanal biomes. These biomes hold patches of RLRL areas that are very large in size (patch-extent) and that contain abundant levels of native vegetation. The combined coverage area of these biomes’ large RLRL area patches amounts to a large portion of each biome’s overall area (figs. S19 and S39). Moreover, these biomes’ remaining 5-km RLRL areas account for 68.3% (288 Mha) of the Amazon’s area and 41.9% (6.3 Mha) of the Pantanal’s area, and the unprotected portions of these areas account for 23.9% (100 Mha) of the Amazon’s and 39.2% (5.9 Mha) of the Pantanal’s total areas, respectively (tables S8 to S10). Furthermore, when overlaying PABCs with these unprotected vegetated 5-km RLRL areas, the delineated areas still hold 11.9% (41.7 Mha) of the Amazon’s remaining native vegetation (at 9.9% of the Amazon’s area) and 20.2% (2.4 Mha) of the Pantanal’s remaining native vegetation (at 15.8% of the Pantanal’s area) (tables S53 and S57). However, illegal deforestation and fires in 2019 and 2020 in both of these biomes have likely reduced these numbers (*45*, *46*).

These areas, especially those falling in the delineated overlaps of all three variables (RLRL areas, native vegetation cover, and PABCs) are opportune for immediate and appropriate conservation protection and, moreover, should be avoided at all costs by transportation planners (figs. S21 and S41). It is important to stress that the overall vegetation abundance and ample conservation choices should not be misconstrued as also representing an opportunity to allow for more deforestation and NVS or a greenlight for transportation development without due diligence studies in other unprotected and nonconservation priority areas. Both of these biomes are of extreme importance because of the significant ecosystem services they provide at national and international scales, and both biomes’ ecosystem functionalities are highly sensitive to deforestation and NVS, which, if continued, could lead to either ecosystem crossing irreversible thresholds (*12*, *13*, *57*, *58*). While Brazil has achieved its commitment to legally preserve 30% of its Amazonian biome, published modeling results suggest that greater than or equal to 60% of the Amazon’s original vegetation extent should be protected to avoid triggering a massive forest dieback event (*12*, *57*). Recent widespread illegal deforestation and fires, increasing cases of land grabbing, and ever more common invasions of protected areas in both biomes are tied to the concurrent dismantling of Brazil’s environmental law enforcement. These actions have coalesced to such a grim level that any glimmer of hope for achieving necessary conservation requirements to avoid crossing ecological thresholds in either biome has never been more unlikely. Therefore, for all intents and purposes, Brazil’s achievement of protecting 30% of its Amazonian territory is anticlimactic and the 17% conservation goal for the Pantanal is not enough.

The second trend highlights urgent conservation priorities that should be considered seriously for the Atlantic Forest and the Pampas, the two biomes with the lowest amounts of 1- and 5-km RLRL areas proportional to their total biome areas (tables S8 to S10). These biomes have a relatively low number of 5-km RLRL area patches, they are relatively smaller in size compared to 5-km RLRL patches found in Brazil’s other four biomes, and the combined area of these 5-km RLRL patches proportionally covers little of either biome’s total areas (figs. S23 and S35 and tables S8 to S10). Furthermore, the majority of the remaining native vegetation in these biomes’ 5-km RLRL area patches is unprotected, 53.7% (621 Kha of 1.1 Mha) is unprotected in the Atlantic Forest and accounts for just 1.8% of the biome’s overall remaining native vegetation, and 86.6% (90 Kha of 104 Kha) is unprotected in the Pampas, which accounts for just 0.96% of the biome’s overall remaining native vegetation (figs. S23 and S35 and table S55). Of this unprotected native vegetation found in both biomes’ 5-km RLRL areas (the most “remote” areas), a “sizable” percentage of this vegetation is also located in unprotected PABCs, 25.9% (157.6 Kha of 607 Kha) in the Atlantic Forest and 26.6% (24 Kha of 90 Kha) in the Pampas, which only accounts for 0.46 and 0.25% of each biome’s overall remaining native vegetation (figs. S25 and S37 and table S57). Given the overall state of natural landscape degradation in both these biomes, these spatially identified overlaps represent paramount priority starting points for achieving conservation targets as they are the very few areas of both biomes that have ample distance from existing infrastructure and hold remaining native vegetation.

Of Brazil’s six biomes, the Atlantic Forest has suffered the most proportional native vegetation loss and anthropic landscape expansion since the colonial era, with the Pampas following in second place (*58*). Moreover, these biomes have exceptionally low remaining native vegetation coverage even in their 1-km RLRL areas. Conservation in these biomes will likely have to incorporate both 1- and 5-km RLRL areas into a complex multipronged approach: saving what little is left, conserving any and all remaining native vegetation within and outside of RLRL areas, and trying to restore already degraded areas, especially within degraded RLRL areas, to create biodiversity corridors and build biome-level resilience in the face of climate change. As for transportation planning, any 1- and 5-km RLRL area with native vegetation and within a PABC should be avoided, and rerouting some meso- and macroregional transportation plans via different routes may also be necessary to restore ecological corridors and expand the overall integrity of these biomes’ RLRL areas. This is especially important as the roads within the Atlantic Forest biome concentrate most of Brazil’s vehicle traffic and have a high number of roadkills reported (*59*), which has the potential of decreasing the persistence of wildlife populations in the remaining habitats (*60*).

The third trend highlights the Caatinga and Cerrado, the two biomes in the middle range compared to Brazil’s other four biomes because of their proportional biome-level coverage of remaining 1- and 5-km RLRL areas. The Caatinga and Cerrado biomes have few RLRL area patch options for conservation, but the overall proportional area gains from conserving these few identified patches would still be noteworthy. When analyzing the status of the biomes’ 5-km RLRL areas, although their formed patches and delineated overlays with unprotected PABCs and native vegetation are few in number and in total do not cover either biome at a large proportional extent (as opposed to the Amazon and Pantanal), the 5-km RLRL area patches that do exist are still much larger in area than the remaining 5-km RLRL area patches found in the Atlantic Forest and the Pampas (figs. S27 and S31 and data S1).

In recent decades, both the Caatinga and Cerrado have suffered from increasing deforestation and NVS (*61*), more markedly so in the Cerrado than in the Caatinga due to policy and economic forces encouraging the rapid expansion of soy monoculture landscape (*62*). Moreover, both biomes have received relatively low conservation priority and action, the Caatinga receiving the lowest amount of attention than Brazil’s other biomes (*61*). Conservation in these two biomes should likely focus on conserving the currently available advantageous opportunities, especially those in PABCs that fall in heavily degraded ecoregions, before they succumb to further degradation or continued agricultural expansion (*62*). Secondly, conserving the areas that could be used as biodiversity corridors (remaining 1-km RLRL areas, their PABC overlaps, and remaining native vegetation) is still a possibility; however, restoring degraded areas may also be necessary in some instances. Transportation planning in these biomes will need to follow a dynamic approach. First, planned new infrastructure routes and improvements should be avoided when near to large unprotected RLRL patches with native vegetation and PABC designation. Moreover, meso- and macroregional improvements of already existing infrastructure should undergo careful consideration to avoid the possible future economic pressures (e.g., incentivizing an expanding agricultural frontier) they could place on RLRL patches that may be opportune restoration locations because of their PABC classification.

### Conservation and restoration actions are urgently needed

In relation to Brazil’s commitments to many MEAs, there has been a general track record of failure in fully meeting promised goals, and now, there are efforts to backtrack on original commitments so as to avoid having to produce results (*30*). This is most apparent with Brazil’s conservation deficits in five of its biomes for the CBD, specifically for meeting Aichi Targets 11 and 15, because all biomes, except the Amazon, have deficits ranging from roughly 6 to 14% for achieving minimum Aichi Target 11 area conservation goals (tables S37 and S38). RLRL areas with native vegetation that are concurrently part of unprotected PABCs serve as advantageous opportunities to work toward meeting Aichi Target 11. The RLRL areas that are in unprotected PABCs but that do not have native vegetation cover may serve as starting points for restoring Brazil’s most degraded biomes to work toward meeting Aichi Target 15 goals. These areas simultaneously serve as opportunities to make progress toward achieving Brazil’s commitments to the Sustainable Development Goals and its original NDC and Bonn Challenge forest-landscape restoration pledges.

Avoiding further habitat loss and fragmentation is crucial for the success of Brazil’s conservation policy and MEA commitments that have been pledged to Brazilian citizens and the international community. Policies from other countries can be reference models for Brazil to build upon as a way to recognize the importance of RLRL areas, such as the United States Forest Service’s (USFS’s) Roadless Area Conservation Rule (*63*, *64*), although this rule does not allocate protected area status to its identified roadless areas and many environmentally impacting activities are still permitted in them (*63*). Therefore, the highlighted RLRL areas should be incorporated into conservation, transportation, and all other relevant policymaking realms affecting land use. While biome-level differences indicate that tailored conservation and transportation planning approaches are needed, the overall impact of incorporating Brazil’s RLRL areas as guiding posts for transportation planners and conservation planners offers a synergistic and productive framework to reduce policy and legal conflicts.

## MATERIALS AND METHODS

### Study area

Our study area includes the total continental territorial extent of Brazil, excluding maritime territory, outer lying islands in the Atlantic Ocean (such as Fernando de Noronha), and two major water bodies (Lagoa dos Patos and Lagoa Mirim). Furthermore, Brazil encompasses six classified terrestrial biomes that include the Amazon, Atlantic Forest, Caatinga, Cerrado, Pampas, and Pantanal (fig. S1). Although five of six of Brazil’s biomes also extend into neighboring countries, our analysis only focuses on the Brazilian portions of these biomes. To identify the divisions of these biomes in our modeling and analyses, we combined official national boundary spatial data as well as biome limits developed by Rosa and Project MapBiomas (table S1) (*65*, *66*). Geospatial analyses were conducted using ArcGIS v10.4.1 (*67*) and R language (*68*), and all input data, whether in vector or raster format, were projected to the Brazilian Institute of Geography and Statistics’ (IBGE’s) standard Albers Conic Equal Area projection for Brazil (fig. S2 and table S2) (*69*).

### Spatial modeling approach

Inspired by the global scale roadless areas analysis conducted by Ibisch *et al.* (*1*), we modeled Brazil’s RLRL areas within a comprehensive framework by adding an additional infrastructure category (railroads) and by also using country-specific data sources whose finer scales are better suited for country-level modeling and analysis. We determined that Brazil’s RLRL areas should be modeled at 1 and 5 km in distance from any documented road, railroad, or pathway feature. Following the methodology of Ibisch *et al.* (*1*), we included the documented pathways from the OpenStreetMap (OSM) (*70*) in the model (table S4 for more details). We argue that pathways, especially in remote landscapes, should be treated like roads. All road categories have impacts (*1*), whether large or small or whether paved or dirt. Pathways could possibly be used by all-terrain vehicles, which could potentially affect surrounding flora and fauna as well as be used for colonization and human settlement, which in extreme cases could be facilitated by foot travel. While we decided to treat roads, railroads, and pathways in a similar fashion for our analysis, we encourage the scientific community to investigate the impacts of railroads and pathways further to determine whether different impact patterns exist.

Rivers also serve as important transportation corridors for human economic activities and migration. However, we decided not to include navigable rivers in our analysis for the following reasons. First, although past studies have indicated that navigable rivers have some degree of influence on deforestation patterns in the Brazilian Amazon (*8*), their direct contribution to overall deforestation is much lower than terrestrial transportation infrastructure. Second, given that this study uses Ibisch *et al.* (*1*) as the methodological starting point, that study also did not include rivers for the global level analysis. Last, rivers are natural features that, for all intents and purposes, are spatially located outside the control of human influence, except in instances of costly and large-scale engineering and damning projects. Therefore, we decided not to include rivers in the scope of modeling RLRL areas. However, we still do caution that proposed large-scale development projects to increase transportation access or capacity on rivers could cause significant changes to the land use and land cover in any biome. Other types of linear infrastructure, like pipelines and power lines, also influence development in their surroundings (*71*), but they were also not included in our study as their pattern of use influence in land cover and land use might be different from that of transport infrastructure. We, thus, recommend that future studies investigate the effects of other types of linear and transport infrastructure on NVS.

Brazil shares an extensive land border with 10 neighboring countries that totals 16,145 km and has a significant coastline of 7491 km (*72*). Given that Brazil only has sovereign control over its territory and does not control if roads or railroads are constructed on the other side of its borders, our measurement and identification of the RLRL areas stops at Brazil’s borders and coastline. It is likely that many of Brazil’s RLRL areas do continue to extend into neighboring countries; however, their identification was not included in the scope of this study.

### Spatial data sources

IBGE’s official spatial data identifying Brazil’s terrestrial biomes (scaled 1:5,000,000) and IBGE’s official data for identifying Brazil’s territorial extent in the 2017 1:250,000 scaled Continuous Cartographic Database of Brazil (BC250) are not available in the same scale and, as a result, vary significantly in their territorial coverage (*66*, *73*). Therefore, the national and biome-specific area measurements for our study areas are defined by overlaying an adaptation of IBGE’s spatial biome data that was developed by Project MapBiomas (*65*), which provides a lower amount of spatial area coverage inconsistencies, with IBGE’s 2017 BC250 national territorial data as opposed to IBGE’s own official biome spatial data (*66*). In combining the overlapping area of these two data sources, the defined study area when considering the overlaid terrestrial biomes by MapBiomas covers 849,957,609.87 ha, although the national area coverage of IBGE’s 2017 BC250 national territorial extent spatial data is equal to 851,419,849 ha when protected using IBGE’s official projection (table S3) (*66*). This difference in area of our study area to that which is reported by IBGE (*66*) is principally the result of excluding major maritime bodies of water (e.g., large bays or straits between islands and mainland), which are not a focus of our analysis, and updated maritime island coastlines.

According to IBGE (*69*), “The total value of the Brazilian surface was kept at 8,515,767.049 km^2^ (851,576,704.90 ha), as published in the Brazilian Official Gazette of no. 118 of June 22, 2016, according to Resolution no. 02, of June 21, 2016.” However, using data from IBGE’s 2017 BC250 and having to correct for errors in the shapefile geometry and using IBGE’s official projection, we calculated Brazil’s national territory at 8,514,198.492 km^2^ (851,419,849.20 ha), a difference of 1568.557 km^2^ (156,855.70 ha) (table S3). It is important to note that 77% of this appears to be Lagoa dos Patos and Lagoa Mirim in Rio Grande do Sul and 23% appears to be land along Brazil’s shoreline and with small coastal islands. Moreover, with overlaying MapBiomas Biome Definitions and Classifications of Land versus Water, more areas of discrepancies were captured by the differences in scale and along Brazil’s shoreline (fig. S3).

To model our RLRL areas, we incorporated road, railroad, and pathway data (table S4). Focusing on roads, we specifically used both IBGE’s spatial road network data from the BC250 (*66*) and OSM (*70*) (which included the pathway data) to ensure that all known roads (officially documented or crowdsource identified) were included in our model. OSM data include identified pathways, such as bridleways and hiking footways, that are not automobile-dominated roads. As these categories were included by Ibisch *et al.* (*1*), we decided to include all of these types of “roads” in our model database. The accuracy and completeness of OSM data have been documented to vary among different regions in Brazil (*1*). Although OSM appears to have larger geographic coverage than IBGE’s 2017 BC250 national road data, it is possible that some officially documented roads from IBGE could have been left out of the model if we solely depended on OSM. Thus, both data sources were included in the model to err on the side of caution (fig. S4).

As for the railroads included in our spatial database, four data sources for existing railroads were used: (i) OSM’s railroad data (*70*), (ii) MTPA’s Transportation Information Database (for *Banco de Informações de Transportes*) (*74*), (iii) the National Agency for Terrestrial Transport’s (for *Agência Nacional de Transportes Terrestres*) Georeferenced Federal Railroad Network (*Malha Ferroviária Federal Georreferenciada*) (*75*), and (iv) IBGE’s 2017 BC250 (table S5) (*66*). These different sources were used to capture all railroads that could fall under the jurisdiction of federal, state, or municipal governments. Moreover, there were some discrepancies in the operational status of railroads as documented by different government sources. Therefore, by using multiple sources, we were able to guarantee that all built railroads (whether in operation, partially built, abandoned, or unknown operational status) were included in the model (fig. S4).

We identified four principal environmental and biodiversity conservation items that were important to spatially investigate in relation to RLRL areas, which include Brazil’s LPAs as of 2018 (*38*–*40*), PABCs (*41*), Remaining Native Vegetation as of 31 December 2017 (*42*), as well as MEA commitments to the Aichi Biodiversity Conservation Targets and Sustainable Development Goals and included in domestic policy via Brazil’s National Biodiversity Strategy and Action Plan (see text S1 and table S6) (*43*). The spatial data used to identify and analyze these environmental and conservation aspects are outlined in table S6, and the process for identifying these items is described in further detail as well as visually identified in figs. S5 to S7. The framework for using the spatial data to analyze progress toward meeting MEA commitments is analyzed after the spatial data are described (text S1).

### RLRL area modeling

Basic data preprocessing, spatial analysis, and area calculations for arriving at the results presented throughout this study were undertaken using basic geoprocessing operations (table S7) in the ArcMAP 10.4.1 software interface, many of which are included in the Analysis and Spatial Analyst toolboxes (*67*), as well as through programmed operations using the R language (data S2 and S3) (*68*). Once all road, railroad, and pathway input data were organized and ready for modeling, we calculated the Euclidean distances from all of the identified road, railroad, and path features (three separate layers per infrastructure type and a fourth layer with all three infrastructure types) at a spatial resolution of 250 m by 250 m for all of our study area using the Euclidean Distance function in ArcMAP 10.4.1 software interface (fig. S8 and data S1) (*67*). For general area measurements, spatial information from the resulting raster layers was converted to vector shapefiles for 1- and 5-km RLRL areas, and thus, we were able to calculate the territorial coverage and other statistics outlined in Results. Raster layers were maintained for the statistical analysis.

### Statistical analysis

We ran a series of binomial logistic regression models at the biome level in the R language using the glm function with logit link (Binomial Logistic Regression Model) from the base R stats package (*68*). Starting with all classified land cover cells from Project MapBiomas (*65*), a filter was implemented to remove all dense urban zones (dense urban built environment, such as the urbanized zones of São Paulo or Rio de Janeiro), thus leaving each biome’s rural areas with all land cover types. Then, abiotic land cover and features such as water, rocky outcrops, and sand dunes (*65*) were also filtered out to generate the sample population as the study area from which to run the logistic regression models. This was done to identify the areas that have already been deforested or could be deforested in the future as the binomial response to regress the independent variables on. With each biome’s rural area sample population identified, six binomial logistic regression models were conducted to test the relationship of rural native vegetation land cover and anthropic land use types in relation to their distance from transportation infrastructure in all of Brazil’s biomes. All binomial logistic regression models had the following general format

$$\text{Logit}(Y)=\text{natural}\;\text{log}(\text{odds})=\text{ln}(p/(1-p))=\alpha +\beta_{1}x_{1}$$

where

$$P=\text{Probability}\;(Y=1\;|\;X=x\;(a\;\text{specific value of}\;x)=\frac{e^{\alpha +\beta_{1}x_{1}}}{1+e^{\alpha +\beta_{1}x_{1}}}$$

For all biome-specific logistic regression models, the dependent response of *Y =* 1 is always represented by native vegetation and *Y =* 0 is always represented by anthropic land use, and α is always the intercept of each model.

In the biome-wide base model (model 1): The sample population was *n*_all rural landscapes_ and *x*_1_ is a pixel cell’s distance in kilometers to the closest infrastructure, without differentiation of each cell’s closest infrastructure type, and β_1_ is the coefficient for *x*_1_.

In the biome-wide stratified unprotected model (model 2): The sample population was a stratified subsample of *n*_Unprotected rural landscapes_, and *x*_1_ is a cell’s distance in kilometers to each unprotected cell’s closest infrastructure (without differentiation of infrastructure type), and β_1_ is the coefficient for *x*_1_.

In the biome-wide stratified protected model (model 3): The sample population was a stratified subsample of *n*_LPA − protected rural landscapes_, and *x*_1_ is a cell’s distance in kilometers to each protected cell’s closest infrastructure (without differentiation of infrastructure type), and β_1_ is the coefficient for *x*_1_.

In the biome-wide stratified road model (model 4): The sample population was a stratified subsample of *n*_Road dominated rural landscapes_ in which only cells whose closest infrastructure class is roads, and where *x*_1_ is a cell’s distance in kilometers to the closest road, and β_1_ is the coefficient for *x*_1_.

In the biome-wide stratified railroad model (model 5): The sample population was a stratified subsample of *n*_Railroad dominated rural landscapes_ in which only cells whose closest infrastructure class is railroads, and where *x*_1_ is a cell’s distance in kilometers to the closest railroad, and β_1_ is the coefficient for *x*_1_.

In the biome-wide stratified pathway model (model 6): The sample population was a stratified subsample of *n*_Pathway dominated rural landscapes_ in which only cells whose closest infrastructure class is pathways, and where *x*_1_ is a cell’s distance in kilometers to the closest pathway, and β_1_ is the coefficient for *x*_1_.

For the Amazon, because of the biome’s size and the much larger distance of some of its RLRL areas compared to Brazil’s other five biomes, the sample populations were limited to a distance of 58 km (which is still more distant than any other most distant RLRL cell point in Brazil’s other five biomes). This is because the sheer number of sample data points that stretch up to the Amazon biome’s deepest RLRL area locations caused the logistic regression models into a nonconvergence of only 0 or 1 probabilities for *Y =* 0 and *Y =* 1, respectively. The cutoff of 58 km was chosen not only because it is the third quartile limit of the distribution of the native vegetation land cover class from the nearest infrastructure but also because it allowed for almost all of the anthropic land use sample points to be included in the model (principally only cutting off extreme outliers above the maximum while ensuring that all the minimum to maximum points and some outliers above the maximum were included). Therefore, the upper 25% and outliers of native vegetation land cover were not included in the analyses for the Amazon.

## Supplementary Materials

### This PDF file includes:

Text S1 Figs. S1 to S64 Tables S1 to S70 References
