# Supplementary material for: Conservation opportunities and challenges in Brazil’s roadless and railroad-less areas
Source: Sci Adv. 2022 Mar 4;8(9):eabi5548. doi: 10.1126/sciadv.abi5548 (PMC8896799; doi:10.1126/sciadv.abi5548)
Supplement: Supplementary file 1 — Text S1 Figs. S1 to S64 Tables S1 to S70 References [file sciadv.abi5548_sm.pdf]

Science Advances
AAAS

# Supplementary Materials for

## Conservation opportunities and challenges in Brazil's roadless and railroad-less areas

Trevor R. Tisler*, Fernanda Z. Teixeira, Rodrigo A.A. Nóbrega

*Corresponding author. Email: trtisler@ufmg.br

Published 4 March 2022, *Sci. Adv.* **8**, eabi5548 (2022)
DOI: 10.1126/sciadv.abi5548

**The PDF file includes:**

Text S1
Figs. S1 to S64
Tables S1 to S70
Legends for data S1 to S3
References

**Other Supplementary Material for this manuscript includes the following:**

Data S1 to S3

**Text S1**

**Legally Protected Areas (LPAs).** Brazil's primarily tool for enacting its biodiversity and conservation policies is through the designation of Protected Areas, termed Conservation Units (UCs, for *Unidades de Conservação*) (*76*, *77*). Brazilian UCs have two principal categories: a) Full Protection (PIs, for *Proteção Integral*) which correspond to International Union for Conservation of Nature (IUCN) protected area categories I, II and III; and b) Sustainable Use (USs, for *Uso Sustentável*) which correspond to IUCN protected area categories IV, V, and VI (*76*, *78*). Furthermore, these two types of UCs can be instated by federal, state or municipal governments in Brazil as outlined in Federal Law n. 9.985 of July 18, 2000 (*79*). To identify synergies between UCs and RLRL areas we used official spatial data from Brazil's Ministry of the Environment's (MMA) National Registry of Conservation Units (CNUC, for *Cadastro Nacional de Unidades de Conservação*) which is the principal database for spatial data for UCs administered at all three levels of government in Brazil (*38*) (fig. S5).

Indigenous Territories (TIs, for *Terras Indígenas*) in Brazil also play an important role in conserving native vegetation cover and biodiversity, especially in areas with high deforestation pressure (*78*, *80–84*). Furthermore, Amazonian TIs are the guardians of extensive above-ground carbon reserves that if lost could destabilize global climate patterns (*85*). Additionally, Brazil's Maroon Community Territories (*Quilombos*) have also been shown to contribute to native vegetation protection (*82*, *86*) and in general have low deforestation rates (*87*). Given the contributions of TIs and Quilombos to biodiversity conservation both were included in our study as LPAs alongside UCs (fig. S5). However, it is important to remember that TIs and Quilombos are governed by different legislation from UCs. Official spatial data for TIs comes from Brazil's National Foundation for Indigenous Peoples (FUNAI, for *Fundação Nacional do Índio*) (*39*). Official spatial data for Quilombos comes from Brazil's National Institute for Colonization and Agrarian Reform (INCRA, for *Instituto Nacional de Colonização e Reforma Agrária*) (*40*).

It is important to note that the process for legally recognizing TIs and Quilombos and their land rights in Brazil is a multistep, complex and bureaucratic process (*88–94*). Even after the territorial limits of a TI or Quilombo are identified, the community faces many additional legal steps (*91*) before obtaining land rights to that territory and the important legal and constitutional protections that come with secured land rights (*95*). Therefore, based on this important legal difference in land rights status, identified TIs and Quilombos from the input data sources were split between two categories, (i) those that enjoy full legal and constitutional rights through regularization and land titles and, (ii) those that still do not enjoy full legal and constitutional rights as they have not completed the administrative process of land regularization and do not hold land titles.

In the principal scope of this study, only fully regularized TIs and Quilombos (with secured land titles recognized by the state and entitled to constitutional protection) are used in the spatial analysis for protection status for native vegetation in relation to RLRL areas. However, TIs and Quilombos that are still awaiting land regularization could provide legally sound conservation opportunities that would result in biodiversity conservation and that would be legally guaranteed to continue in the future if the land rights of these territories were to be completely formalized (*95*). Furthermore, the Brazilian Forum on Climate Change (henceforth FBMC, for *Fórum Brasileiro de Mudança do Clima*), which is sanctioned by Presidential Decree n. 9.082 of June 26, 2017 (*96*) to guide proposals for how Brazil could achieve the successful implementation of its NDC for the Paris Agreement, published their initial proposal action plan in which the FBMC

highlighted that there are many proposed UCs and unregularized TIs waiting for the stroke of a pen to be formally created and regularized. Moreover, Brazil's NDC states the important role of TIs, both regularized and unregularized with delimited boundaries, for sustainable forest management (*25*). If these proposed UCs and unregularized TIs were to be formally created and regularized, then Brazil would advance towards meeting its international commitments in its NDC in a relatively easy and inexpensive manner (*97*). Finally, it is important to note that FUNAI's data included additional areas of potential indigenous territories; however, the area of these territories and defined borders are not identified in the data set and therefore they were not included in our analysis. Thus, there are likely even more conservation opportunities and conflicts with transportation policy that could result from the demarcation and regularization of these specific TIs.

**Priority Areas for Biodiversity Conservation (PABCs)**. MMA has identified Priority Areas for Biodiversity Conservation (PABCs) in all of Brazil's six biomes, and as outlined in MMA Ministerial Order (*Portaria*) n. 463, of December 18, 2018, PABCs have the explicit purpose of informing and guiding environmental PPPs related to: (i) in situ biodiversity conservation, (ii) sustainable use of biodiversity components, (iii) sharing of benefits arising from access to genetic resources and associated traditional knowledge, (iv) research and inventories on biodiversity, (v) recovery of degraded areas and overexploited or threatened species, and (vi) economic valuation of biodiversity (*41*, Art. 1 Sec. I to VI). These areas have been identified and updated in a second revision which was finished and released to the public in December 2018 (*98*). The spatial distribution of PABCs can be divided between two main categories of importance for this study: (i) parts of PABCs that are already protected by UCs, regularized TIs or titled Quilombos, and (ii) parts of PABCs that are not protected by any of the aforementioned legally protected areas (fig. S6). Therefore, for this study only the areas of PABCs that are not already protected by LPAs were included in our analysis. This was done to identify the locations where unprotected RLRL areas also coincide with unprotected PABCs. These identified overlaps thus could be additional conservation and biodiversity protection opportunities for Brazil whether in the form of newly created UCs, or regularized TIs or titled Quilombos if any untitled TIs and Quilombos currently overlap with PABCs. Furthermore, PABCs identified in relation to one biome sometimes overlap with PABCs in relation to other biomes along the ecotone regions in Brazil. Therefore, we corrected area measurements and spatial data structures to prevent double counting of these instances. We used official spatial data for PABCs from MMA (*98*).

**Native Vegetation.** We also evaluated the native vegetation[1] land cover status in our generated RLRL areas, LPAs and MMA's PABCs, and the overlapping areas of these features, by using land-use classification data from the 3rd version of Project MapBiomas (*41*), using classified year 2017. This was done to further identify the current status of RLRL area and LPA overlaps, as well as RLRL areas and overlaps with unprotected PABCs with ecological assets that could benefit from conservation action or restoration. Project MapBiomas data comes in raster format at a spatial

---

[1]Project MapBiomas uses the term 'natural vegetation'; however, the use of this term should be understood as being synonymous to 'native vegetation'. As natural vegetation is used by Project MapBiomas, it excludes other non-vegetated natural features such as: rivers, lakes, naturally barren land, rock or salt flat features. Moreover, Project MapBiomas classifies agricultural, pasture, forestry plantations and other heavily anthropogenic yet vegetated land covers as separate non-natural vegetation. However, as the data is remotely sensed, it does not exclude the possibility that some non-native vegetation is co-classified in the natural vegetation category. Native vegetation is used in Brazil's environmental laws (*vegetação nativa)* and thus referenced as so. In this study natural and native vegetation should be interpreted synonymously for all intents and purposes.

resolution of 30 by 30 meters. Due to computational processing constraints, we converted land cover classifications falling into the native vegetation category to 250 by 250-meter resolution and then transformed the data into vector format (fig. S7).

**MEA Commitments included in Brazil's National Biodiversity Strategy Action Plan Target**. The NBSAP integrates international commitments into national policy for: (i) the Convention on Biological Diversity's (CBD) Aichi Targets and (ii) the Sustainable Development Goals (SDGs) under the 2030 Sustainable Development Agenda (*43*). PABCs are one of Brazil's key strategy tools for working towards achieving Aichi Biodiversity Targets 2, 7, 11, 14 and 15 (*43*) and as part of the country's action plan for achieving SDGs, specifically SDG 15 (*23, 43*). As such, work towards both international commitments has been integrated into national level policy in the country's National Biodiversity Strategy and Action Plan (NBSAP) which was submitted to the CBD in 2017 (*43*). We have identified the following National Target Highlights from Brazil's NBSAP that was submitted to the CBD that are relevant for our analysis based on RLRL areas, LPAs, PABCs and remaining native vegetation cover (*43*). However, for quantification purposes our analysis only makes measurements for analyzing Brazil's progress for achieving Targets 11 and 15, while the other targets are important for consideration.

**National Target 2**: By 2020, at the latest, biodiversity values, geo-diversity values, and socio-diversity values have been integrated into national and local development and poverty reduction and inequality reduction strategies, and are being incorporated into national accounting, as appropriate, and into planning procedures and reporting systems.

**National Target 7**: By 2020 the incorporation of sustainable management practices is disseminated and promoted in agriculture, livestock production, aquaculture, silviculture, extractive activities, and forest and fauna management, ensuring conservation of biodiversity.

**National Target 11**: By 2020, at least 30% of the Amazon, 17% of each of the other terrestrial biomes, and 10% of the marine and coastal areas, especially areas of particular importance for biodiversity and ecosystem services, are conserved through protected areas foreseen under the SNUC Law and other categories of officially protected areas such as Permanent Protection Areas, legal reserves, and indigenous lands with native vegetation, ensuring and respecting the demarcation, regularization, and effective and equitable management, so as to ensure ecological interconnection, integration and representation in broader landscapes and seascapes.

**National Target 14**: By 2020, ecosystems that provide essential services, including services related to water, and contribute to health, livelihoods and well-being, are restored and safeguarded, taking into account the needs of women, traditional peoples and communities, indigenous peoples and local communities, and the poor and vulnerable.

**National Target 15**: By 2020, ecosystem resilience and the contribution of biodiversity to carbon stocks has been enhanced through conservation and restoration actions, including restoration of at least 15% of degraded ecosystems, prioritizing the most degraded biomes, hydrographic regions and ecoregions, thereby contributing to climate change mitigation and adaptation and to combatting desertification.

**National Target 17**: By 2014, the national biodiversity strategy is updated and adopted as policy instrument, with effective, participatory and updated action plans, which foresee periodic monitoring and evaluation.

**National Target 18**: By 2020, the traditional knowledge, innovations and practices of indigenous peoples, family rural producers and traditional communities relevant for the conservation and sustainable use of biodiversity, and their customary use of biological resources, are respected, in accordance with their uses, customs and traditions, national legislation and relevant international commitments, and fully integrated and reflected in the implementation of the CBD, with the full and effective participation of indigenous peoples, family rural producers and traditional communities, at all relevant levels.

**Fig. S1.**

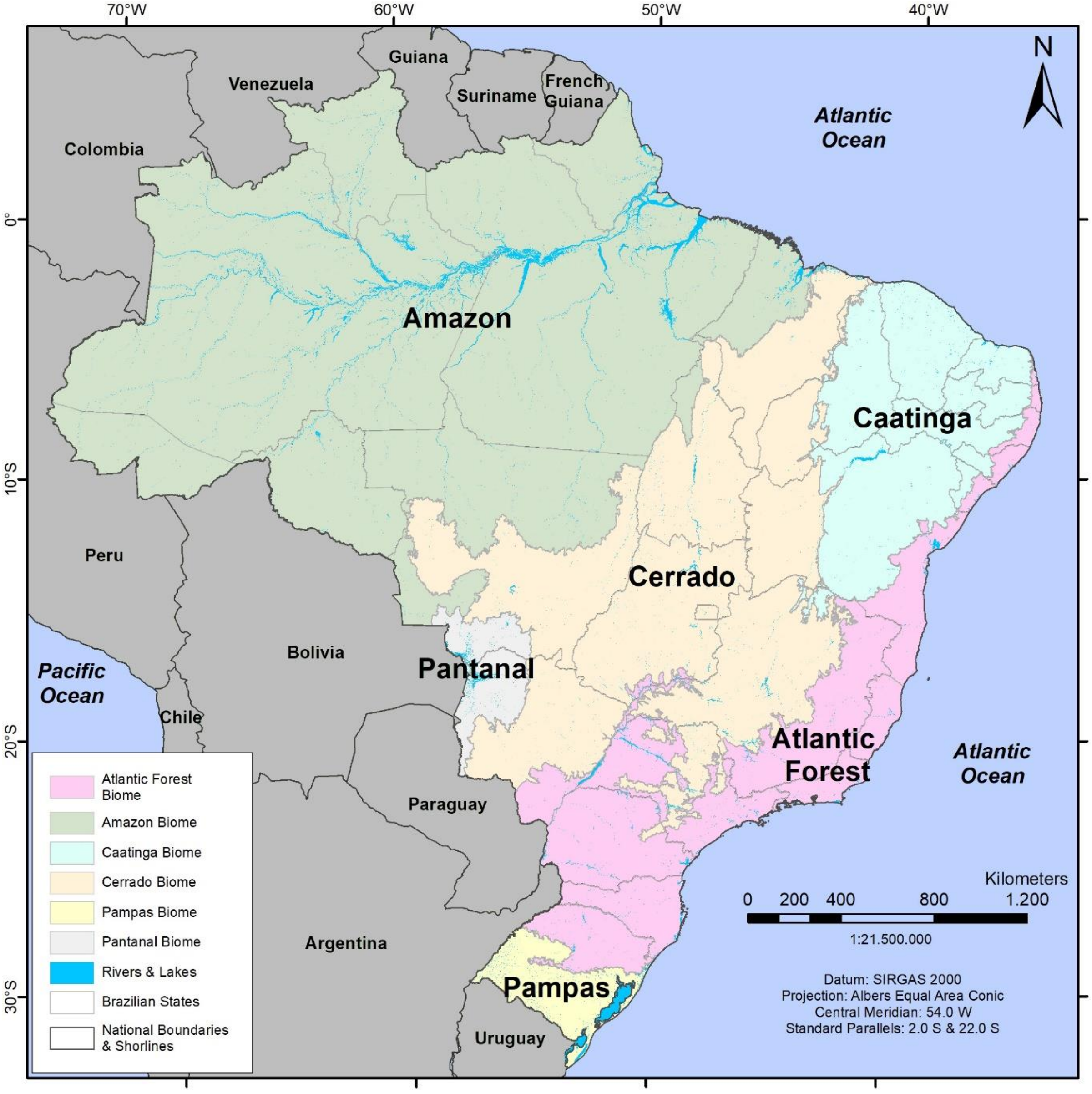


General Study Area (Continental Brazil and Terrestrial Biomes)

**Fig. S2.**

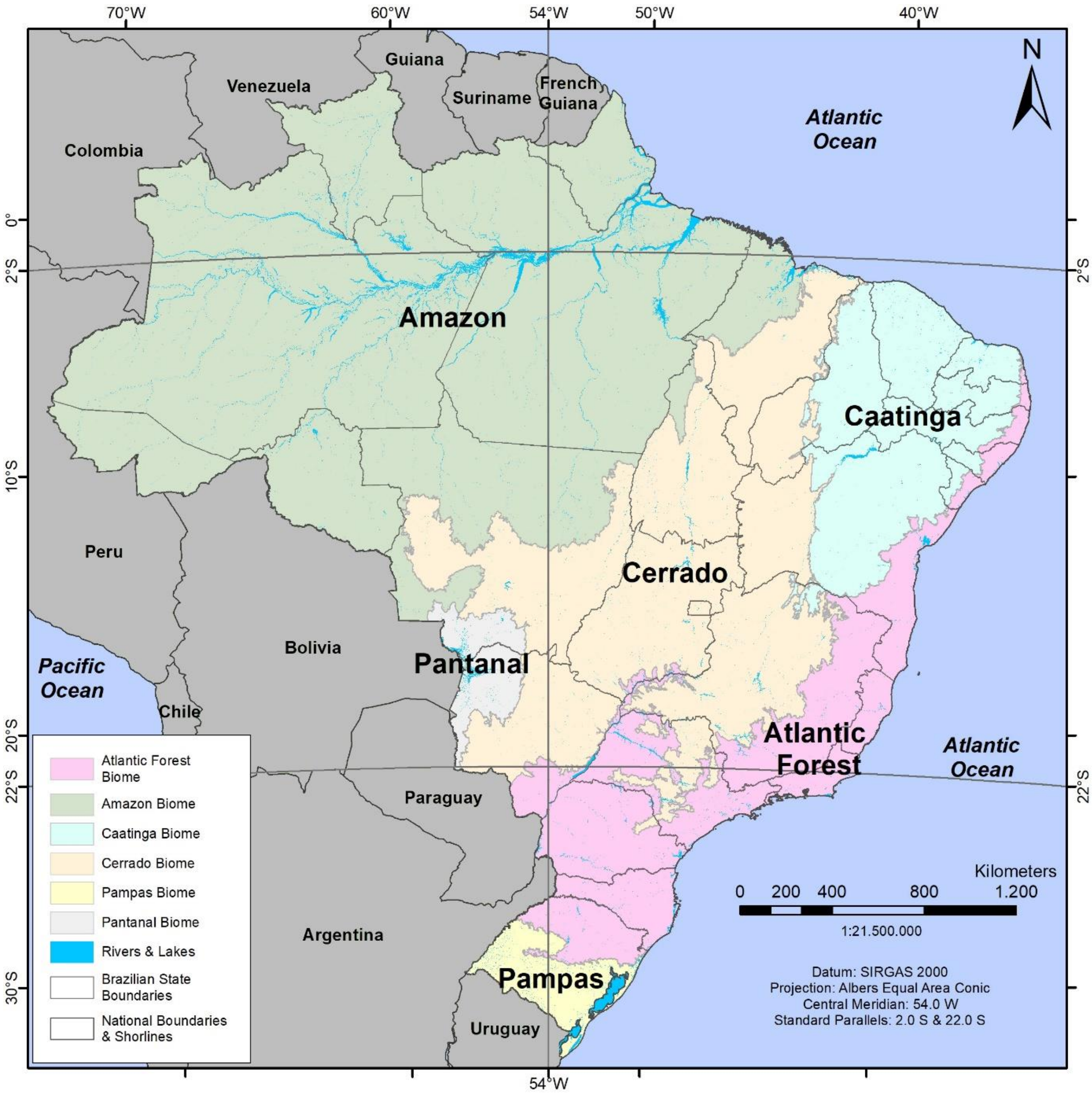


IBGE Projection Configurations on Study Area

**Fig. S3.**

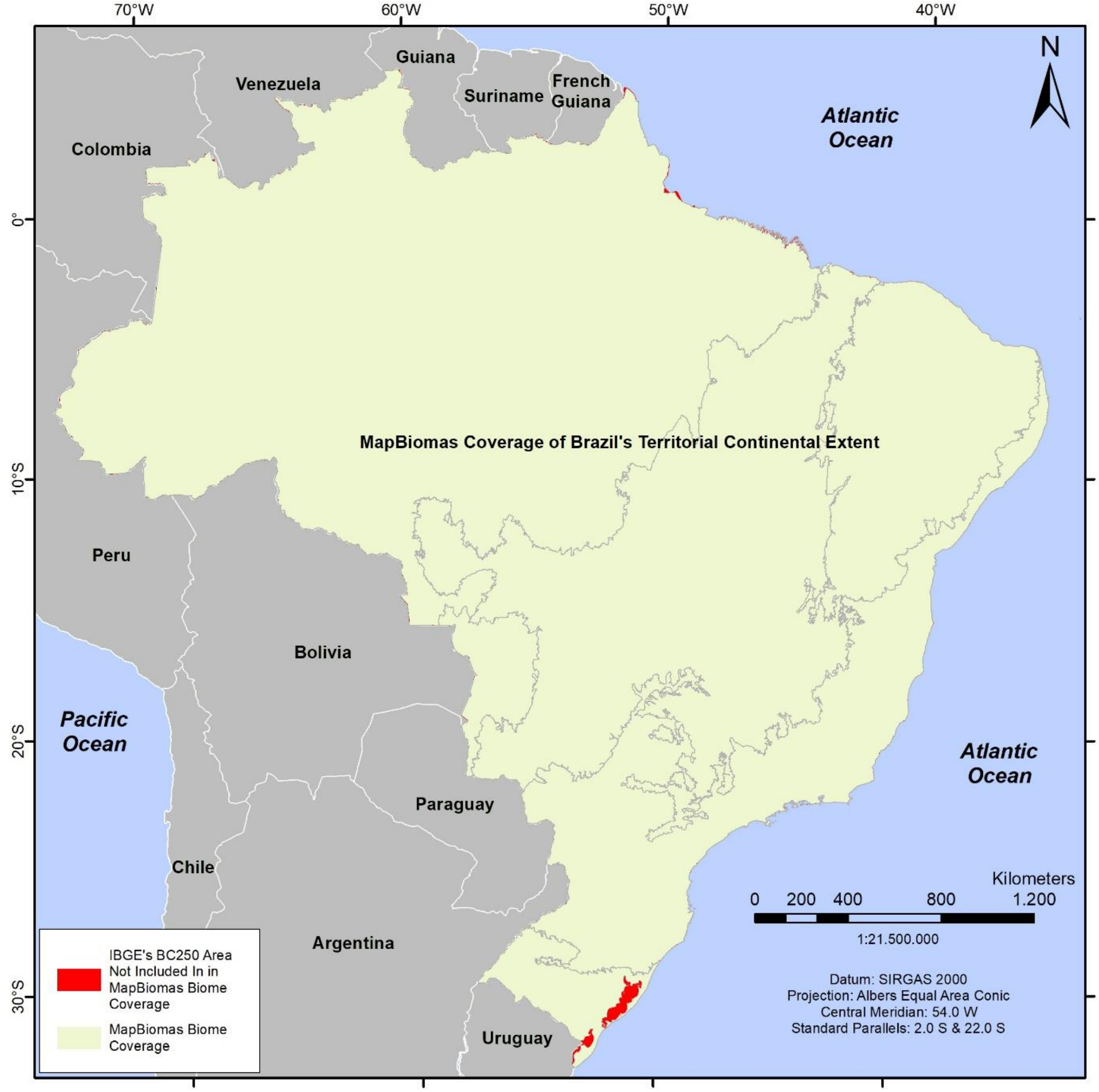


Highlighted Area Coverage Differences between IBGE's BC250 National Territorial Coverage and Rosa & MapBiomas' (2016) Adaptation of Brazil's Terrestrial Biome Coverage.

**Fig. S4.**

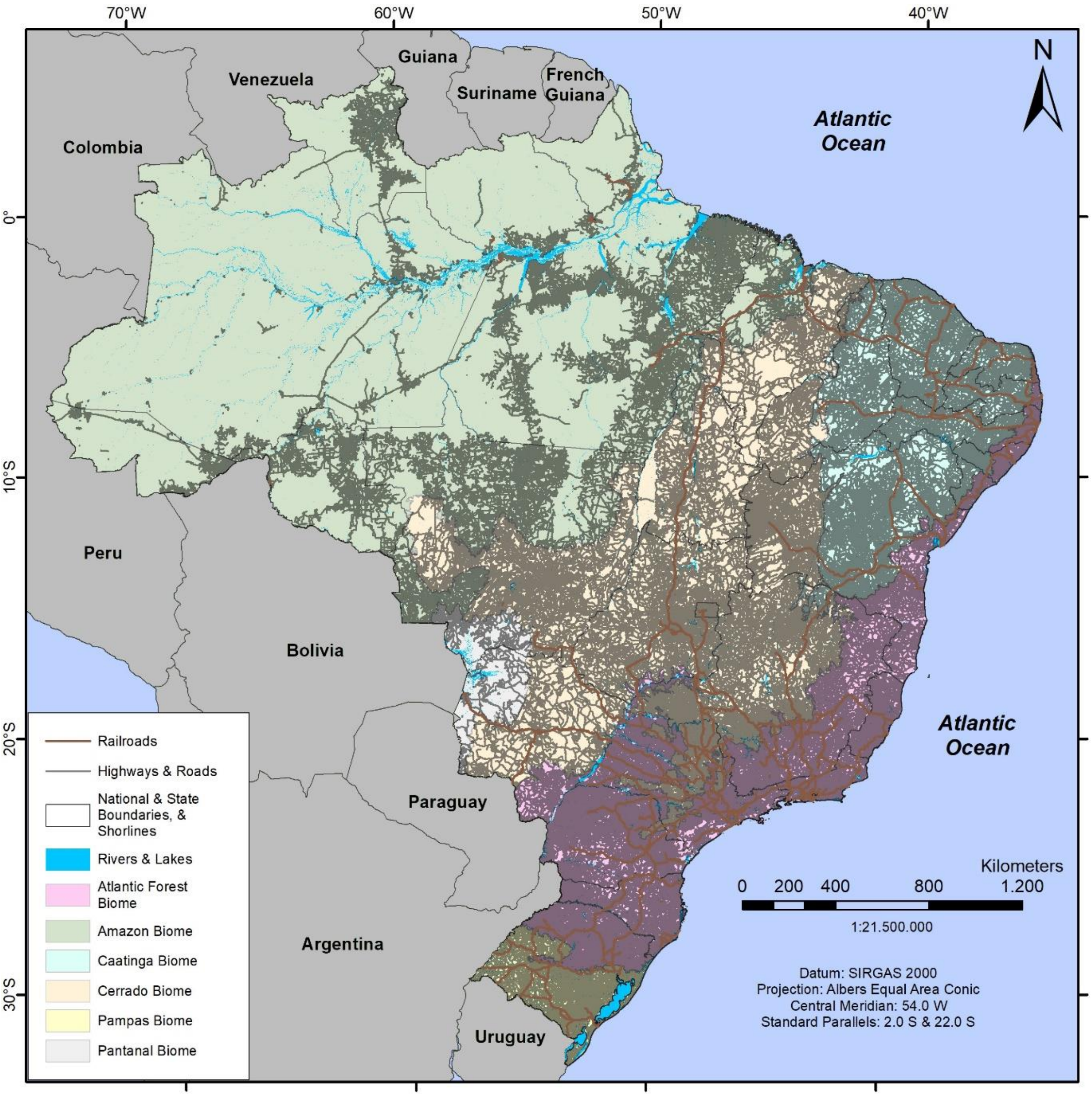


Road and Railroad Input Features for the RLRL area Model.

**Fig. S5.**

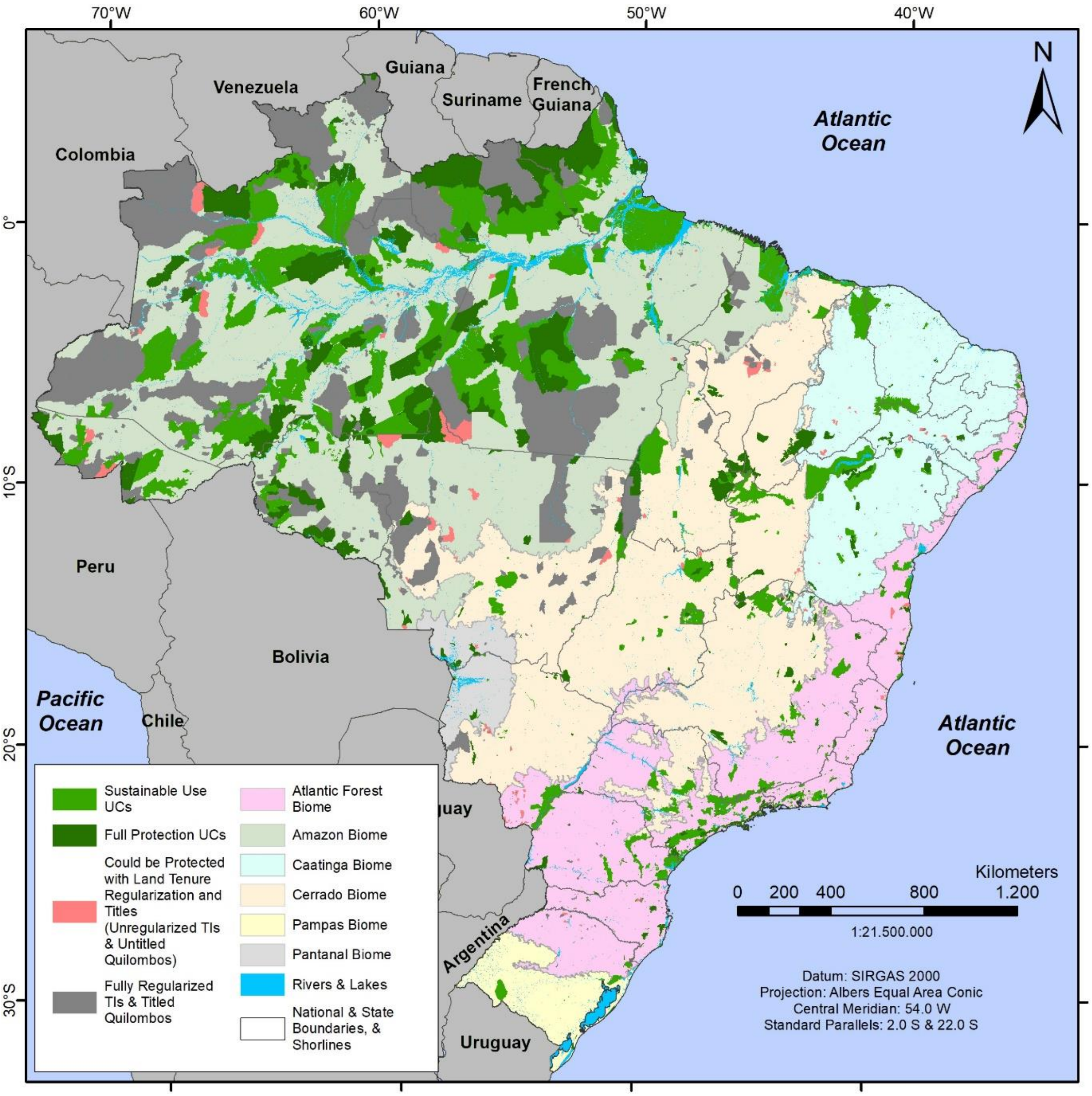


Legally Protected Areas (LPAs) which include Protected Area Conservation Units (UCs – in Dark Green *strict protection UCs* and in Light Green *sustainable use UCs*), Regularized/Land Titled Indigenous Territories (TIs – in Gray) & Titled Maroon/Quilombo Community Lands (Black). While not included in the analysis, Unregularized/Untitled TIs and Untitled Quilombos are included in this figure (Light Red).

**Fig. S6.**

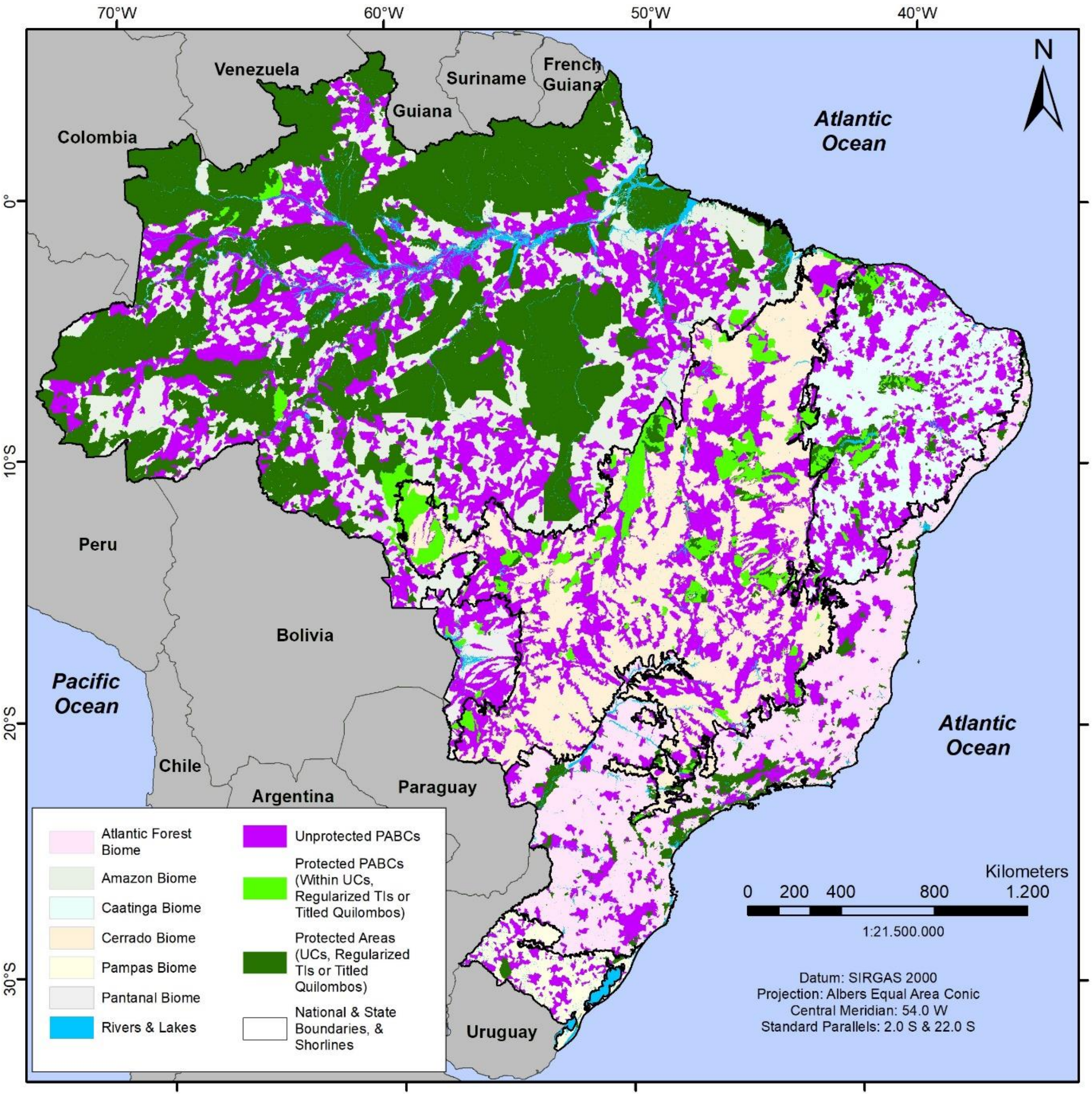


MMA's Second Revision of Priority Areas for Biodiversity Conservation (PABCs). Unprotected PABCs (Purple) were included in our analysis. Already protected PABCs (Light Green) due to their overlaps with Legally Protected Areas (LPAs), were not included in our analysis as they are already fully protected under Brazil's legal framework.

**Fig. S7.**

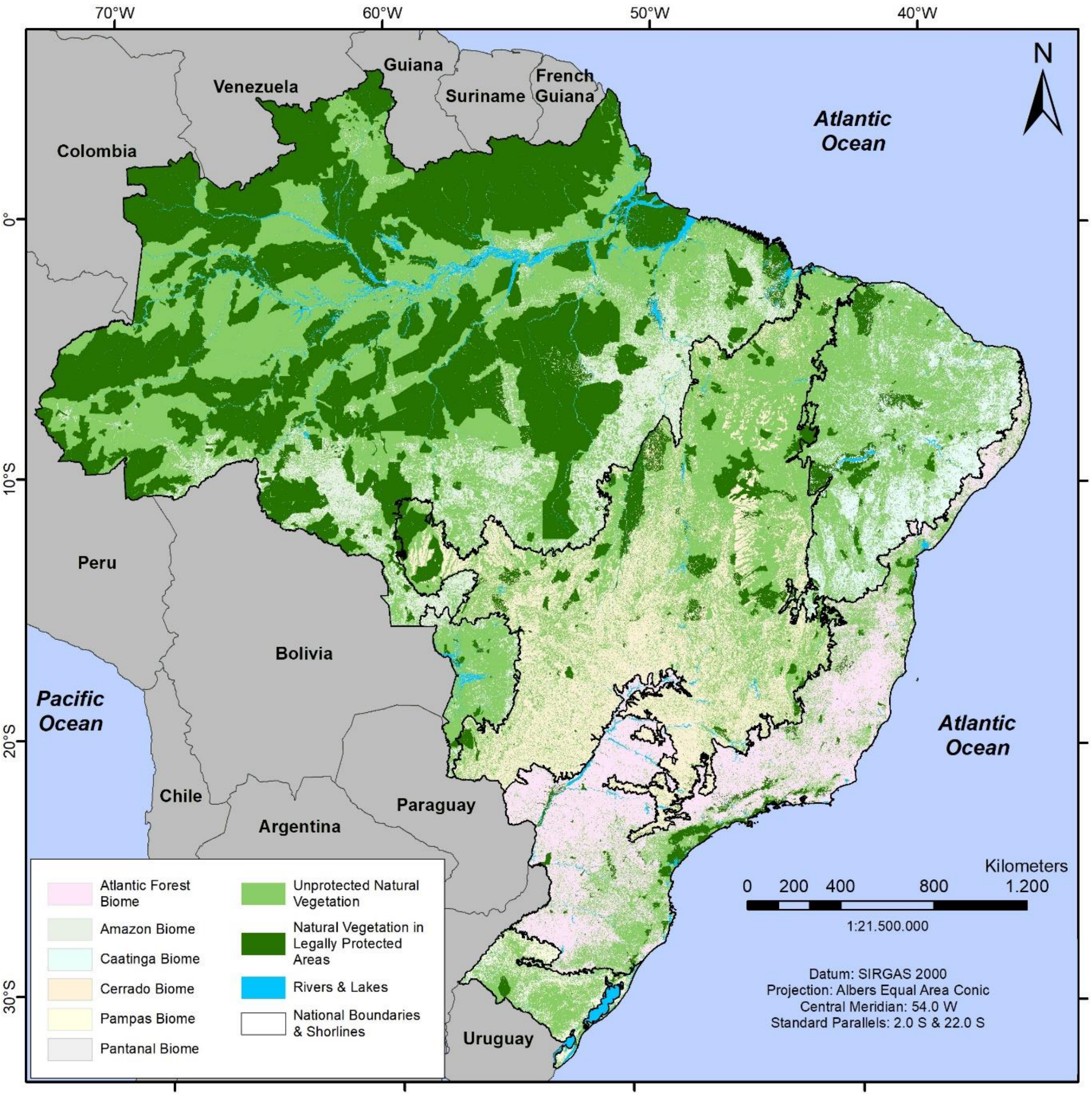


Project MapBioma's Native Vegetation input variable. Native Vegetation protected by a Legally Protected Area (LPA – Dark Green) and Native Vegetation that is unprotected (Light Green).

**Fig. S8.**

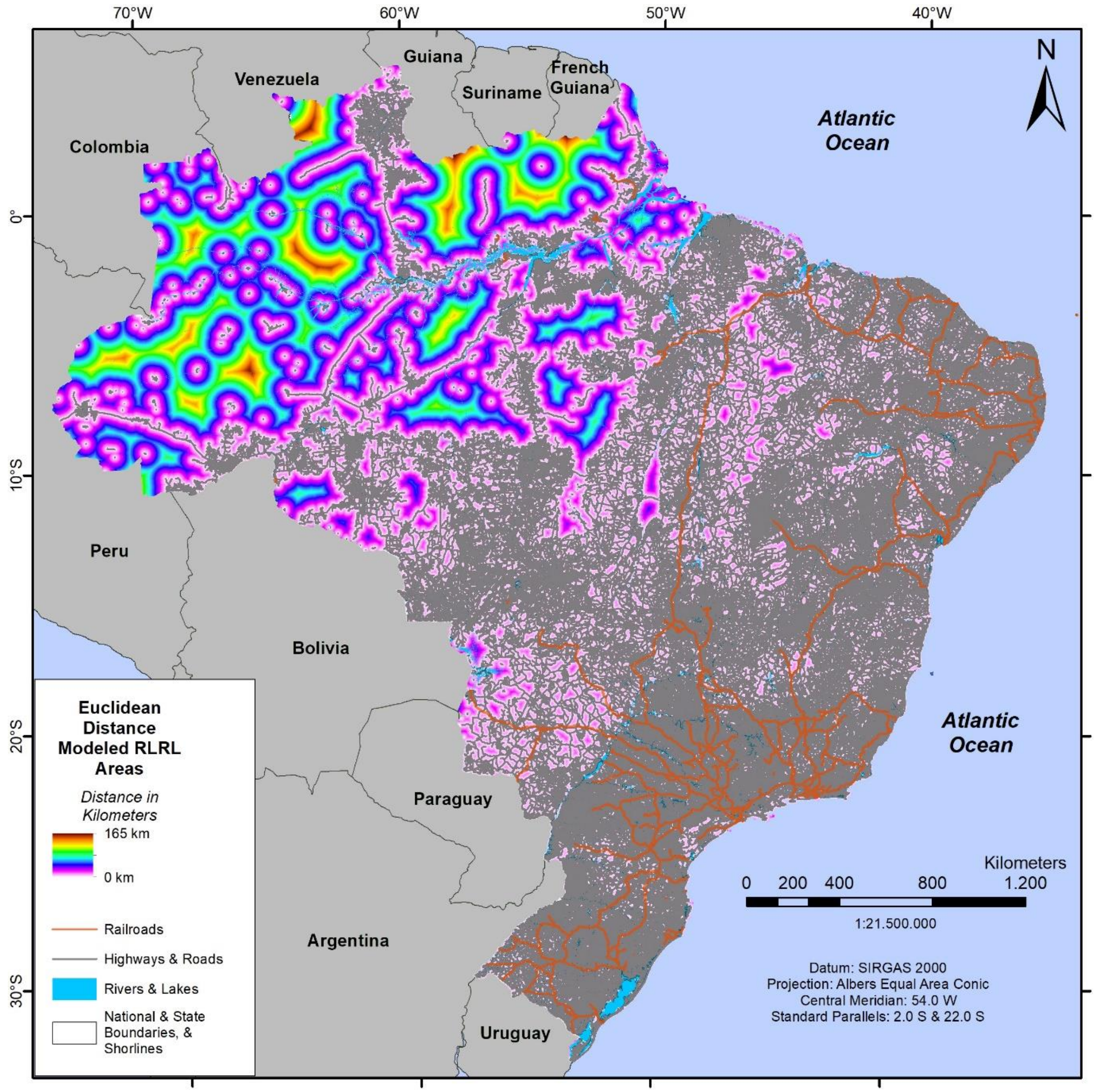


Resulting Euclidian Distance Measurements for Identifying RLRL Areas (using a base raster with 250 x 250-meter resolution.

**Fig. S9.**

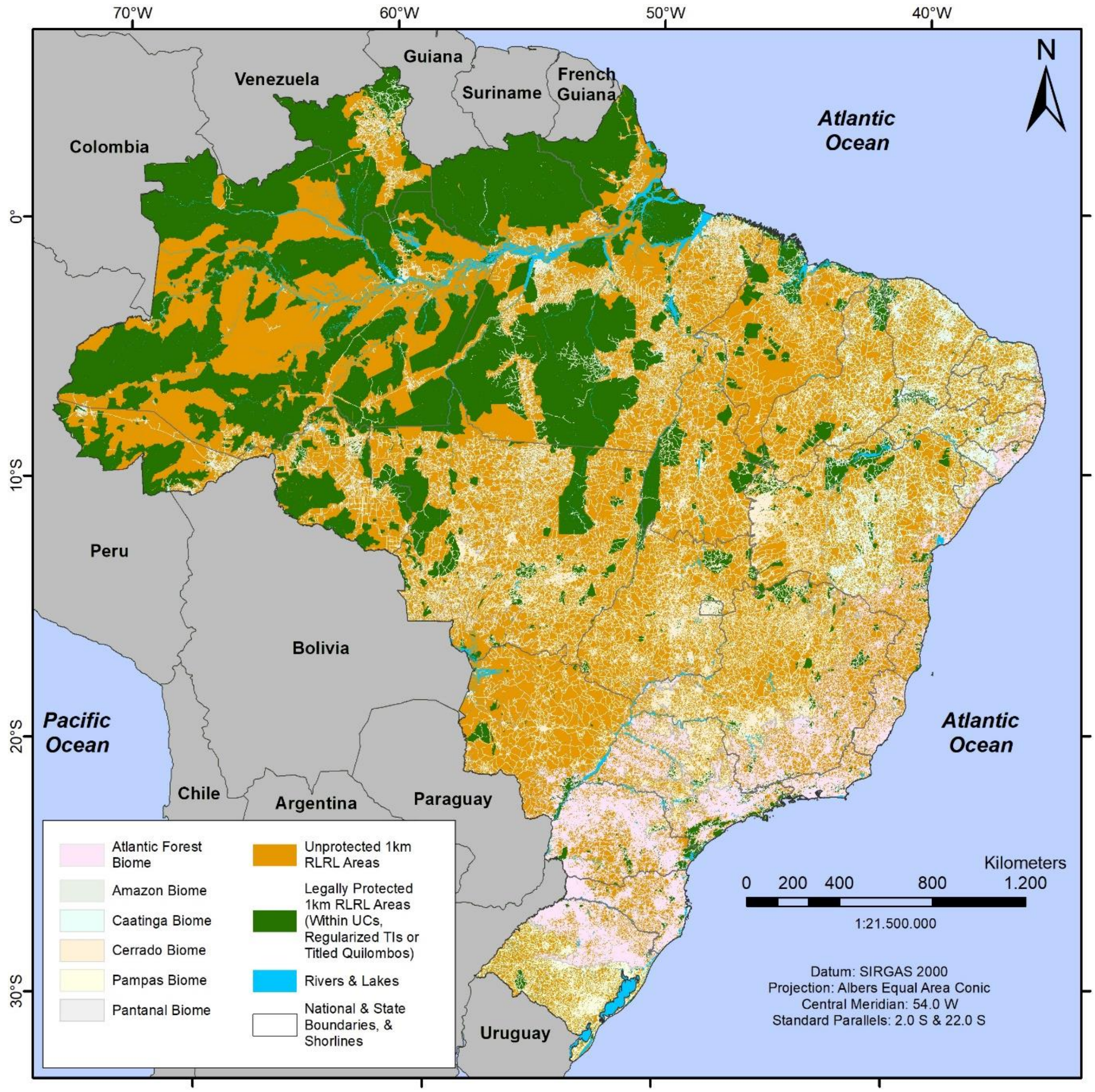


National Level 1km RLRL Areas and Protection Status

**Fig. S10.**

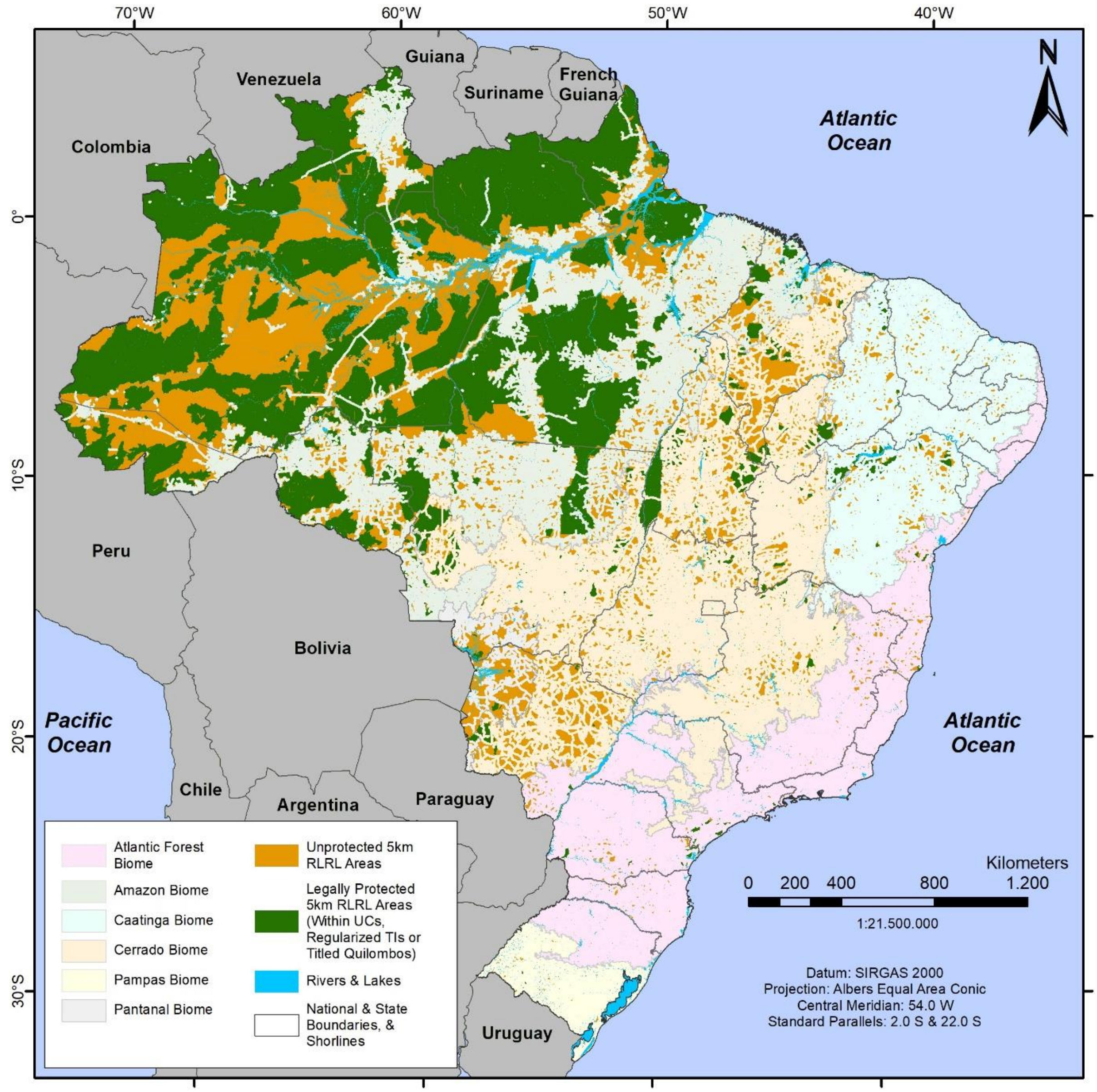


National Level 5km RLRL Areas and Protection Status

**Fig. S11.**

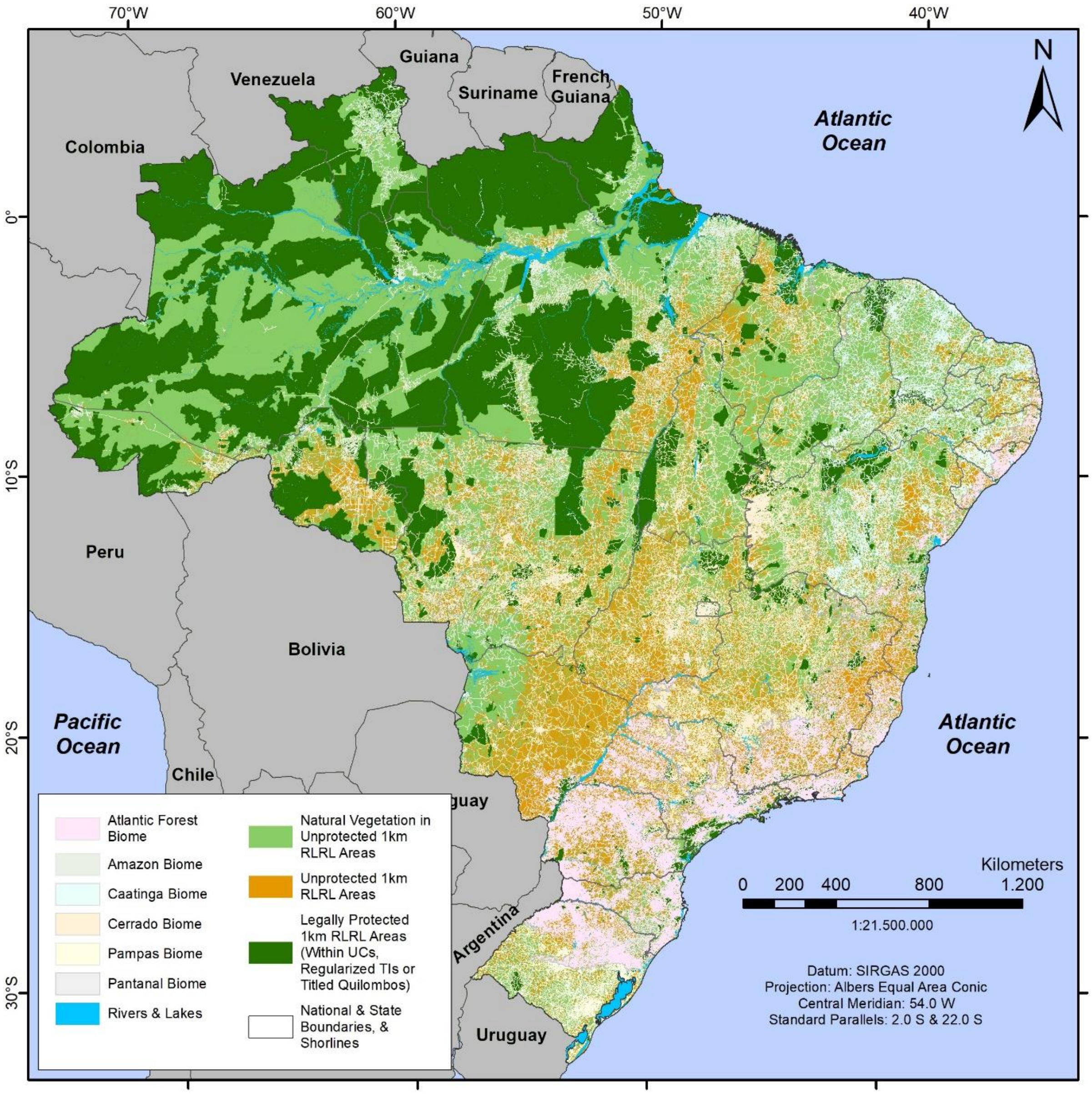


National Level 1km RLRL Areas and Protection Status with Native Vegetation

**Fig. S12.**

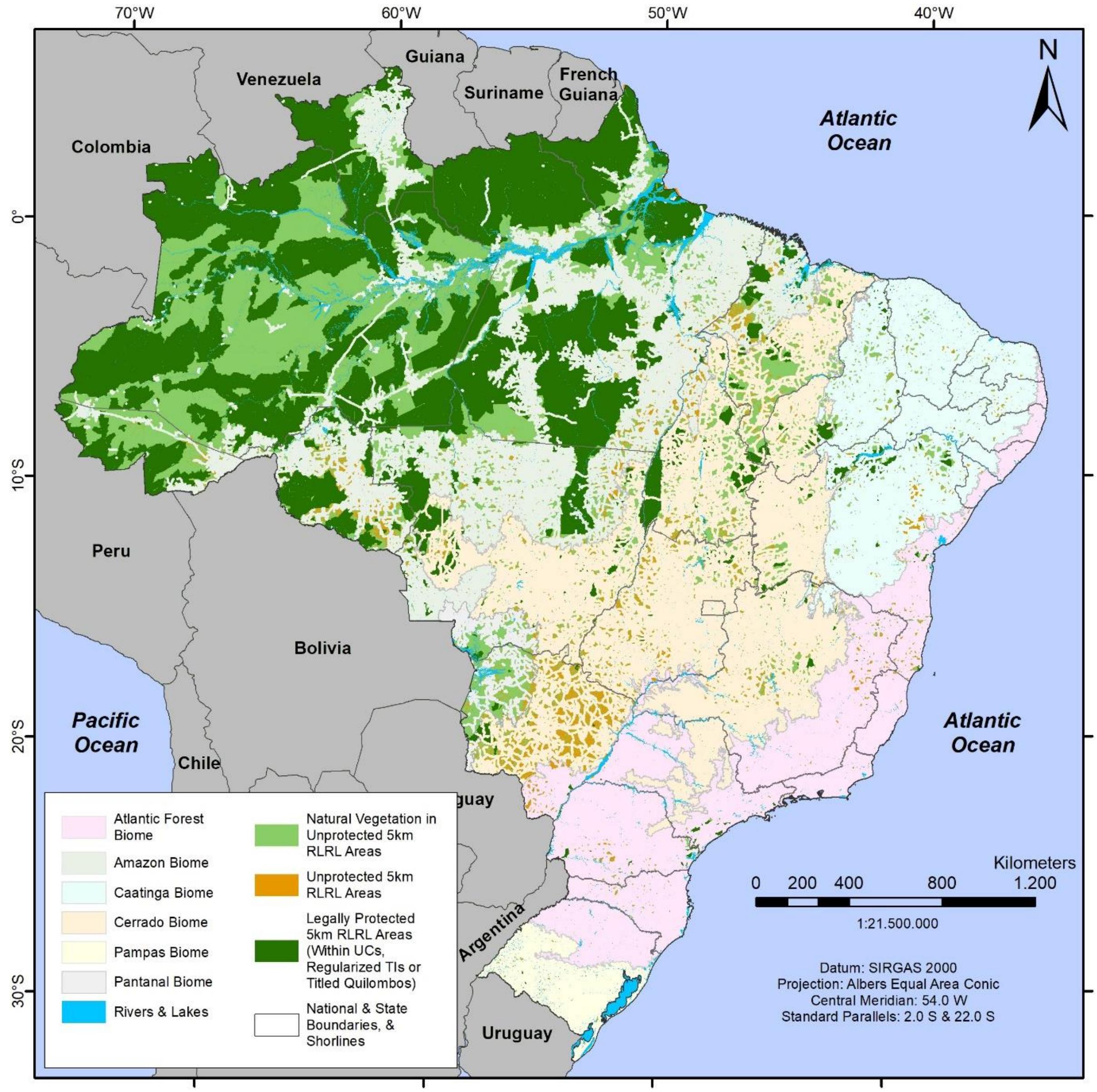


National Level 5 km RLRL Areas and Protection Status with Native Vegetation

**Fig. S13.**

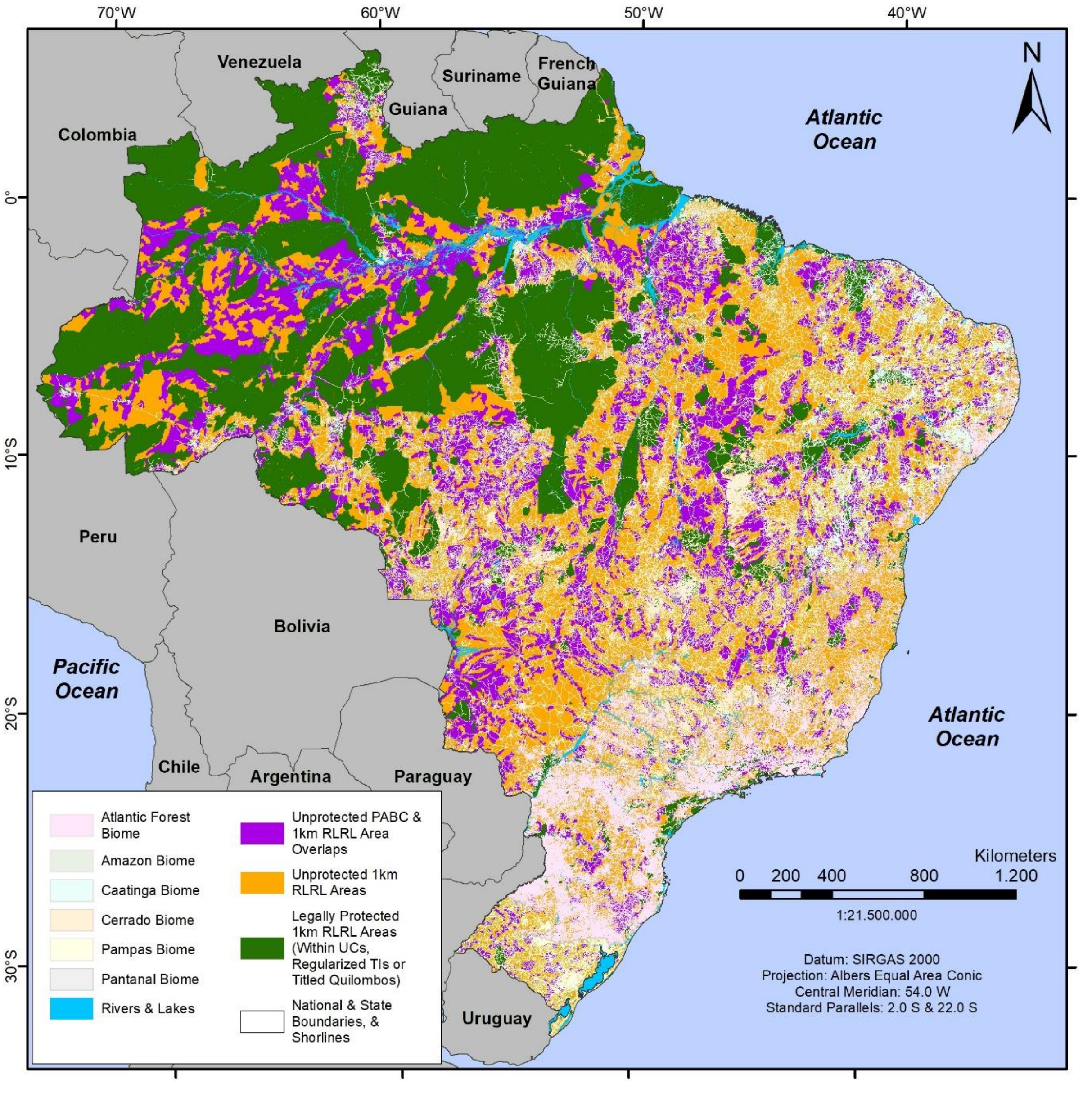


1km RLRL Areas, Legally Protected Areas (LPAs), and Unprotected Priority Areas for Biodiversity Conservation (PABCs)

**Fig. S14.**

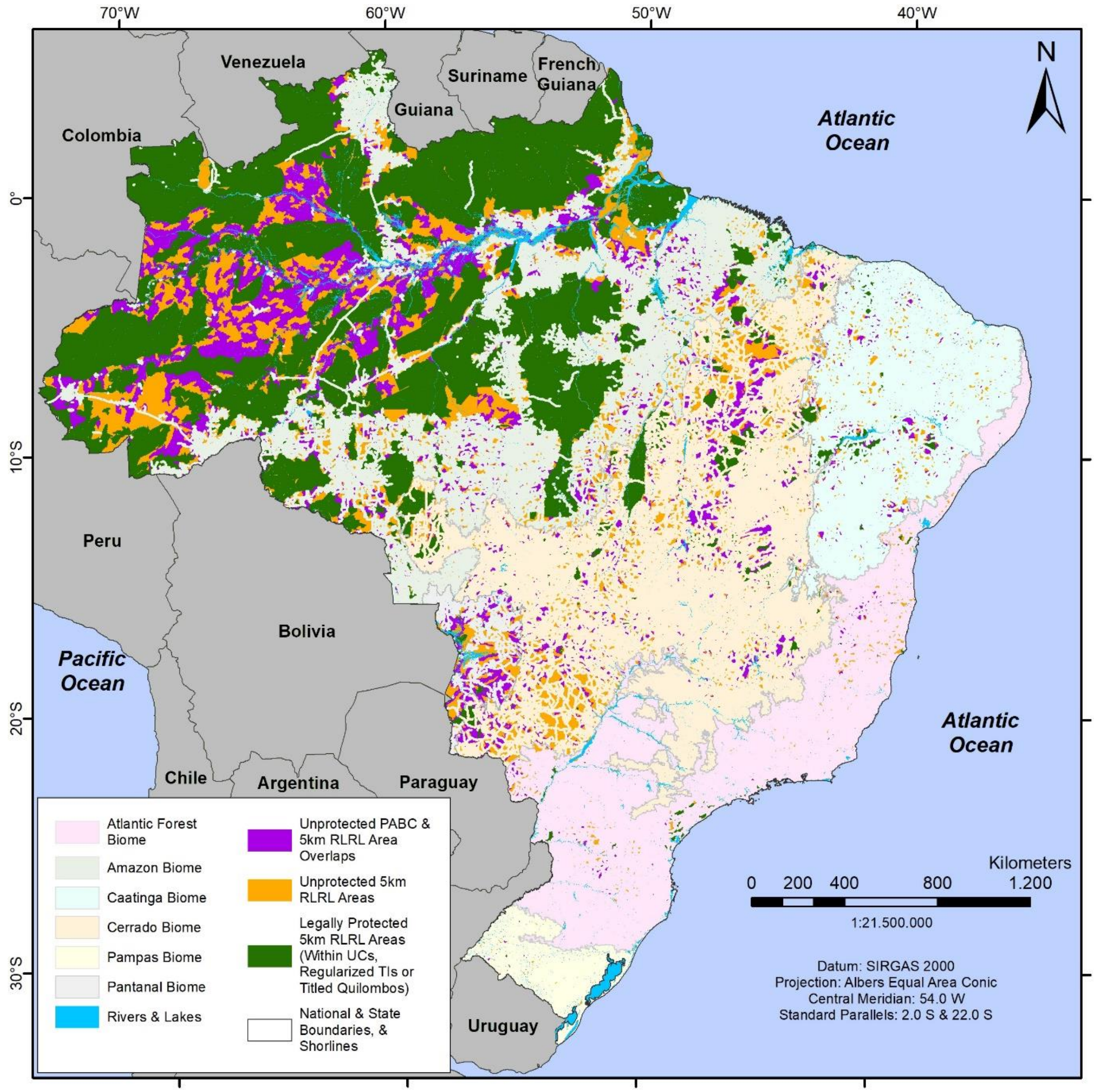


5km RLRL Areas, Legally Protected Areas (LPAs), and Unprotected Priority Areas for Biodiversity Conservation (PABCs)

**Fig. S15.**

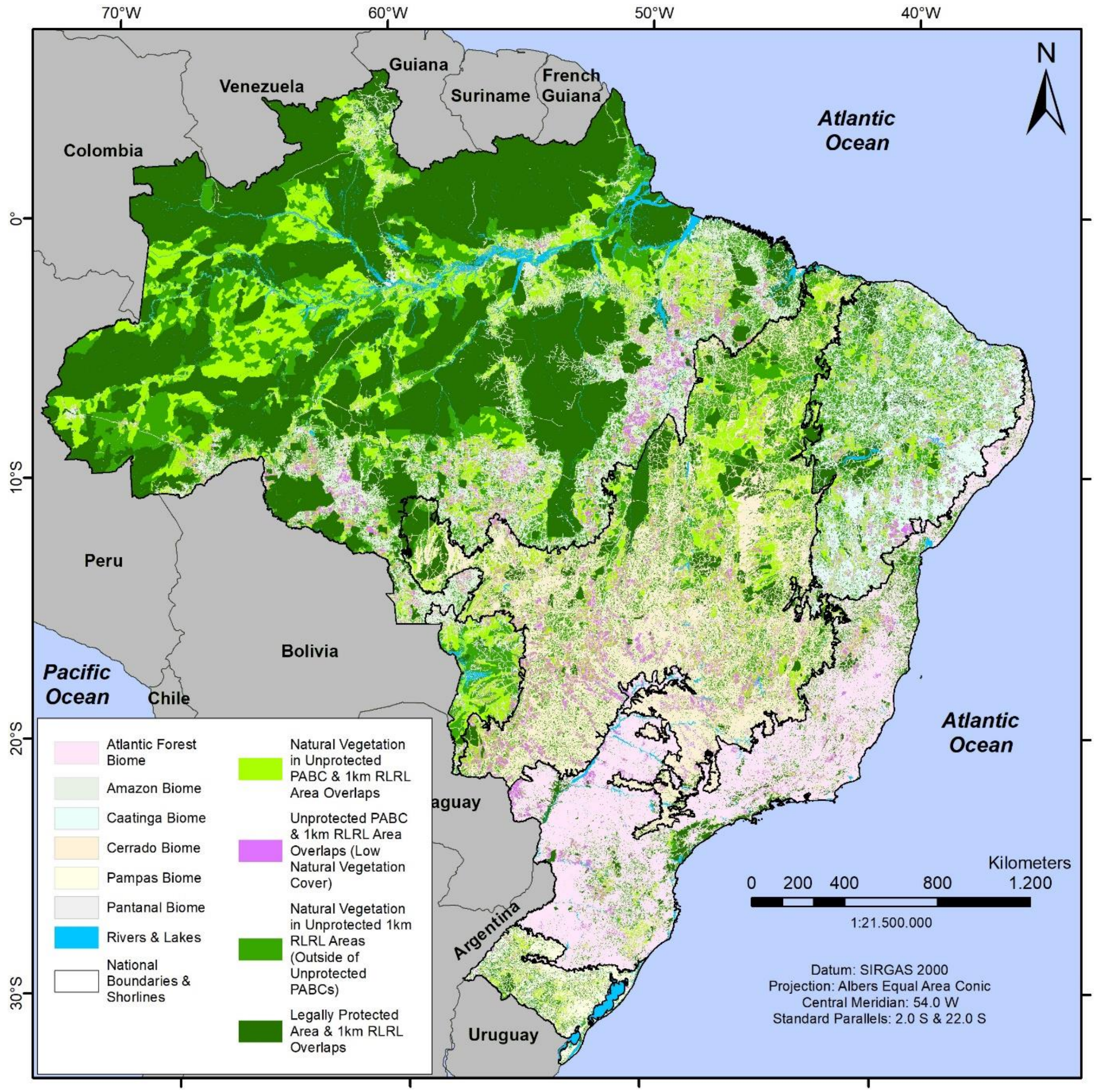


1km RLRL Areas, Native Vegetation, Legally Protected Areas (LPAs), and Unprotected Priority Areas for Biodiversity Conservation (PABCs)

**Fig. S16.**

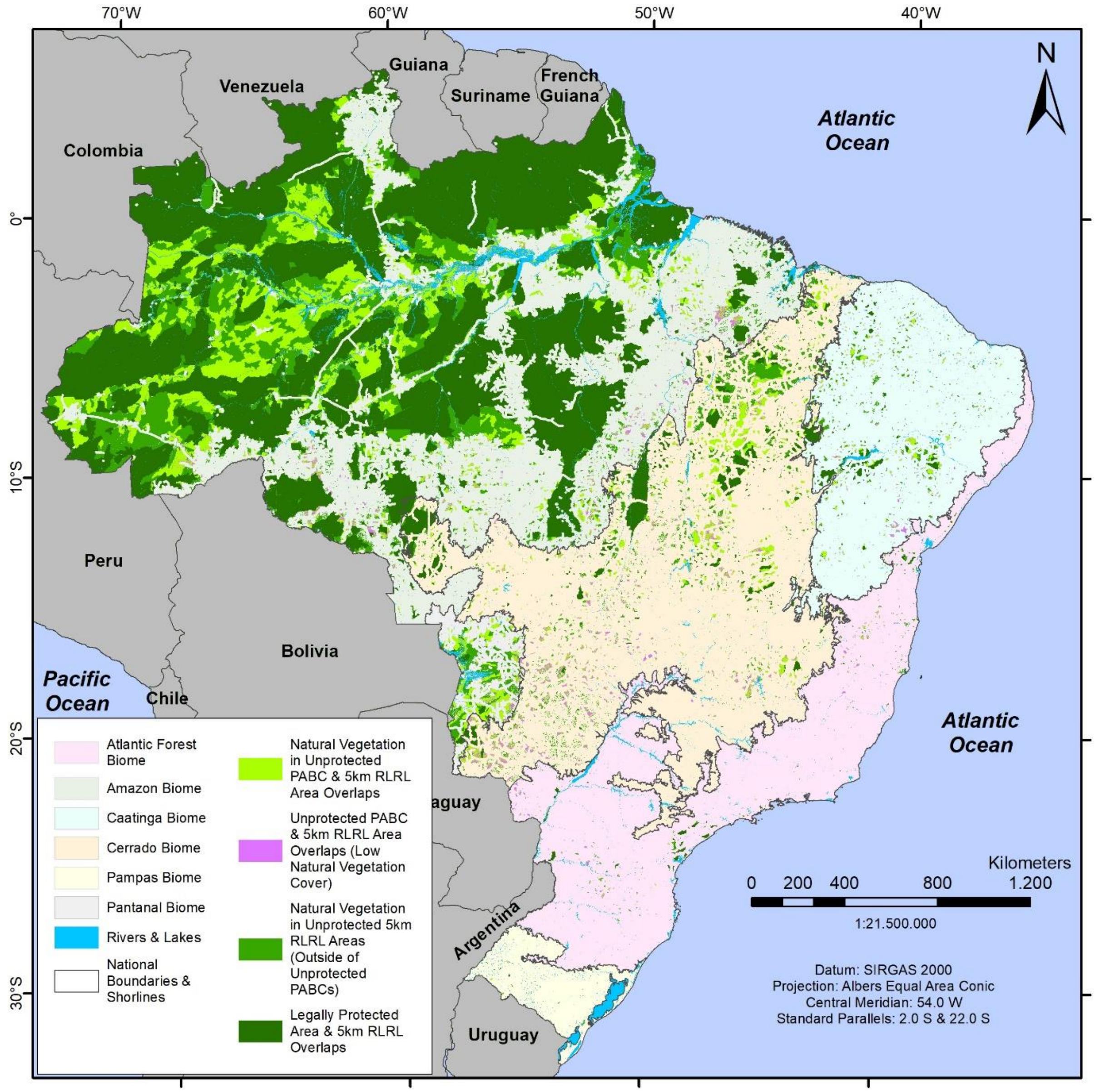


5km RLRL Areas, Native Vegetation, Legally Protected Areas (LPAs), and Unprotected Priority Areas for Biodiversity Conservation (PABCs)

**Fig. S17.**

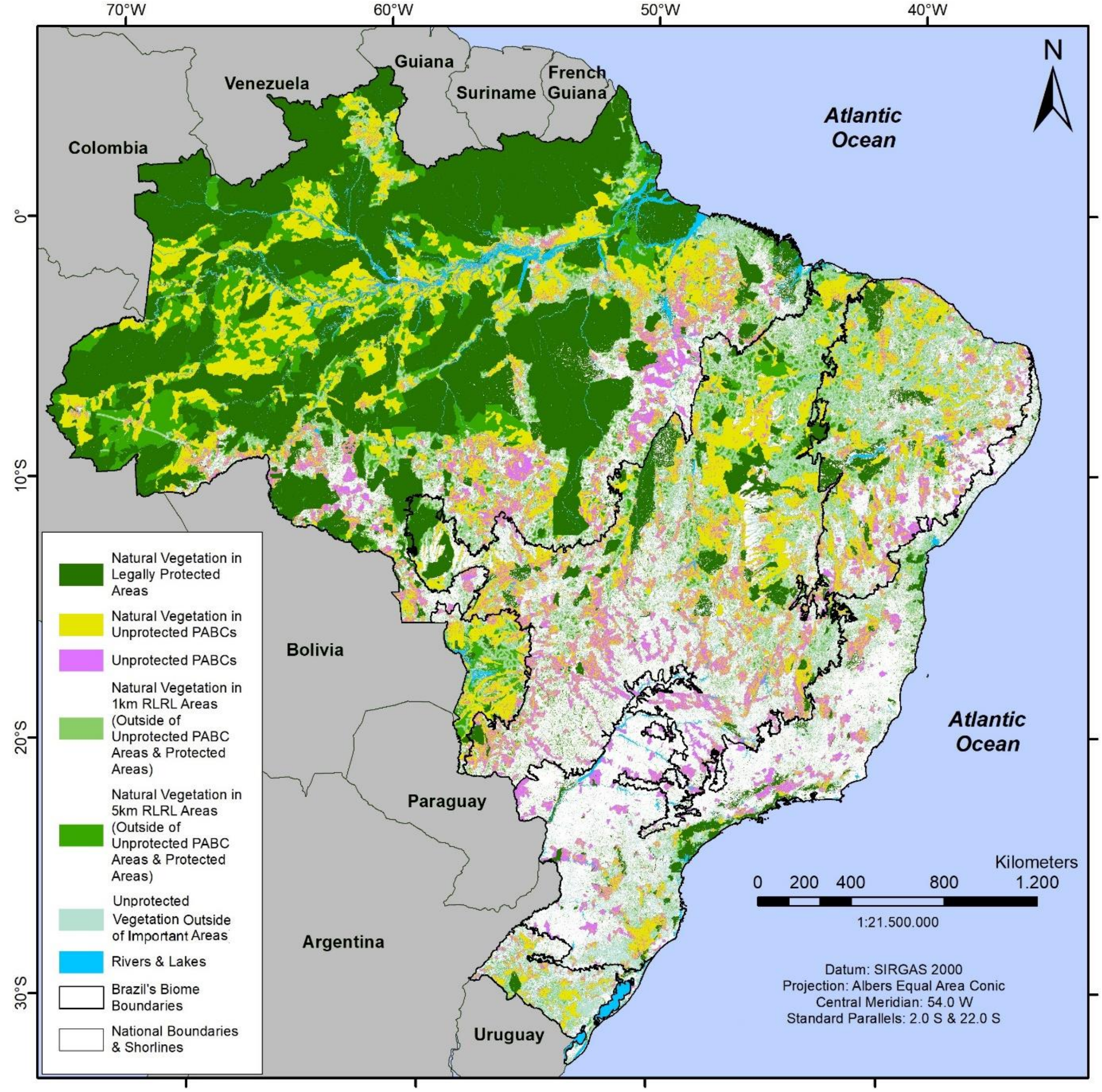


1km RLRL Areas, 5km RLRL Areas, Native Vegetation, Legally Protected Areas (LPAs), and Unprotected Priority Areas for Biodiversity Conservation (PABCs) (With and Without Native Vegetation)

**Fig. S18.**

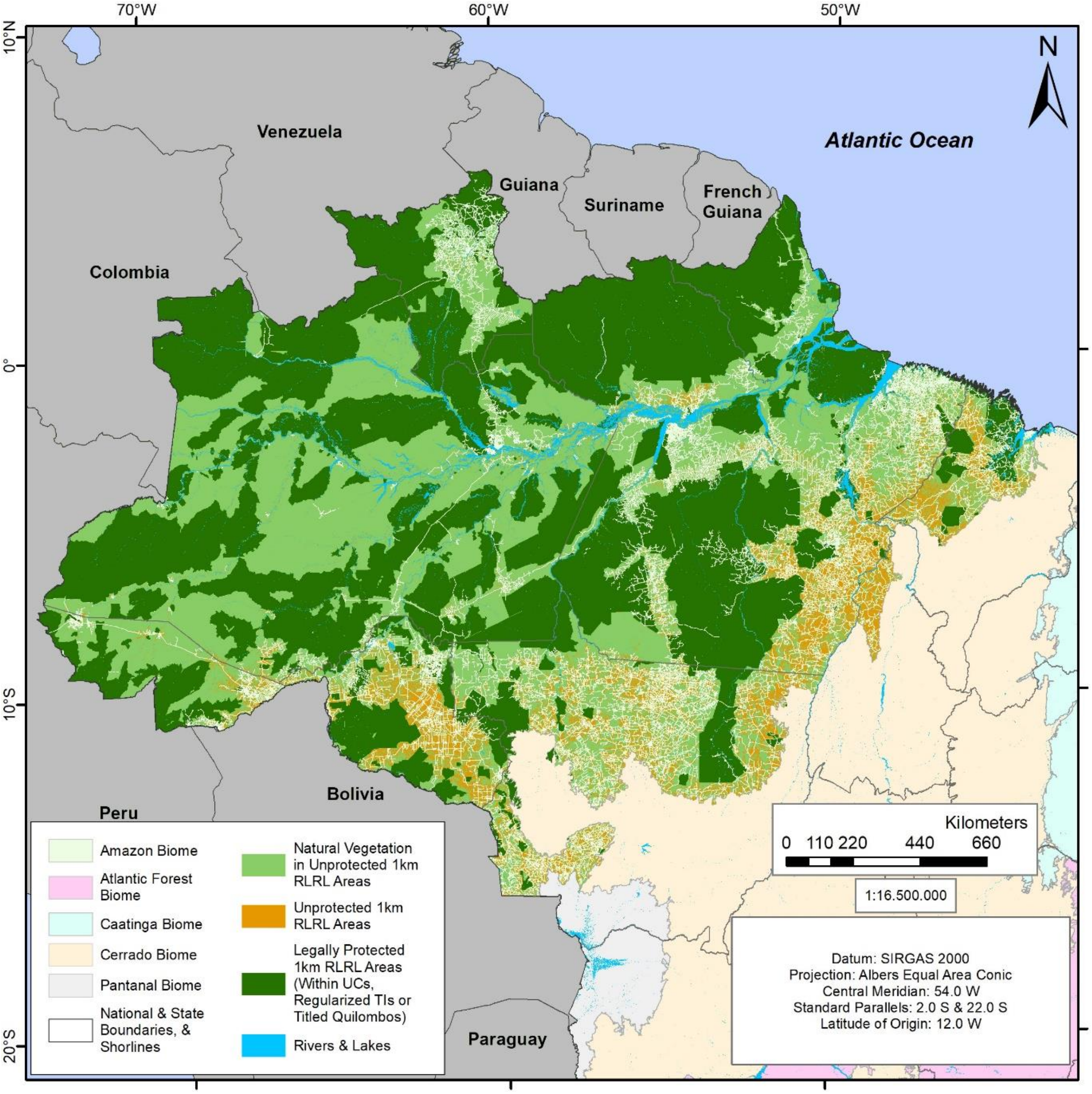


Amazon Biome's 1km RLRL Areas, Legally Protected Areas (LPAs), and Native Vegetation Coverage

**Fig. S19.**

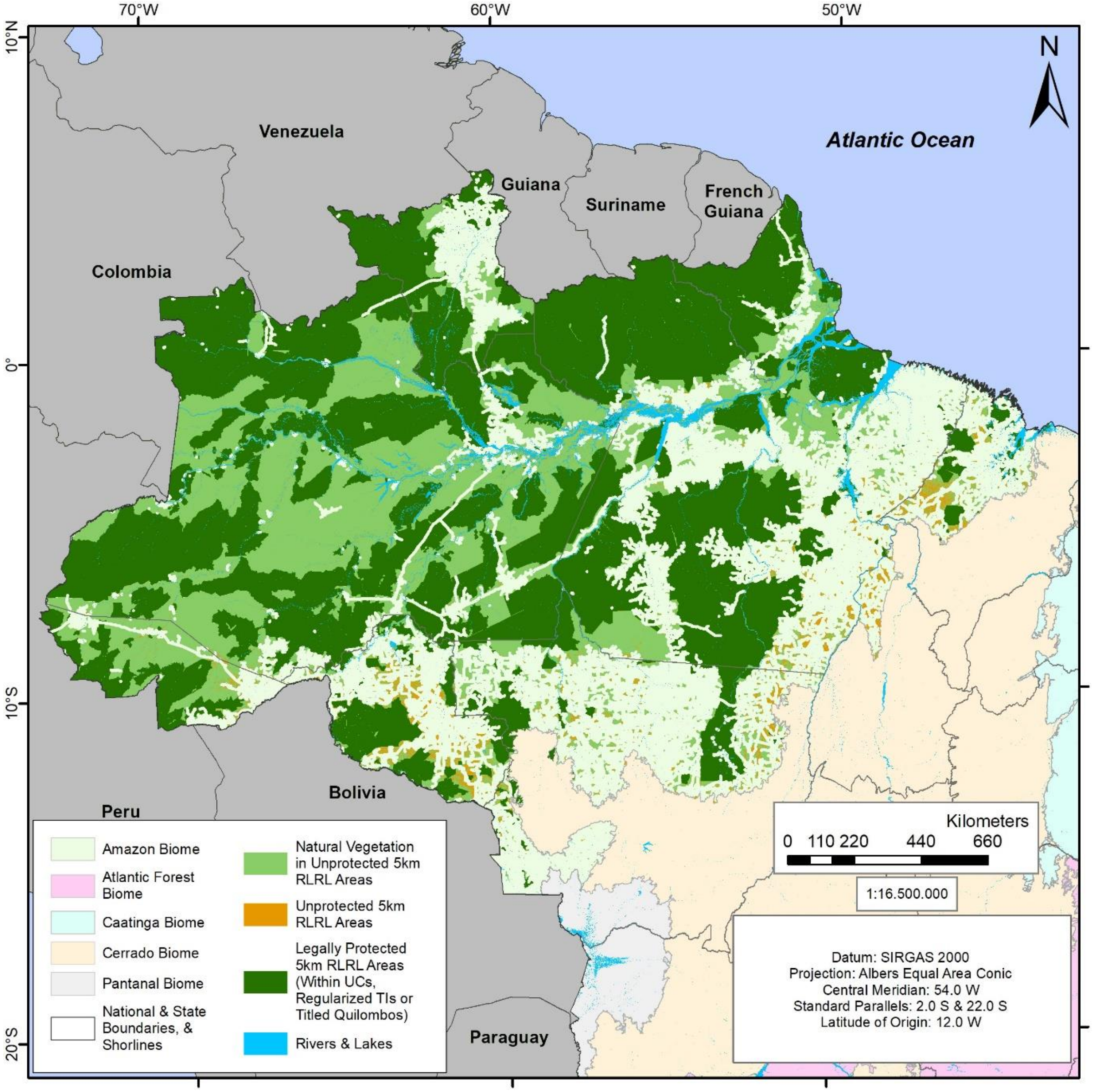


Amazon Biome's 5km RLRL Areas, Legally Protected Areas (LPAs), and Native Vegetation Coverage

**Fig. S20.**

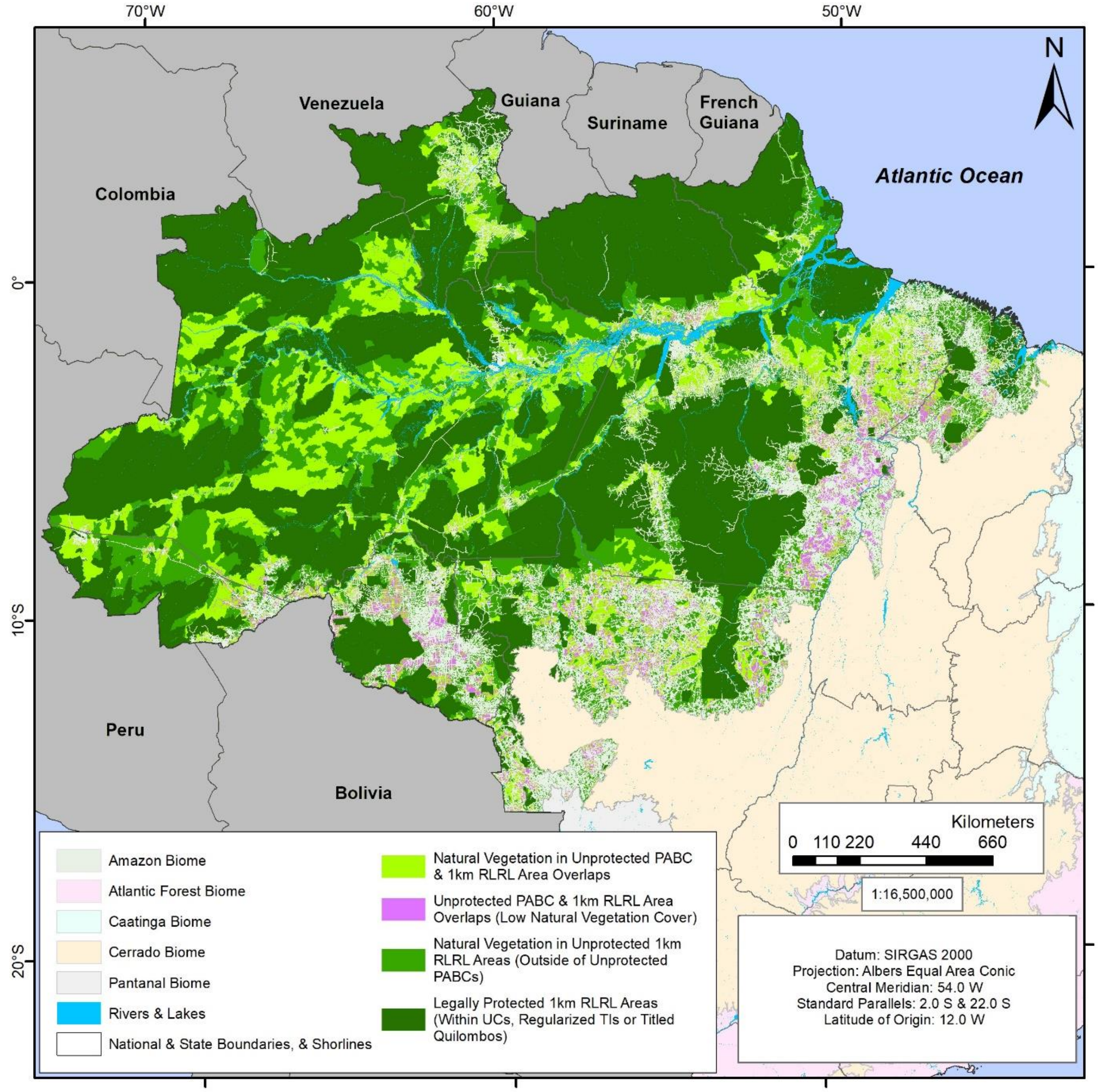


Amazon Biome's 1km RLRL Areas, Legally Protected Areas (LPAs), Unprotected Priority Areas for Biodiversity Conservation (PABCs) and Native Vegetation Coverage

**Fig. S21.**

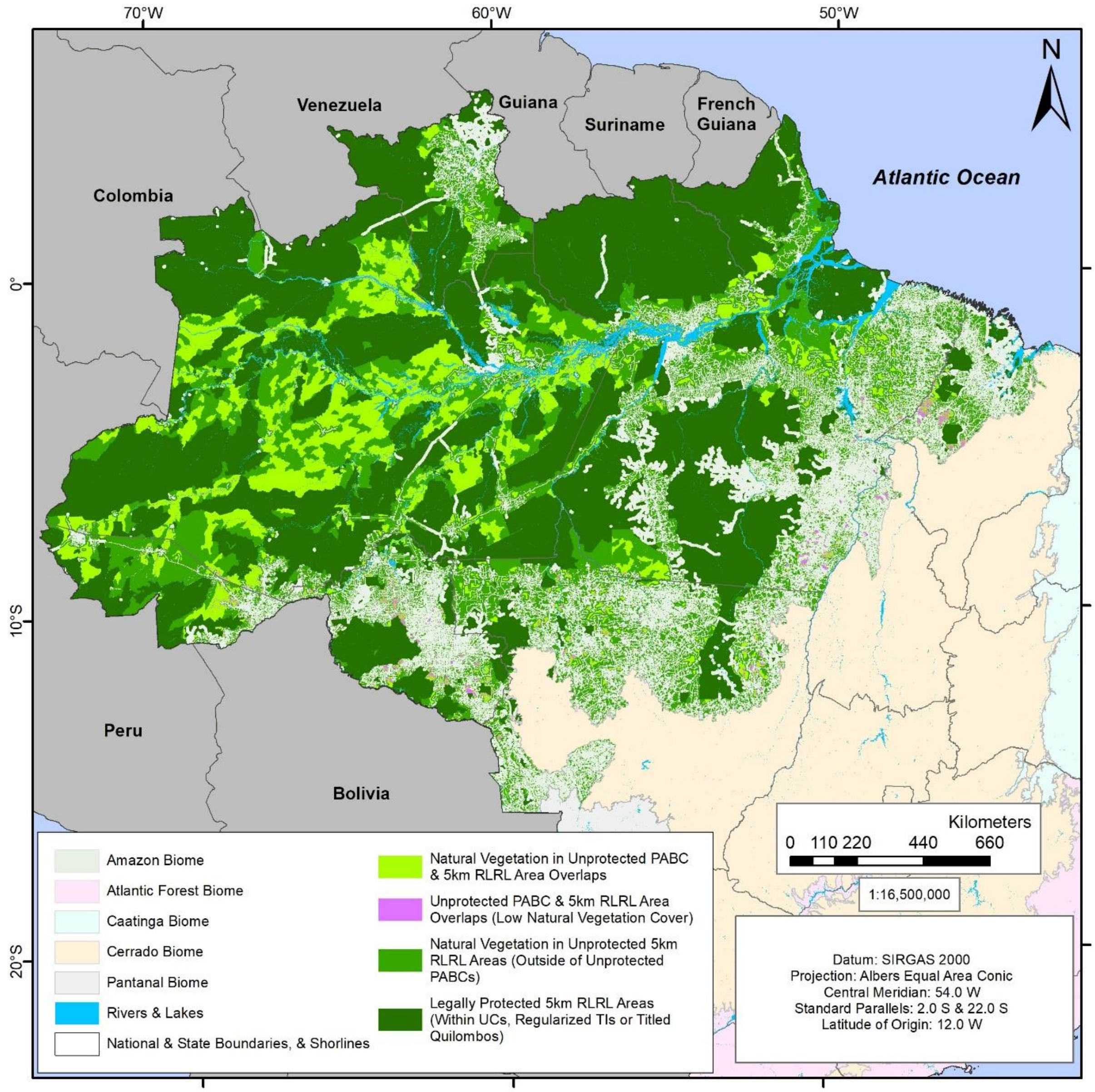


Amazon Biome's 5km RLRL Areas, Legally Protected Areas (LPAs), Unprotected Priority Areas for Biodiversity Conservation (PABCs) and Native Vegetation Coverage

**Fig. S22.**

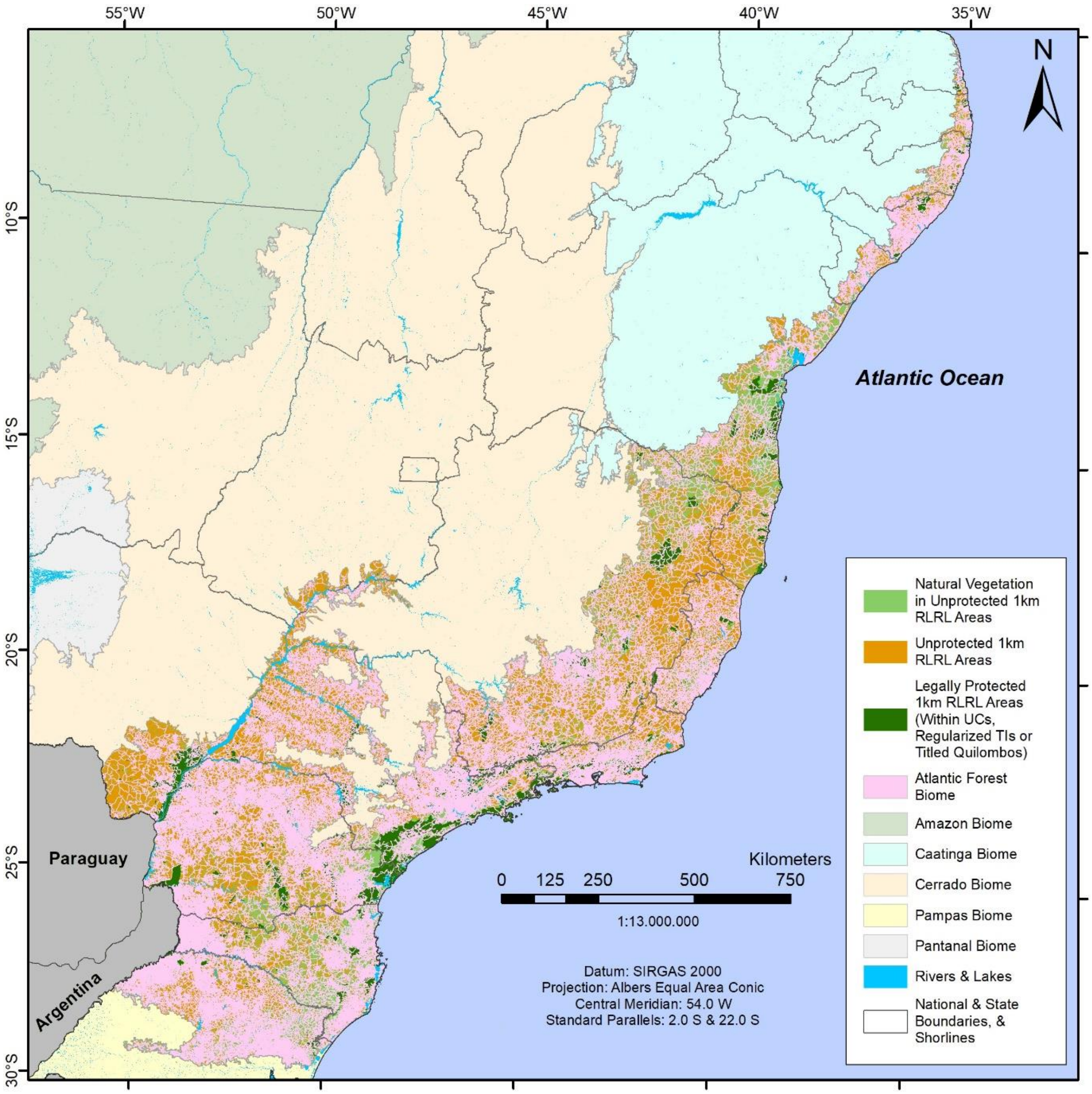


Atlantic Forest Biome's 1km RLRL Areas, LPAs, and Native Vegetation Coverage

**Fig. S23.**

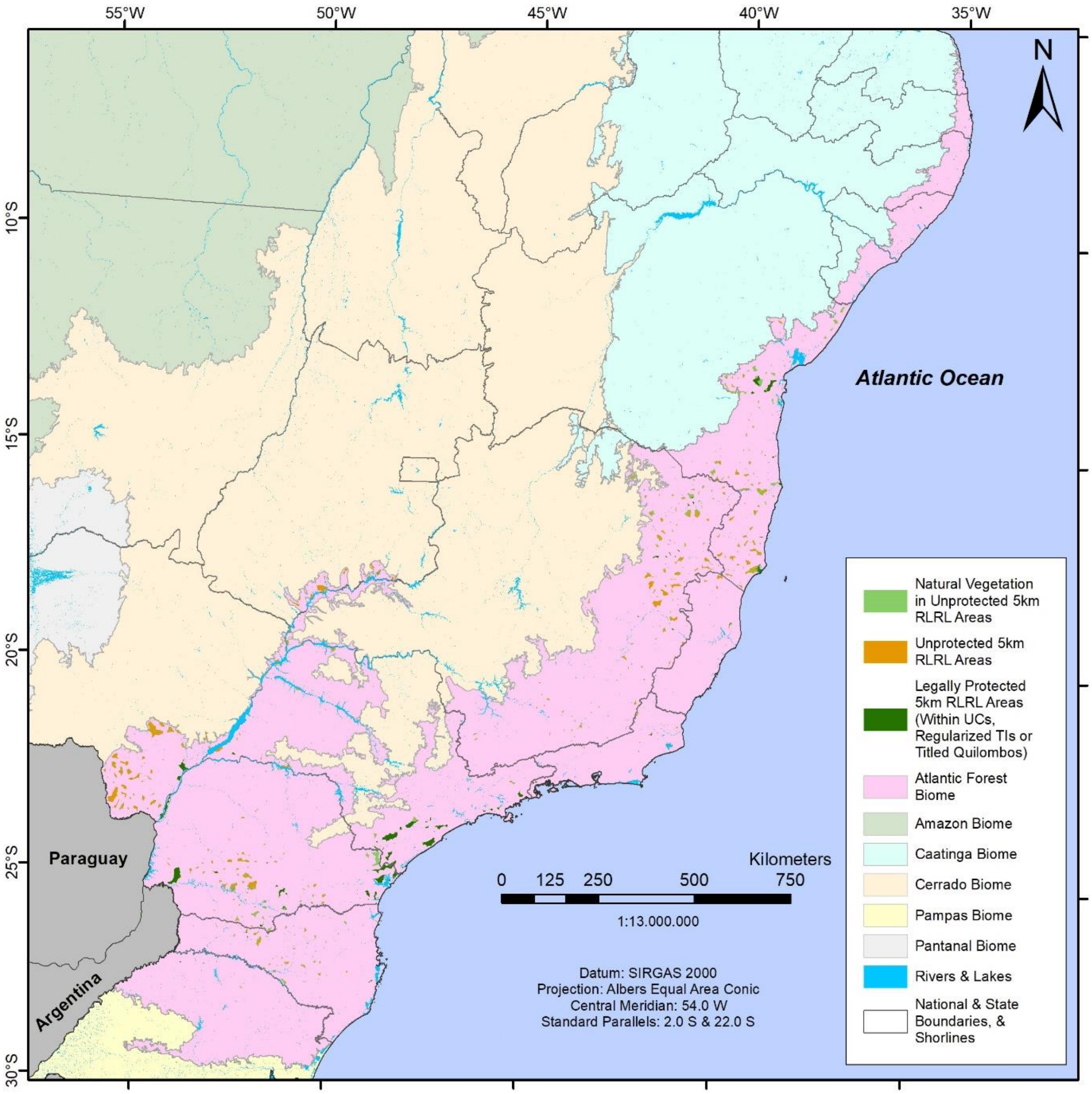


Atlantic Forest Biome's 5km RLRL Areas, LPAs, and Native Vegetation Coverage

**Fig. S24.**

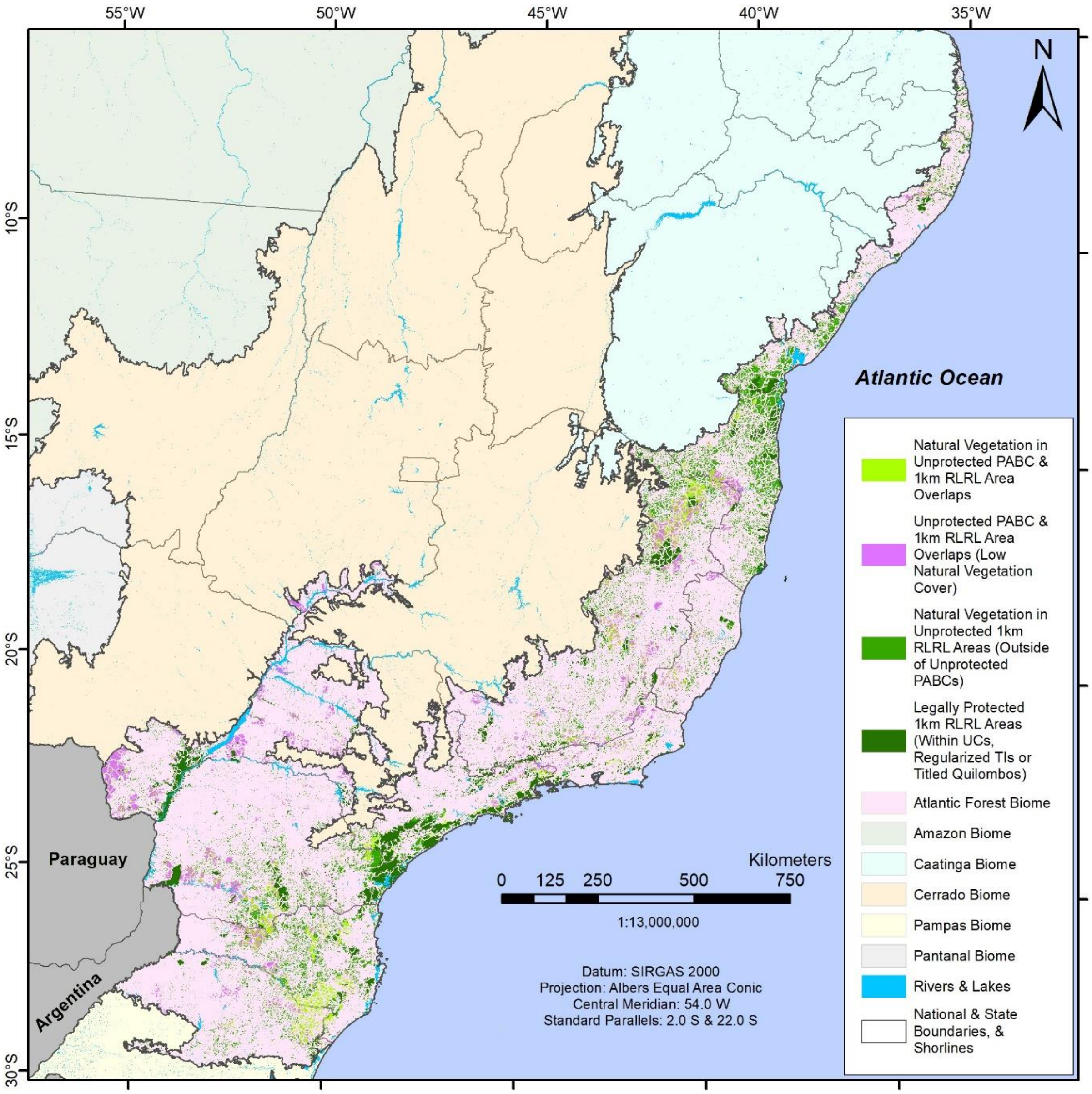


Atlantic Forest Biome's 1km RLRL Areas, LPAs, Unprotected Priority Areas for Biodiversity Conservation (PABCs) and Native Vegetation Coverage

**Fig. S25.**

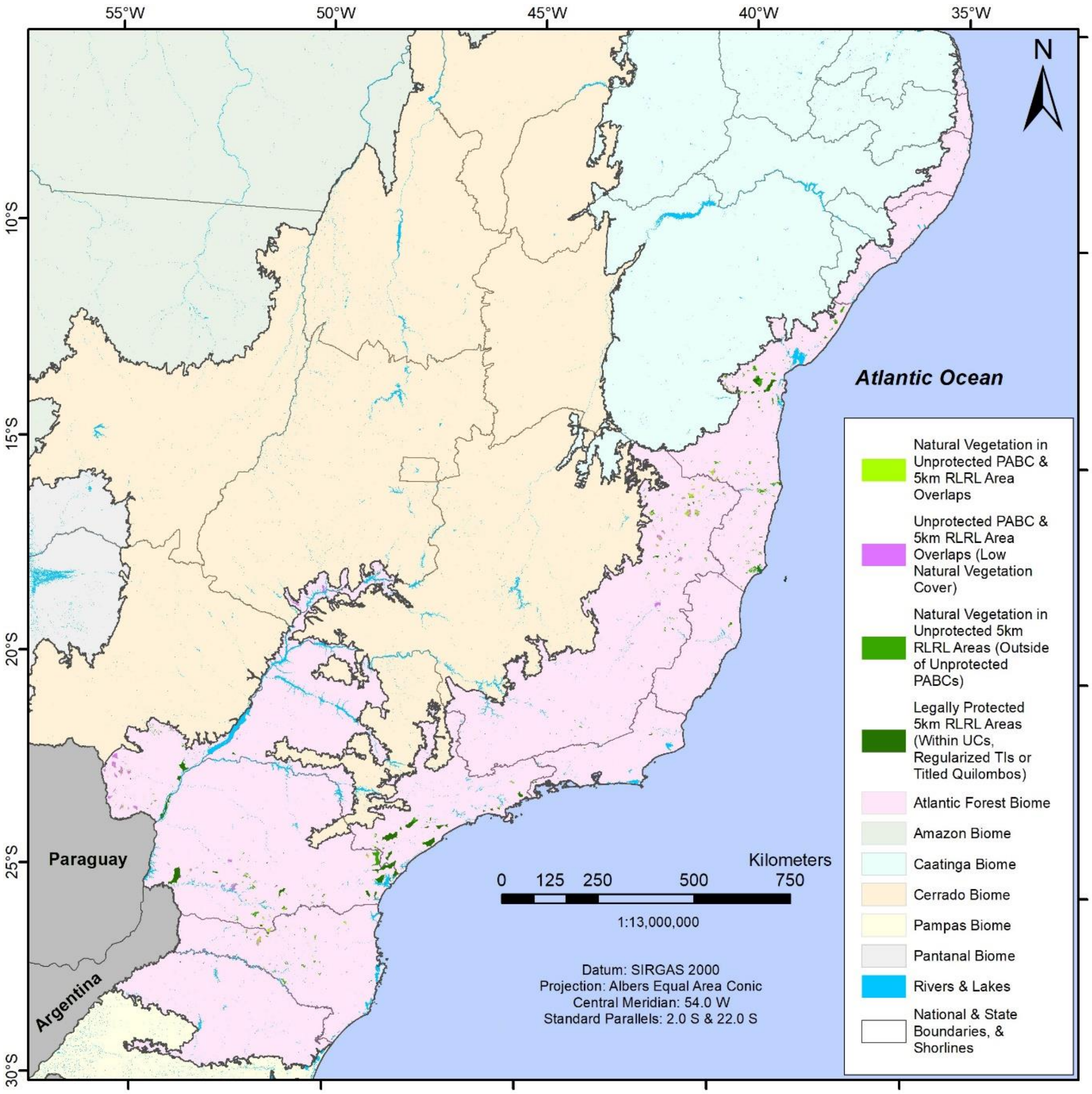


Atlantic Forest Biome's 5km RLRL Areas, Legally Protected Areas (LPAs), Unprotected Priority Areas for Biodiversity Conservation (PABCs) and Native Vegetation Coverage

**Fig. S26.**

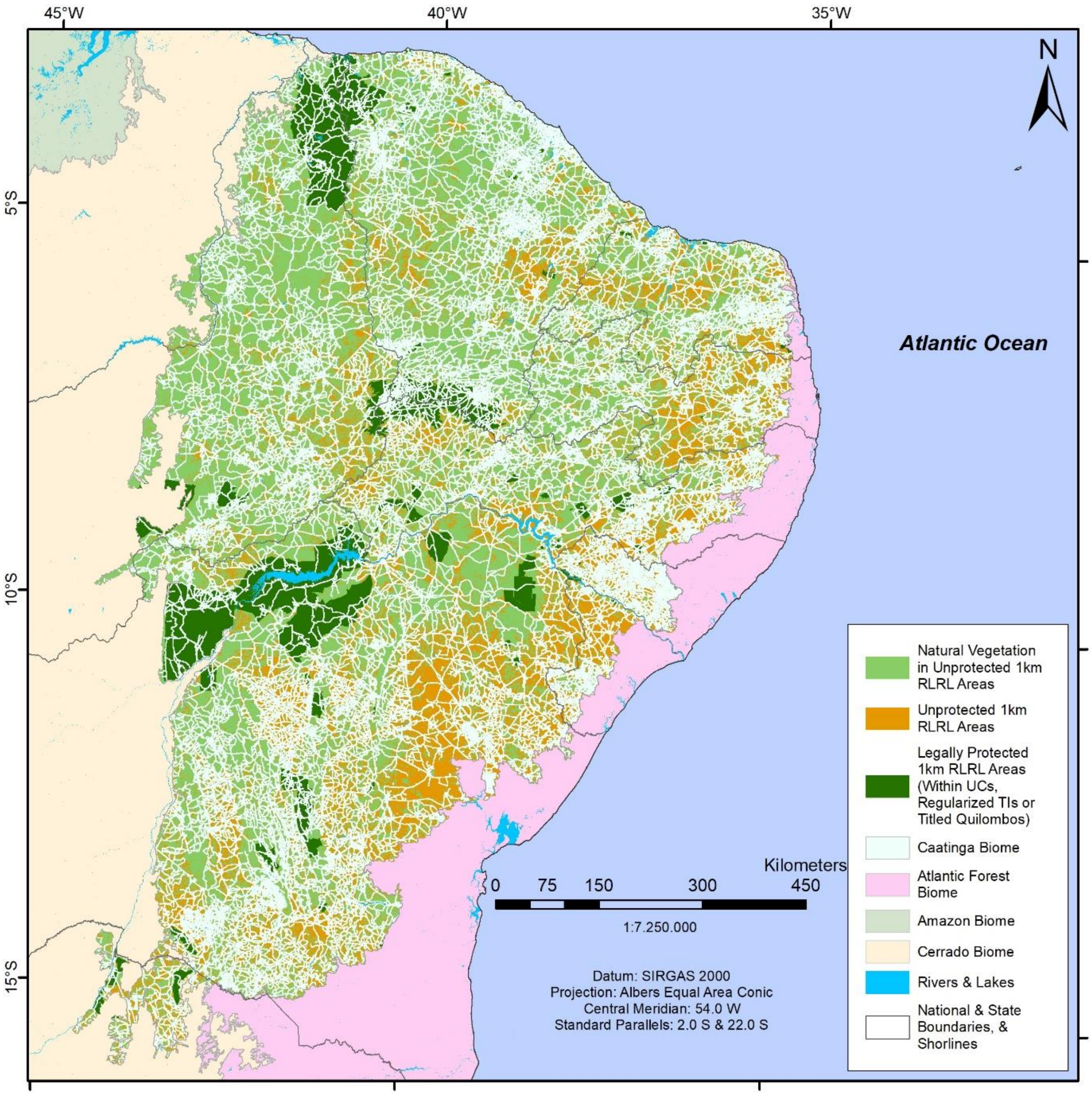


Caatinga Biome's 1km RLRL Areas, Legally Protected Areas (LPAs), and Native Vegetation Coverage

**Fig. S27.**

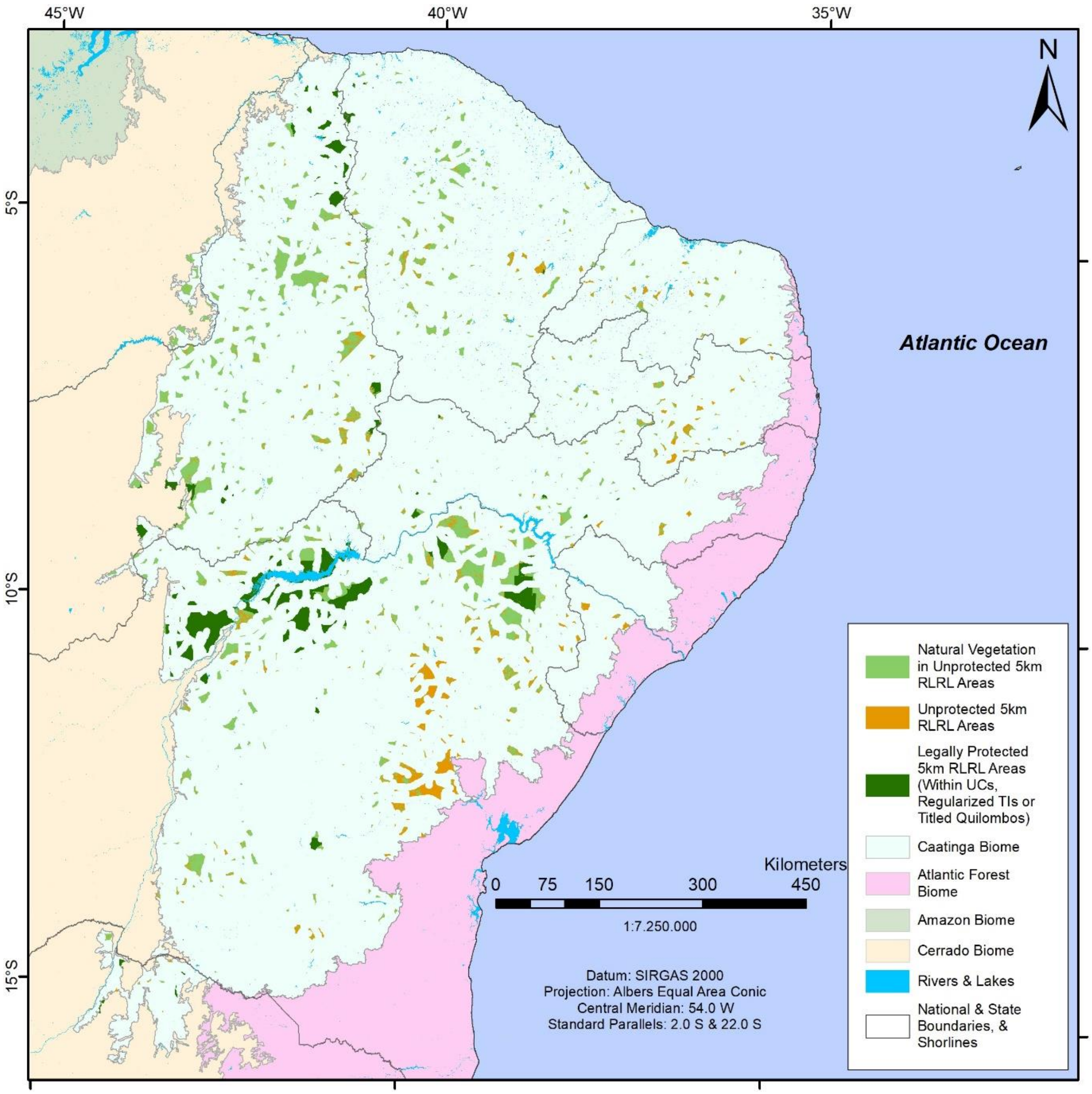


Caatinga Biome's 5km RLRL Areas, Legally Protected Areas (LPAs), and Native Vegetation Coverage

**Fig. S28.**

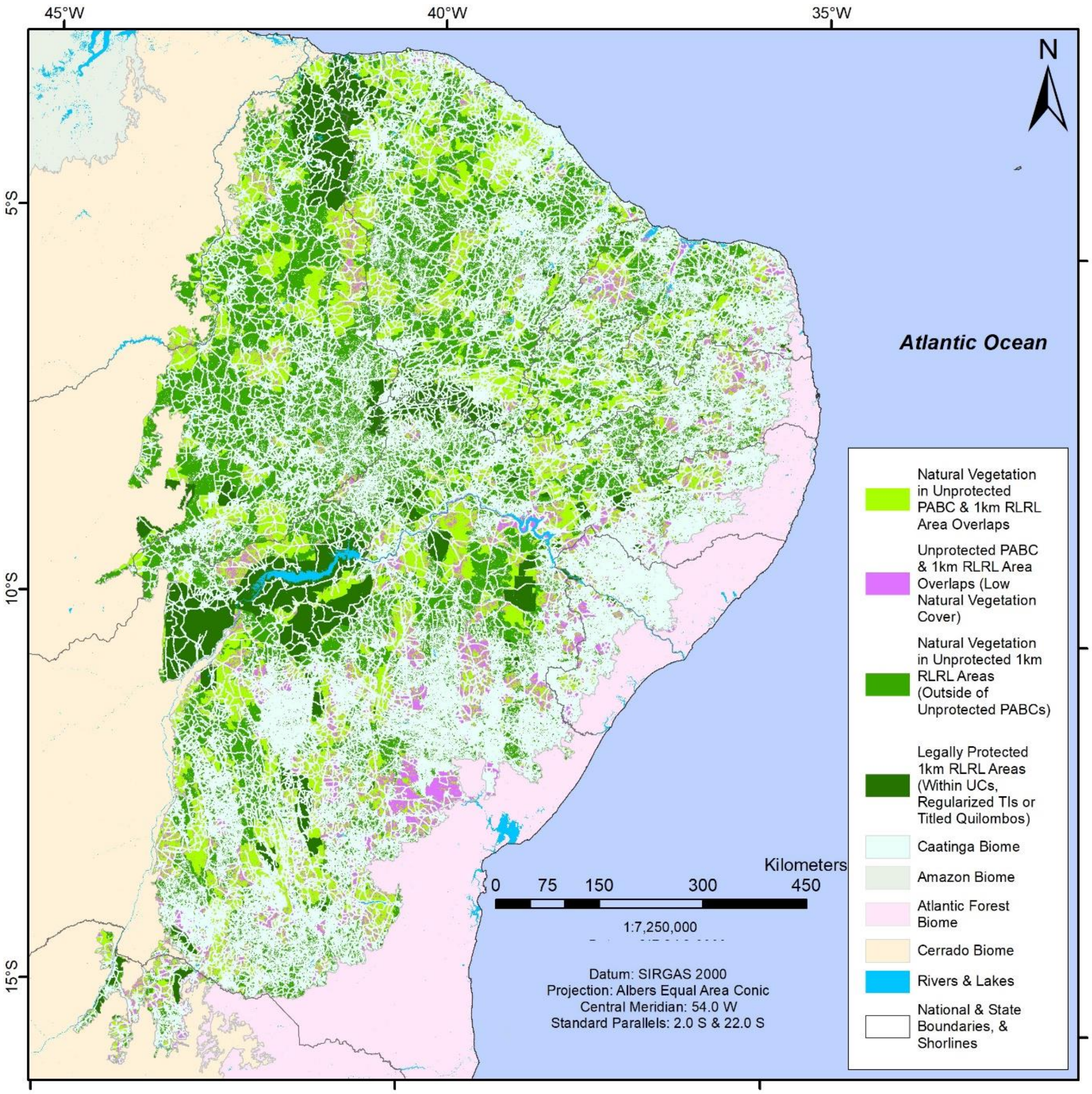


Caatinga Biome's 1km RLRL Areas, Legally Protected Areas (LPAs), Unprotected Priority Areas for Biodiversity Conservation (PABCs) and Native Vegetation Coverage

**Fig. S29.**

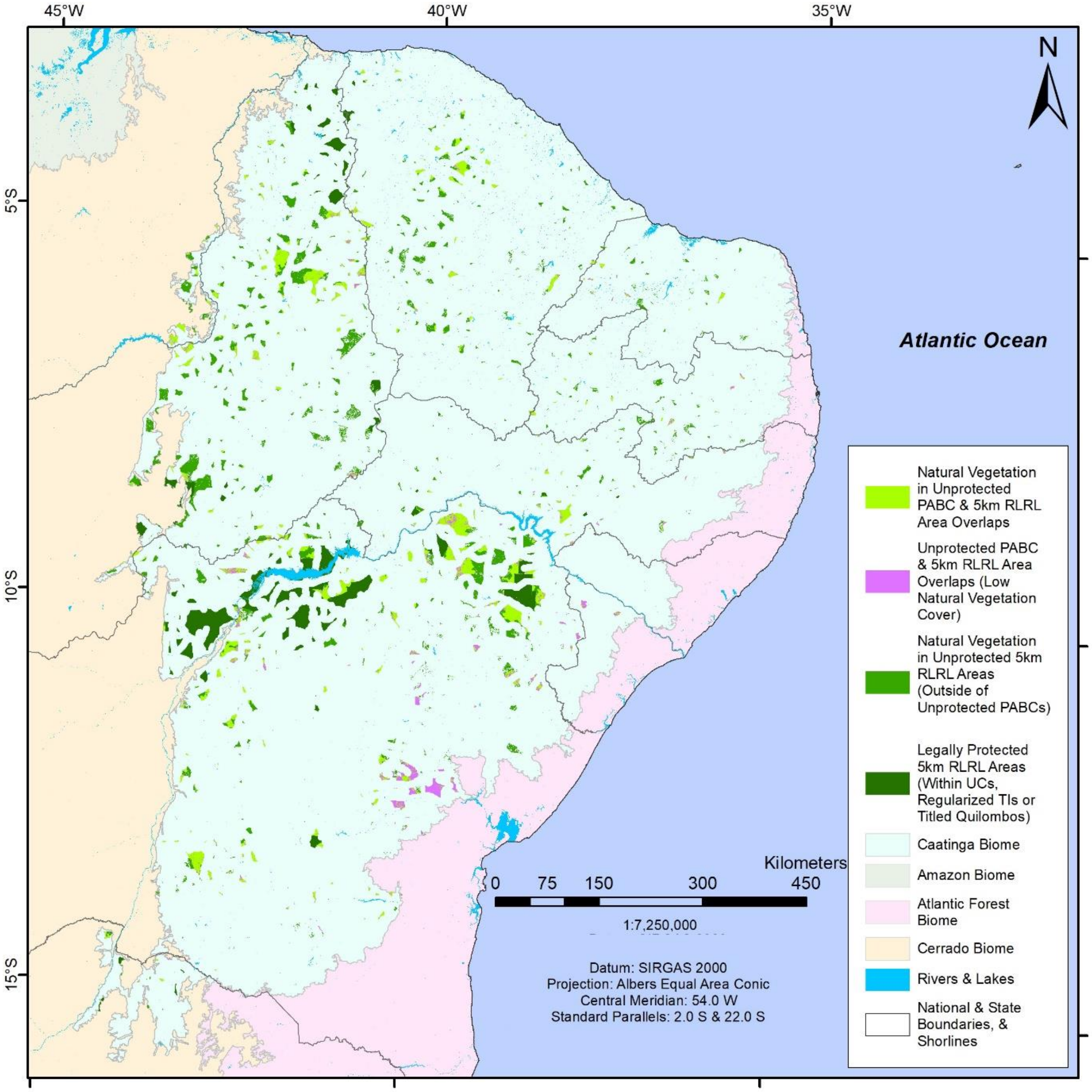


Caatinga Biome's 5km RLRL Areas, Legally Protected Areas (LPAs), Unprotected Priority Areas for Biodiversity Conservation (PABCs) and Native Vegetation Coverage

**Fig. S30.**

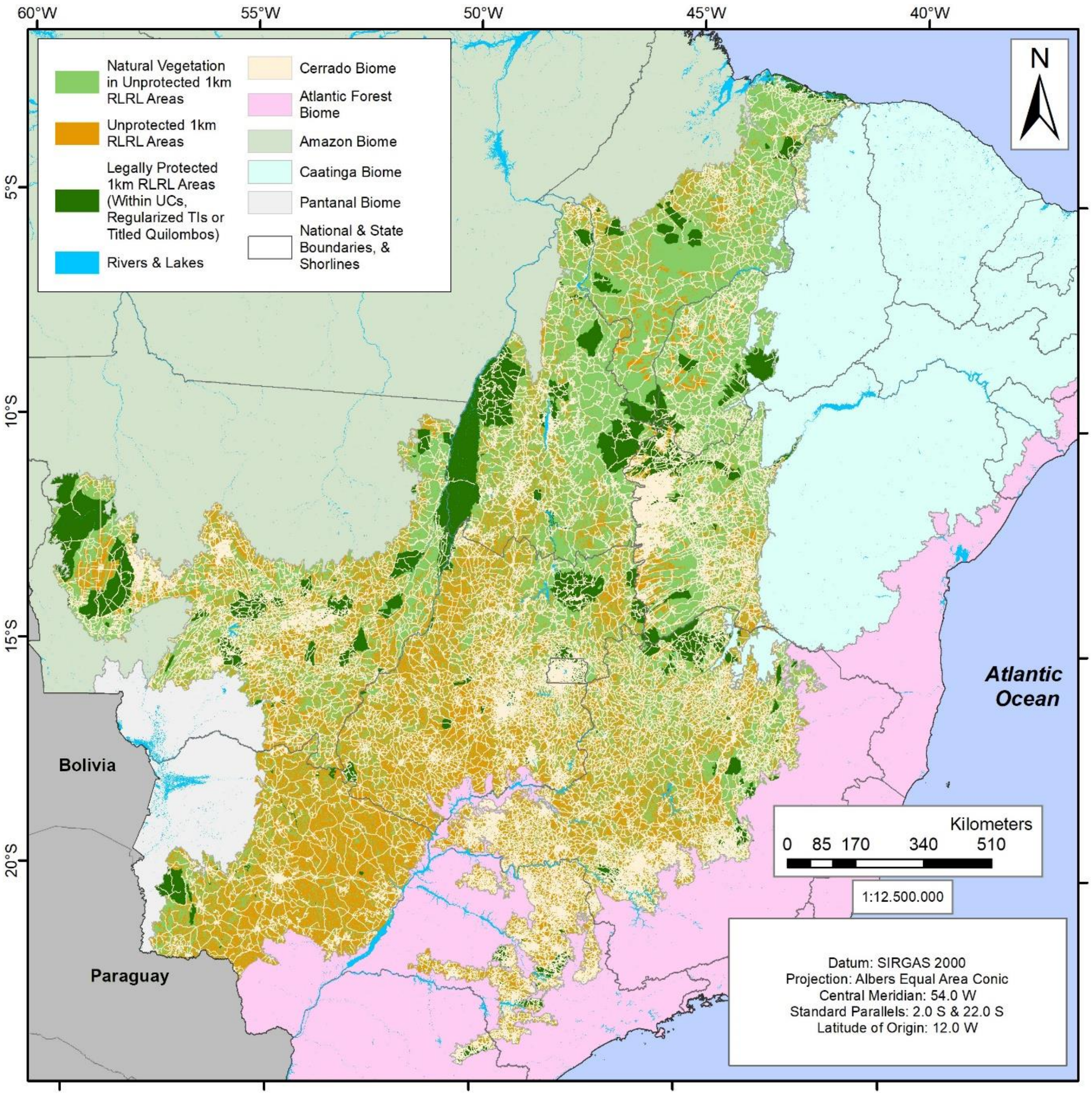


Cerrado Biome's 1km RLRL Areas, Legally Protected Areas (LPAs), and Native Vegetation Coverage

**Fig. S31.**

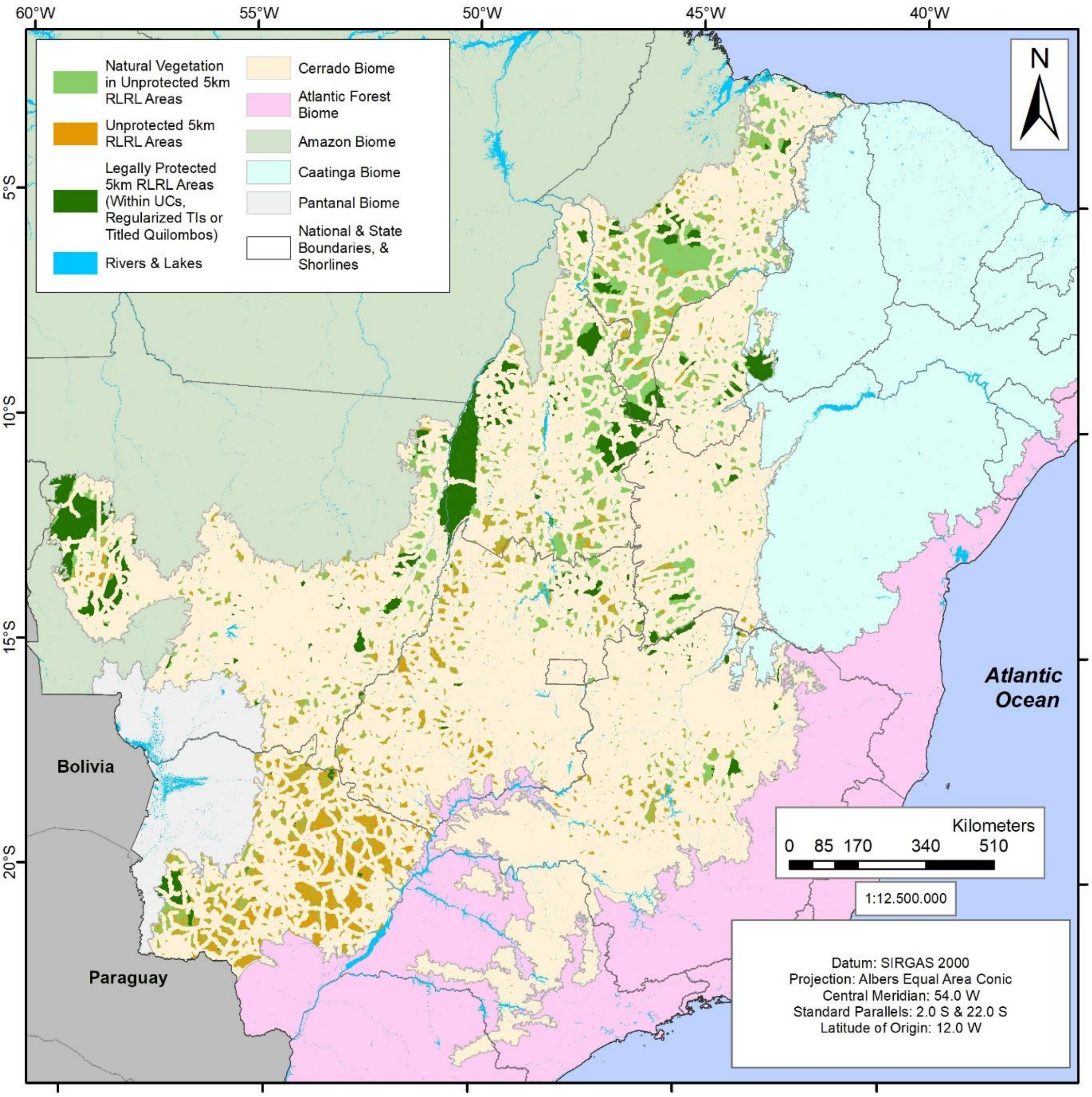


Cerrado Biome's 5km RLRL Areas, Legally Protected Areas (LPAs), and Native Vegetation Coverage

**Fig. S32.**

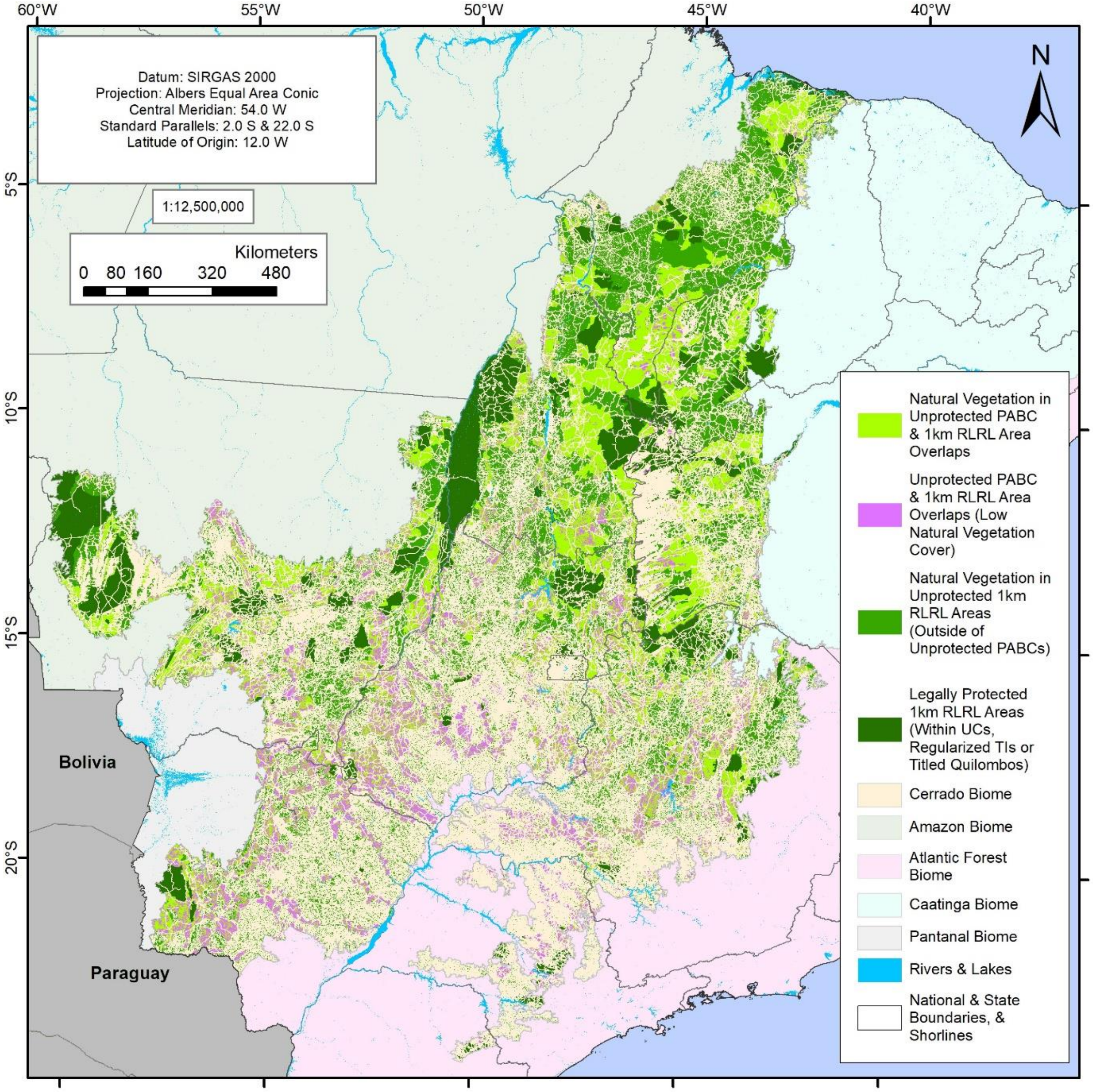


Cerrado Biome's 1km RLRL Areas, Legally Protected Areas (LPAs), Unprotected Priority Areas for Biodiversity Conservation (PABCs) and Native Vegetation Coverage

**Fig. S33.**

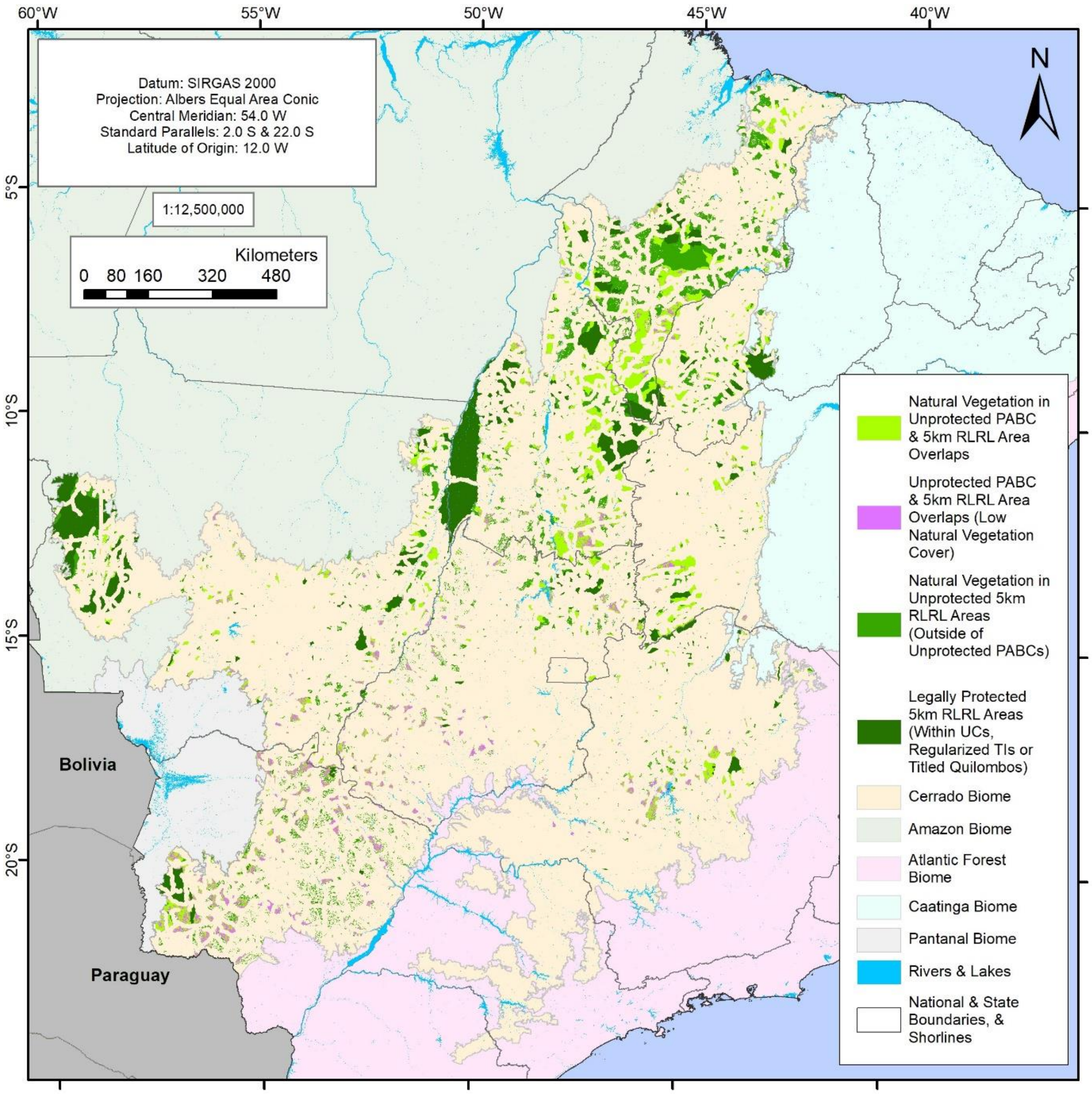


Cerrado Biome's 5km RLRL Areas, Legally Protected Areas (LPAs), Unprotected Priority Areas for Biodiversity Conservation (PABCs) and Native Vegetation Coverage

**Fig. S34.**

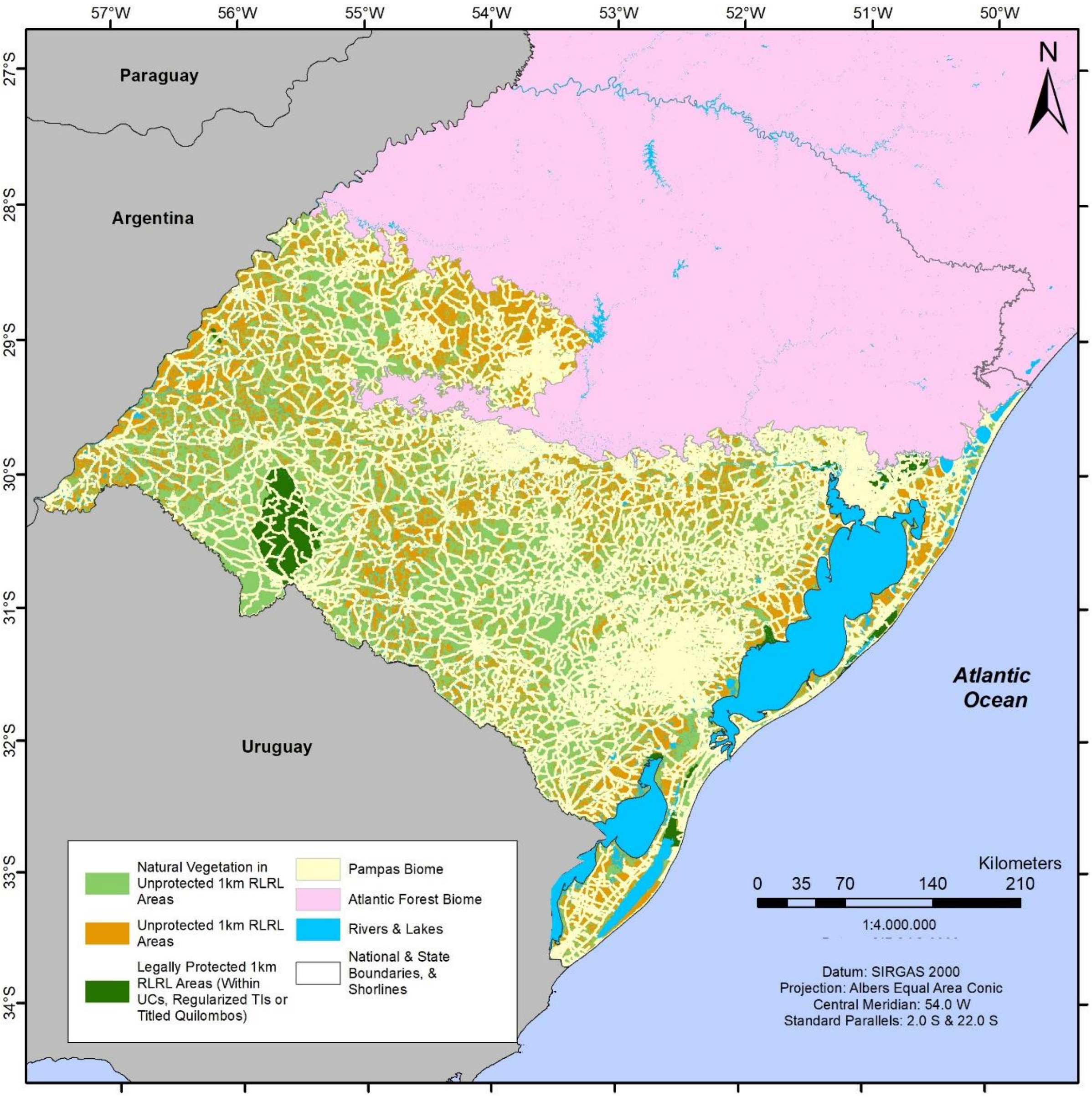


Pampas Biome's 1km RLRL Areas, Legally Protected Areas (LPAs), and Native Vegetation Coverage

**Fig. S35.**

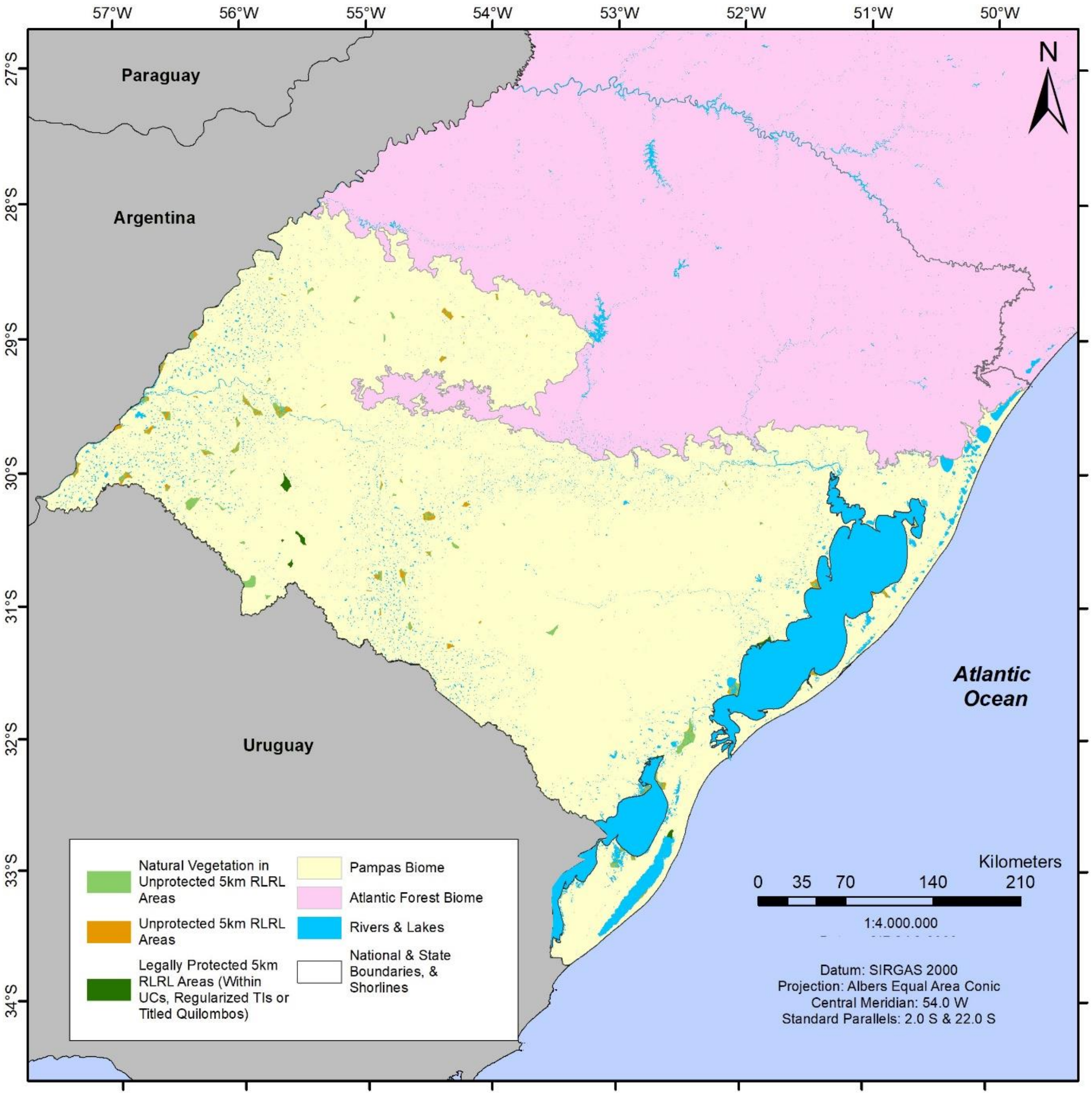


Pampas Biome's 5km RLRL Areas, Legally Protected Areas (LPAs), and Native Vegetation Coverage

**Fig. S36.**

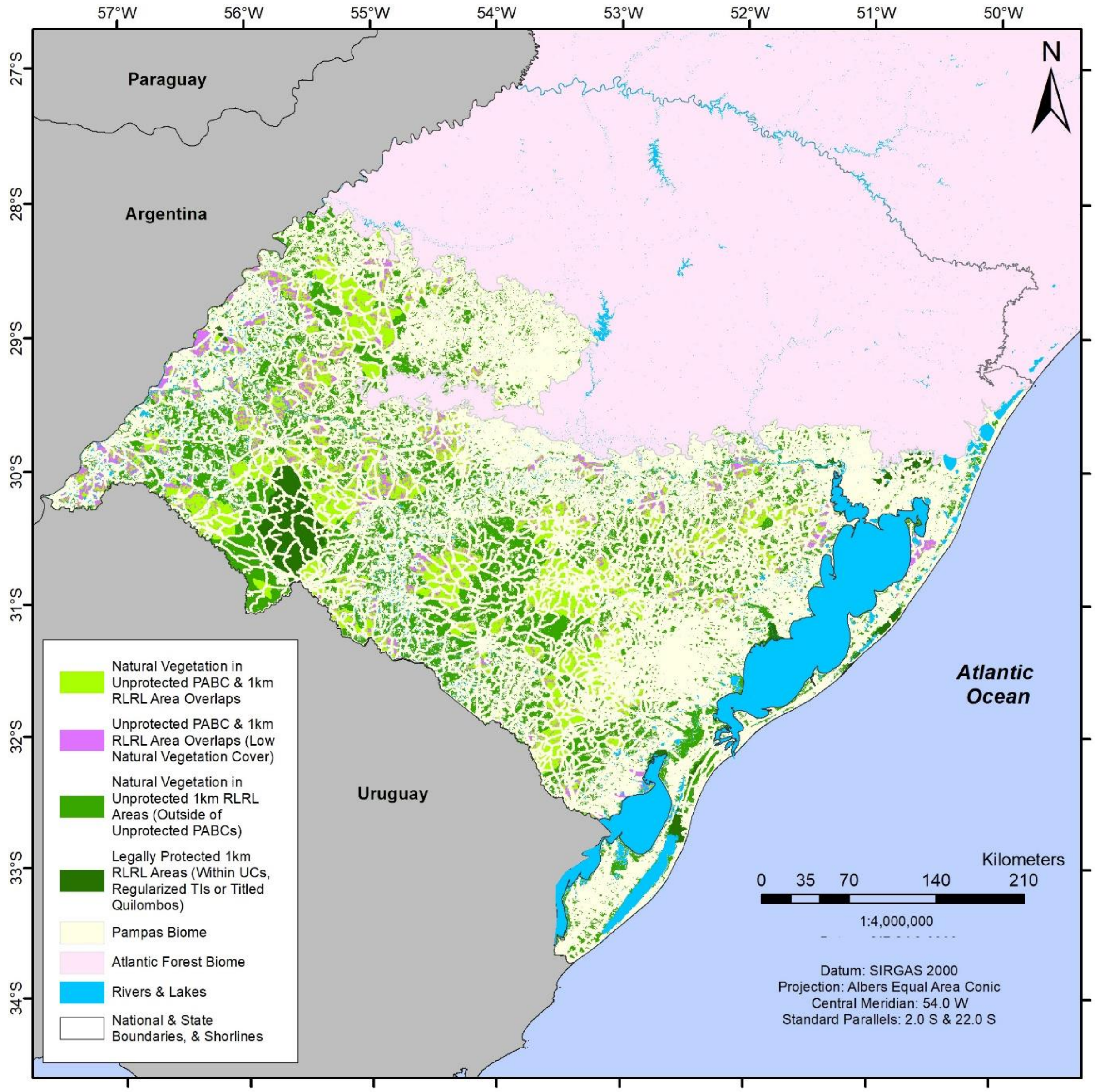


Pampas Biome's 1km RLRL Areas, Legally Protected Areas (LPAs), Unprotected Priority Areas for Biodiversity Conservation (PABCs) and Native Vegetation Coverage

**Fig. S37.**

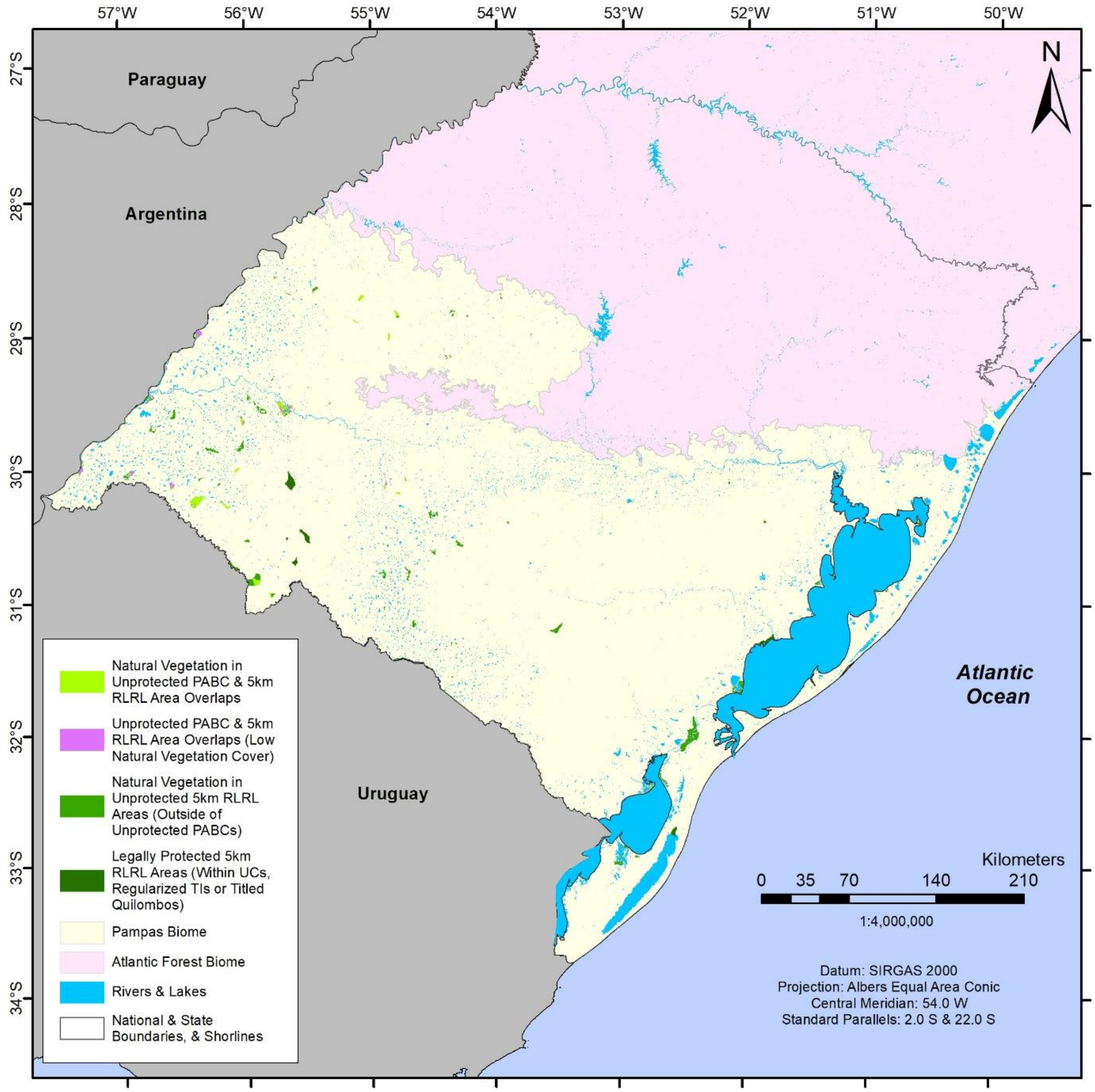


Pampas Biome's 5km RLRL Areas, Legally Protected Areas (LPAs), Unprotected Priority Areas for Biodiversity Conservation (PABCs) and Native Vegetation Coverage

**Fig. S38.**

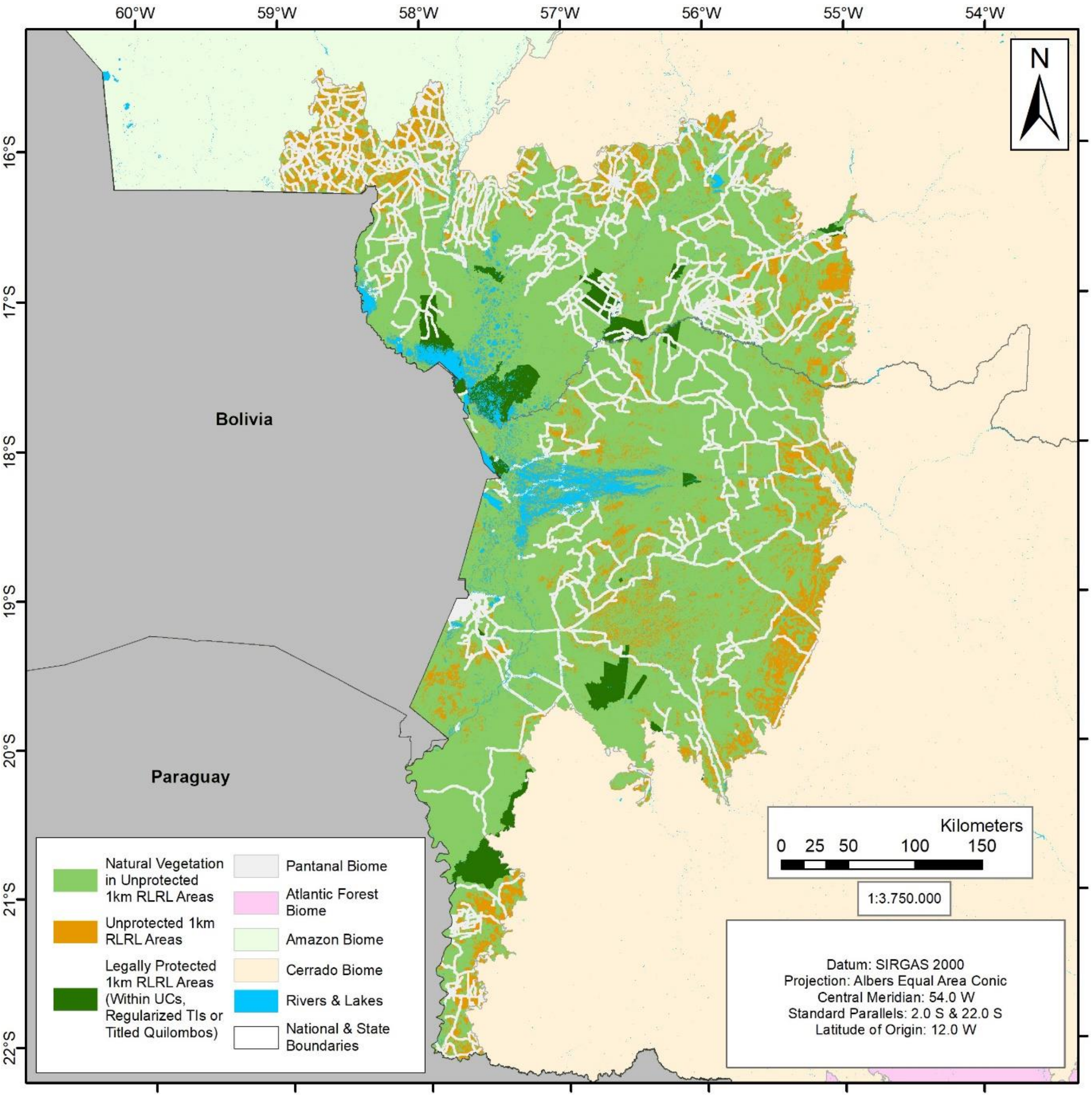


Pantanal Biome's 1km RLRL Areas, Legally Protected Areas (LPAs), and Native Vegetation Coverage

**Fig. S39.**

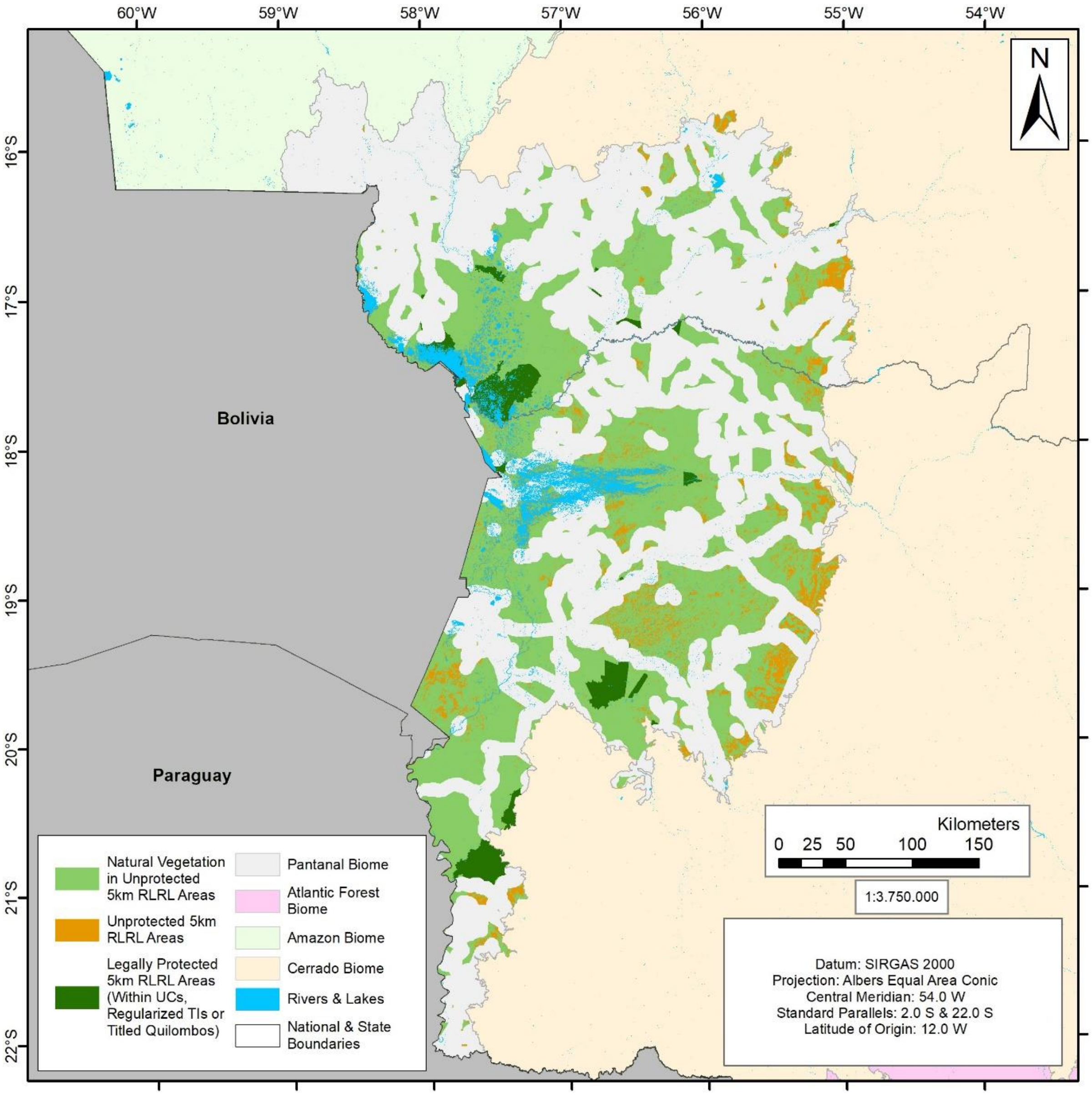


Pantanal Biome's 5km RLRL Areas, Legally Protected Areas (LPAs), and Native Vegetation Coverage

**Fig. S40.**

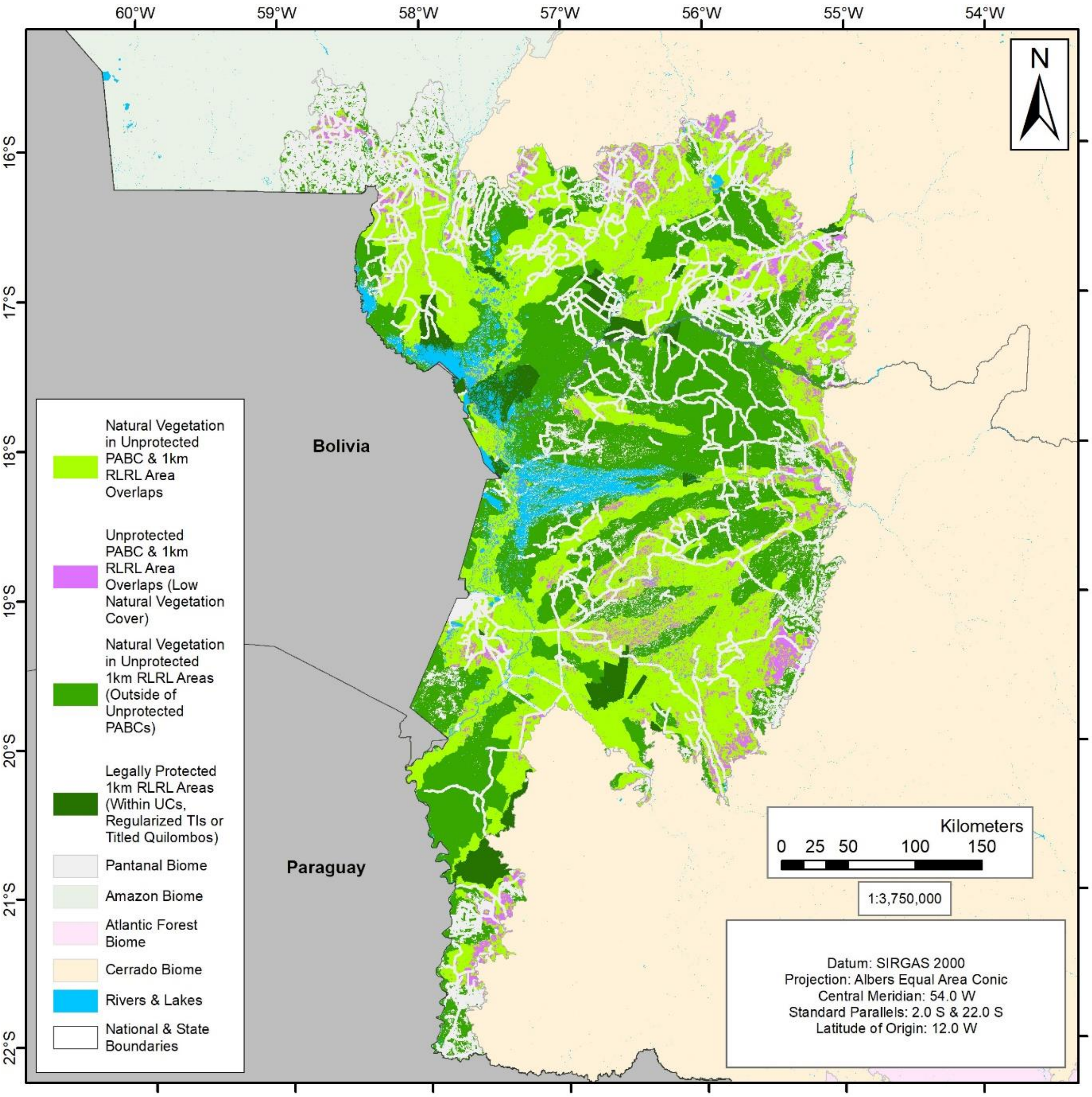


Pantanal Biome's 1km RLRL Areas, Legally Protected Areas (LPAs), Unprotected Priority Areas for Biodiversity Conservation (PABCs) and Native Vegetation Coverage

**Fig. S41.**

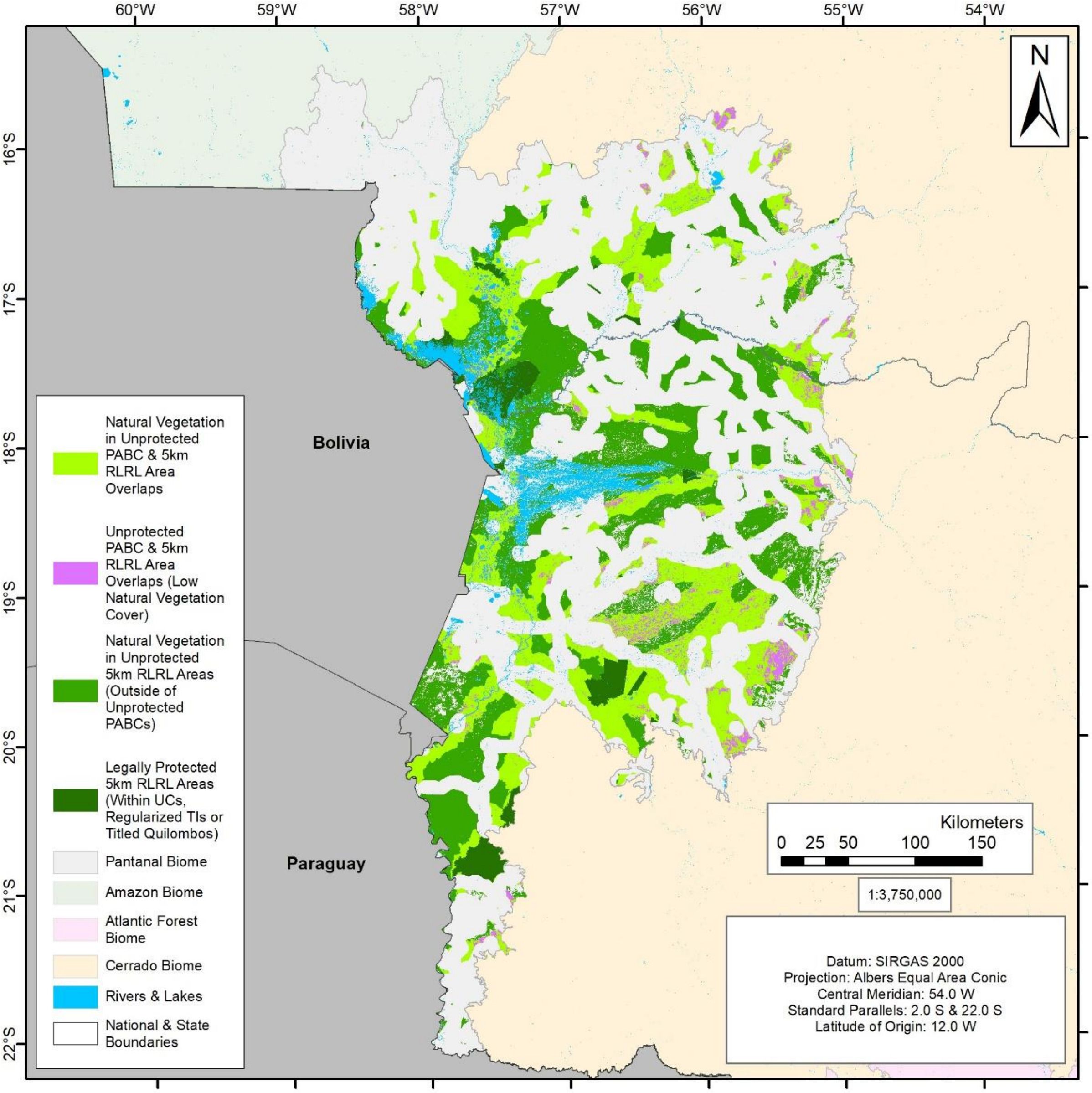


Pantanal Biome's 5km RLRL Areas, Legally Protected Areas (LPAs), Unprotected Priority Areas for Biodiversity Conservation (PABCs) and Native Vegetation Coverage

**Fig. S42.**

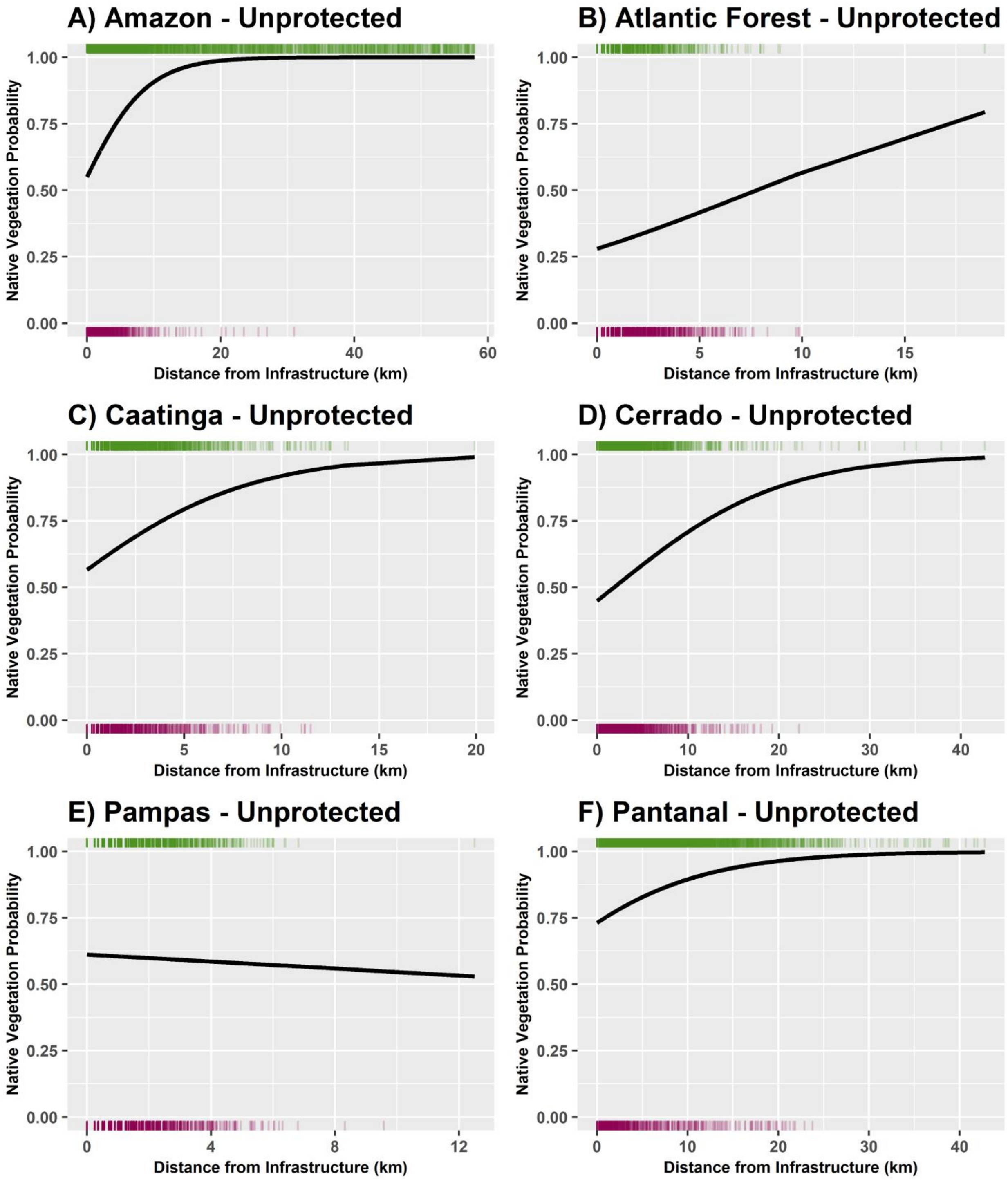


**Unprotected Areas Stratified Logistic Regression Curve: Probability (black line) of encountering native vegetation (green) in Unprotected portions of biomes as distance increases from infrastructure.** For all biomes (**A, B, C**, **D, F**), except for the Pampas (**E**), pixels in unprotected areas that are farther away from infrastructure have a higher probability of being covered by native vegetation.

**Fig. S43.**

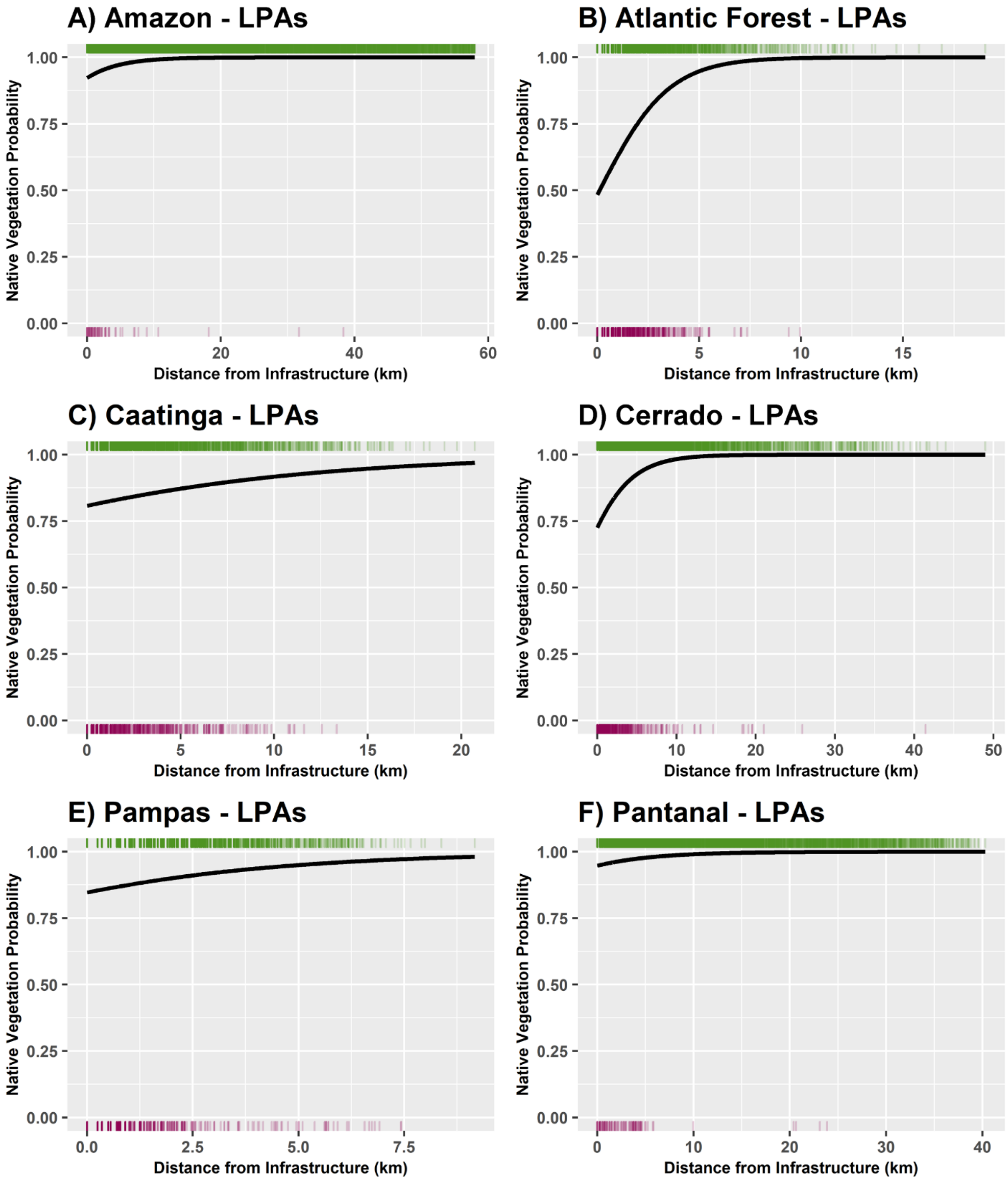


**LPA (Protected Areas) Stratified Logistic Regression Curve: Probability (black line) of encountering native vegetation (green) in LPA portions of biomes as distance increases from infrastructure.** For all biomes (**A, B, C**, **D**, **E**), except for the Pantanal (**F**) pixels in unprotected areas that are farther away from infrastructure have a higher probability of being covered by native vegetation. Purple represents anthropic land use.

**Fig. S44.**

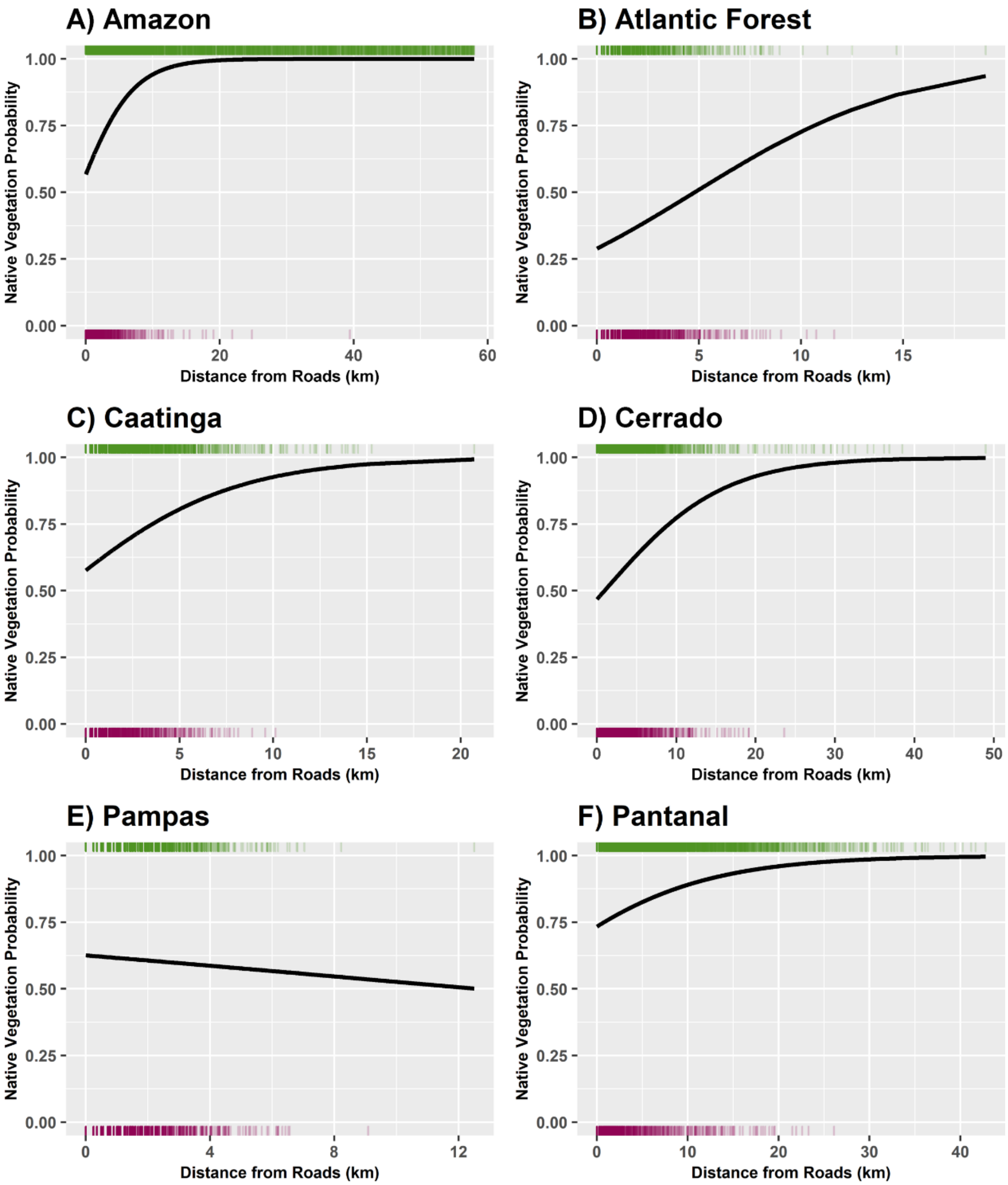


**Roads Stratified Logistic Regression Curve: Probability (black line) of encountering native vegetation (green) as distance increases from roads.** For all biomes (**A, B, C**, **D**, **F**), except the Pampas (**E**), pixels that are farther away from roads have a higher probability of being covered by native vegetation. Purple represents anthropic land use.

**Fig. S45.**

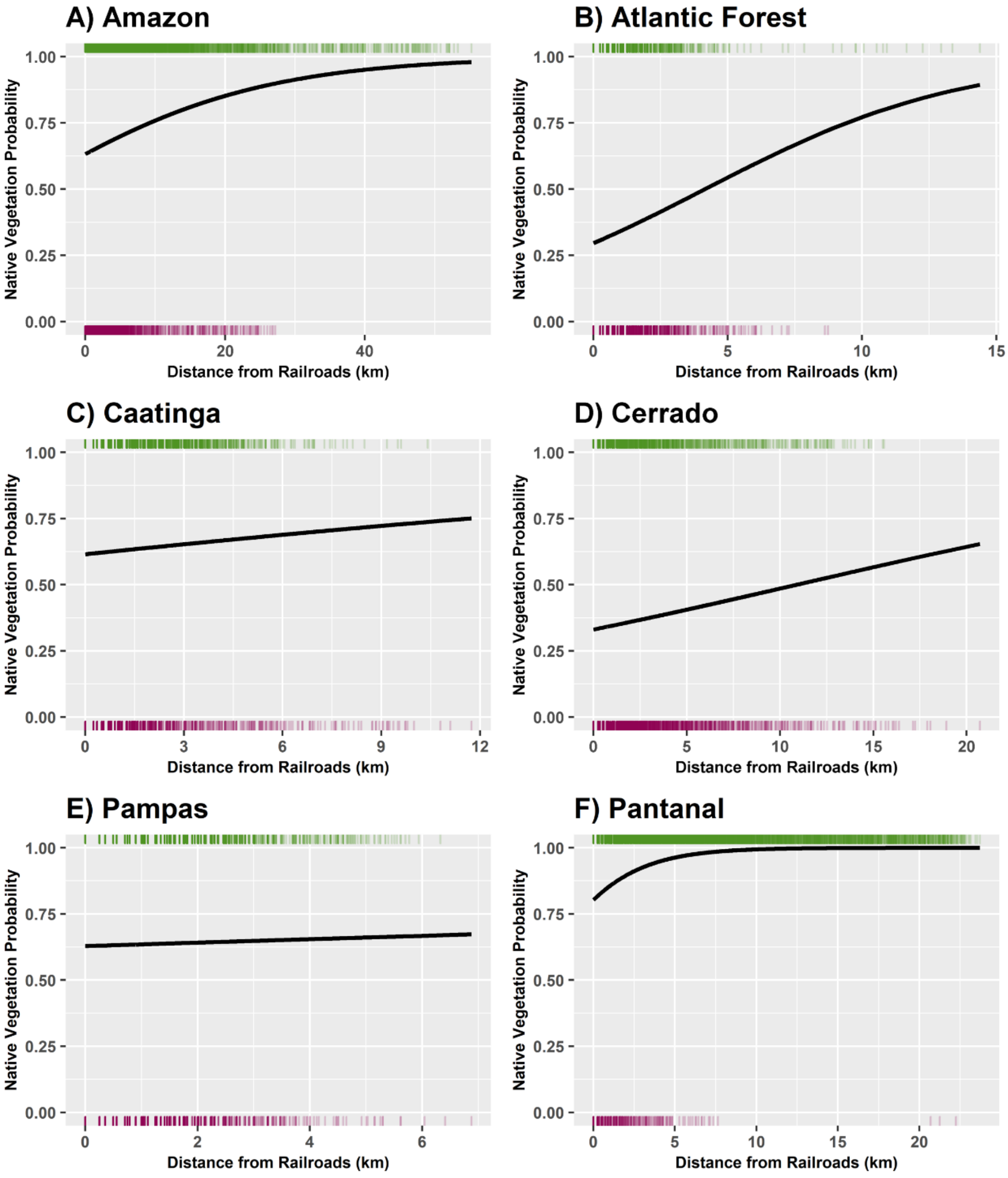


**Railroads Stratified Logistic Regression Curve: Probability (black line) of encountering native vegetation (green) as distance increases from railroads.** For all biomes (**A, B, C**, **D**, **E, F**) pixels that are farther away from railroads have a higher probability of being covered by native vegetation. Purple represents anthropic land use.

**Fig. S46.**

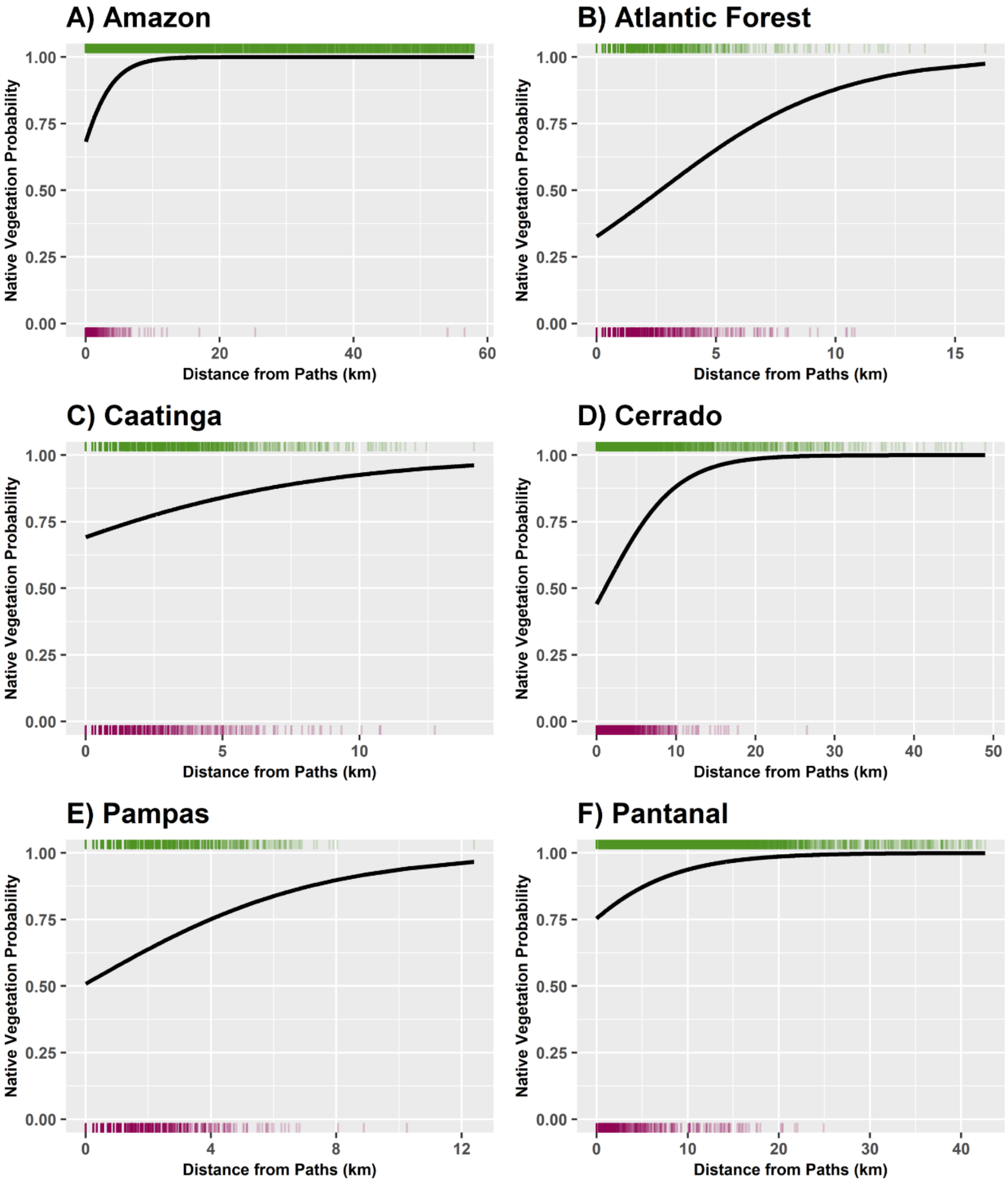


**Pathways Stratified Logistic Regression Curve: Probability (black line) of encountering native vegetation (green) as distance increases from pathways.** For all biomes (**A, B, C**, **D**, **E**, **F**), pixels that are farther away from pathways have a higher probability of being covered by native vegetation. Purple represents anthropic land use.

**Fig. S47.**

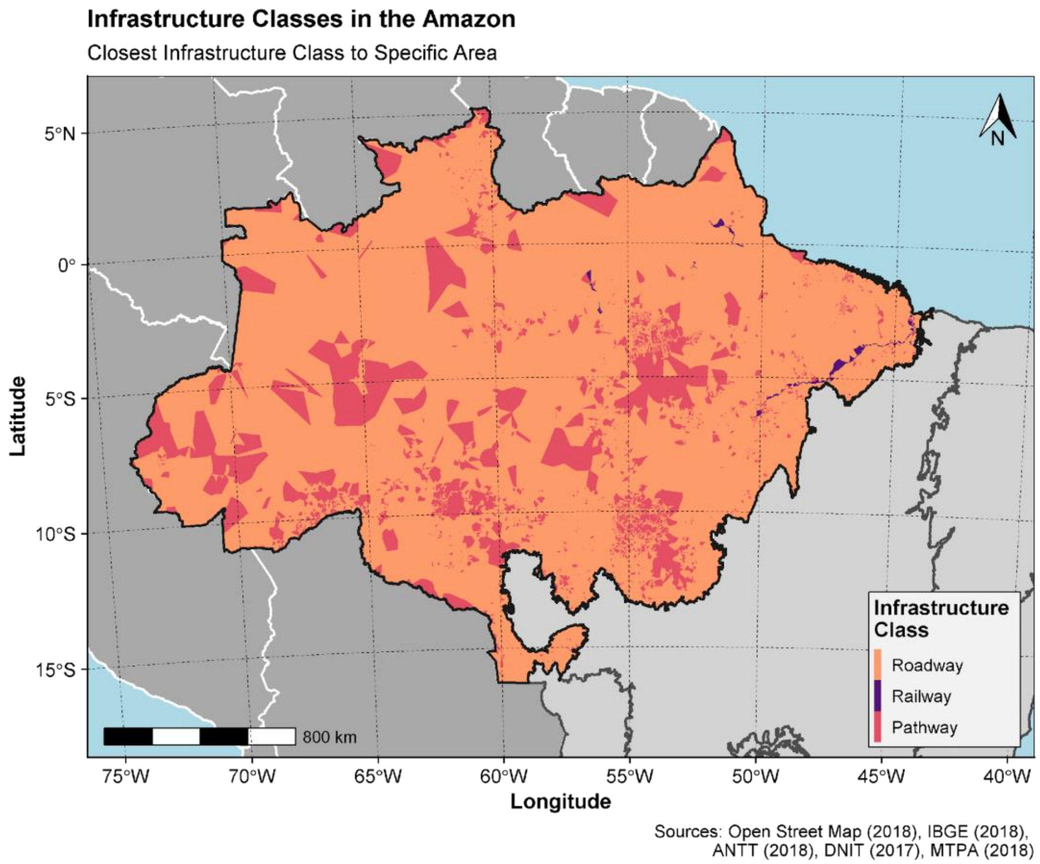


Amazon areas dominated by specific infrastructure types (for the simplified decision tree, see how variable *RLRL_Infra* was determined in R scripts **3A** in **Data S3**).

**Fig. S48.**

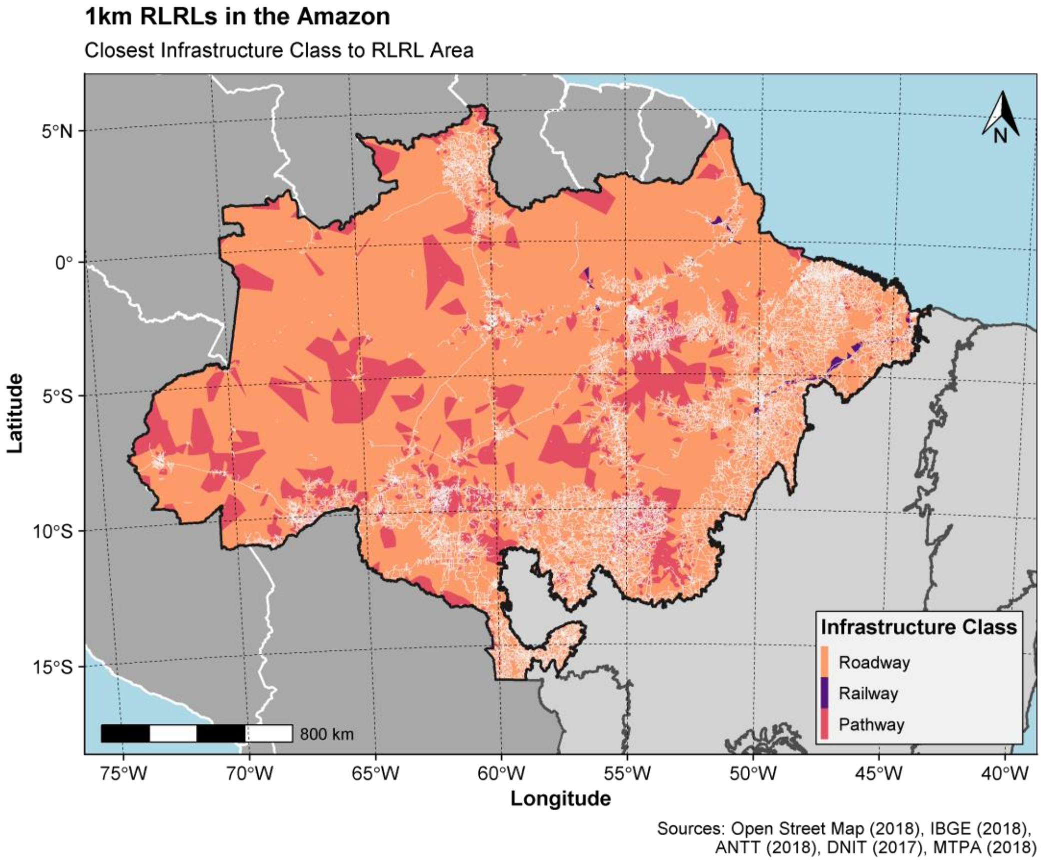


Amazon 1 km RLRL areas dominated by specific infrastructure types (for the simplified decision tree, see how variable *RLRL_Infra* was determined in R scripts **3A** in **Data S3**).

**Fig. S49.**

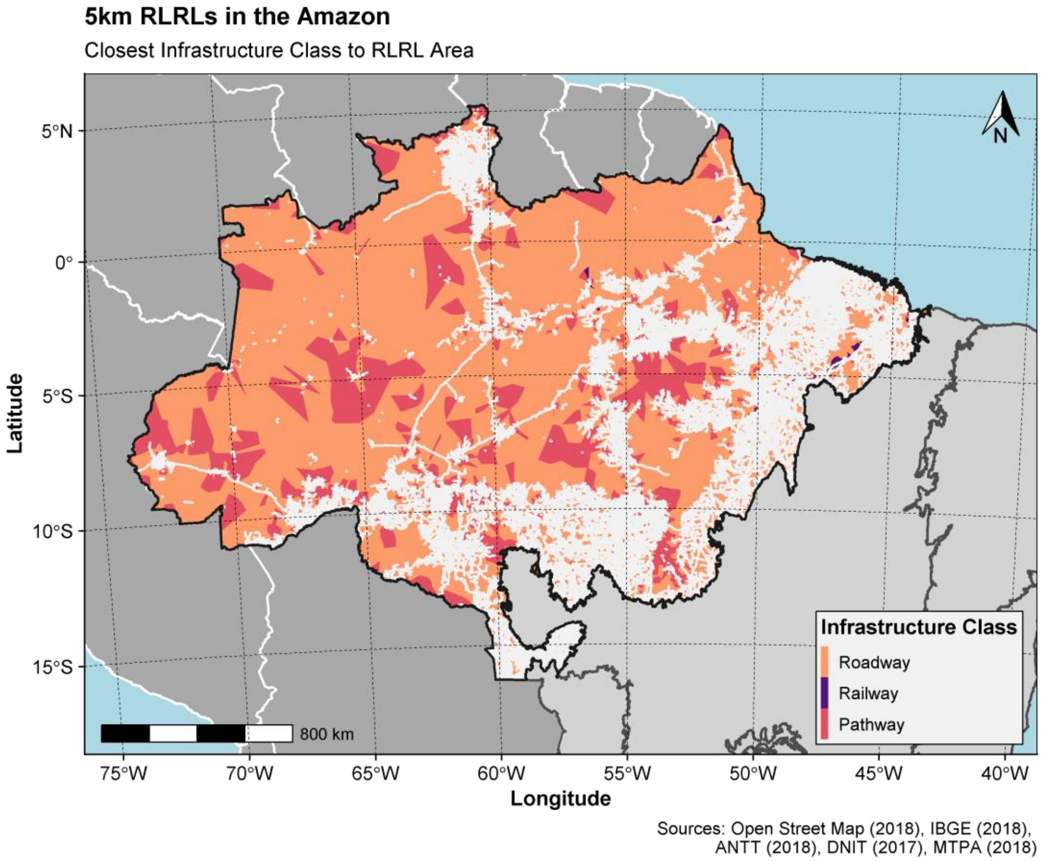


Amazon 5 km RLRL areas dominated by specific infrastructure types (for the simplified decision tree, see how variable *RLRL_Infra* was determined in R scripts **3A** in **Data S3**).

**Fig. S50.**

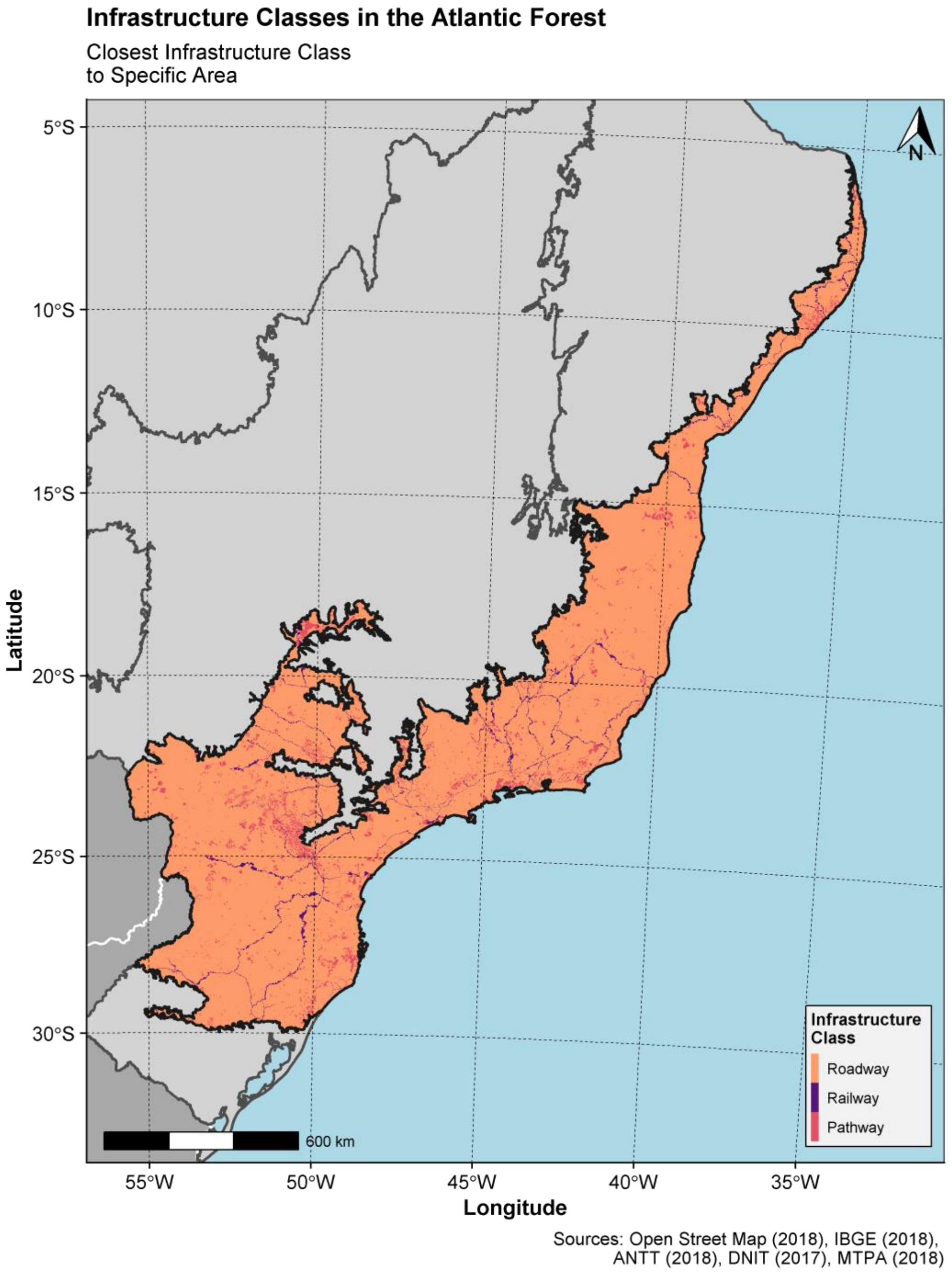


Atlantic Forest areas dominated by specific infrastructure types (for the simplified decision tree, see how variable *RLRL_Infra* was determined in R scripts **3A** in **Data S3**).

**Fig. S51.**

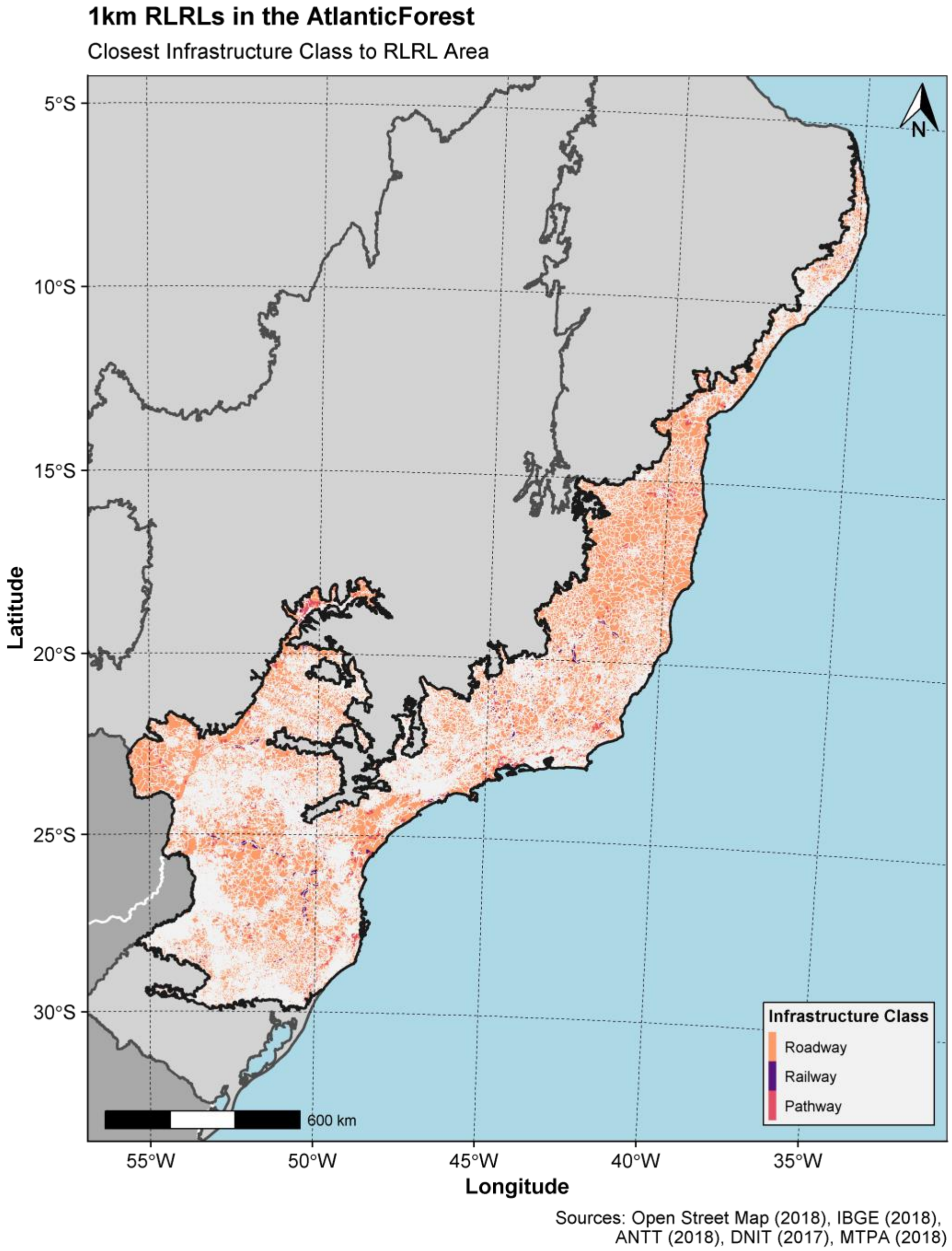


Atlantic Forest 1 km RLRL areas dominated by specific infrastructure types (for the simplified decision tree, see how variable *RLRL_Infra* was determined in R scripts **3A** in **Data S3**).

**Fig. S52.**

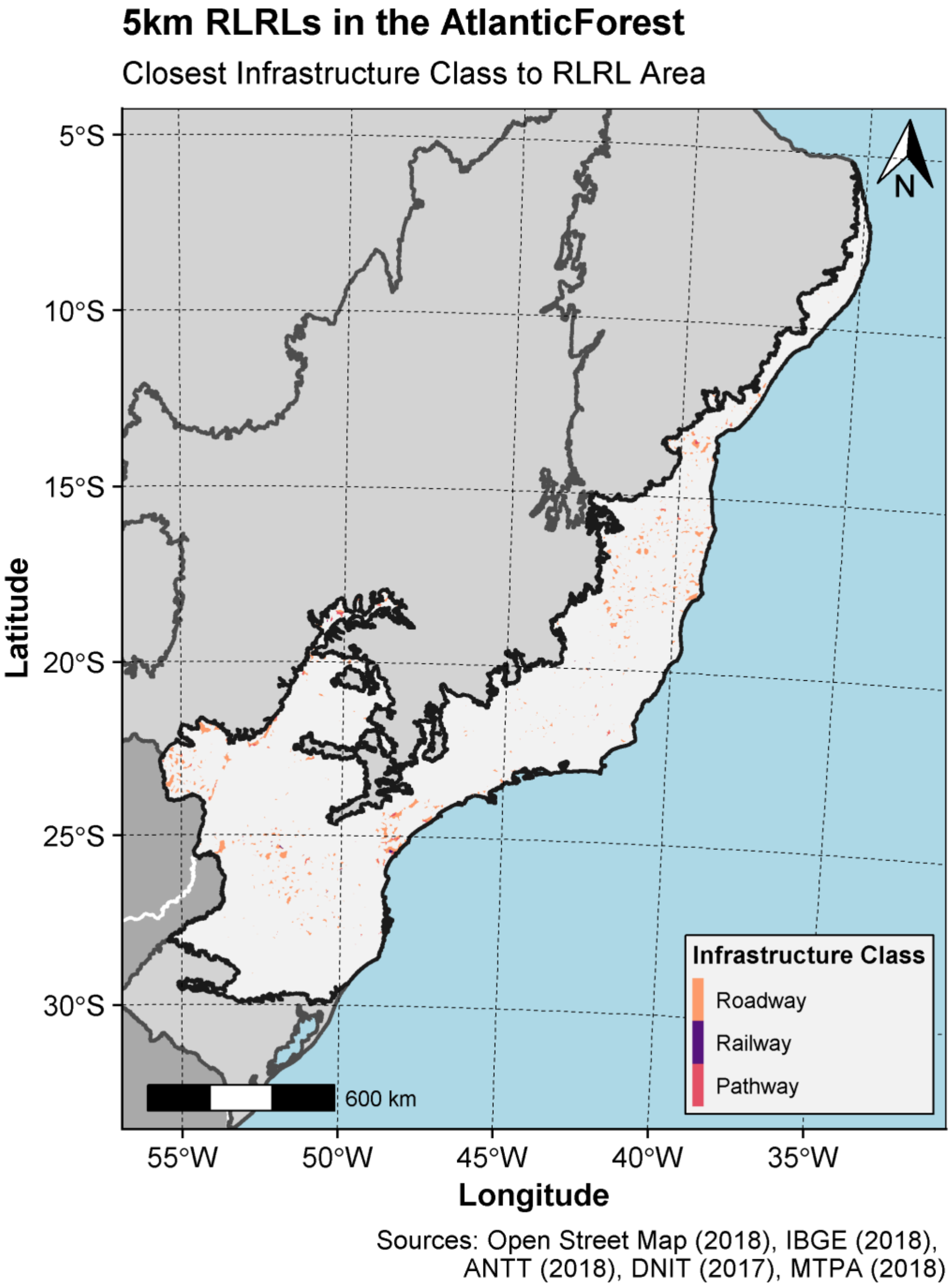


Atlantic Forest 5 km RLRL areas dominated by specific infrastructure types (for the simplified decision tree, see how variable *RLRL_Infra* was determined in R scripts **3A** in **Data S3**).

**Fig. S53.**

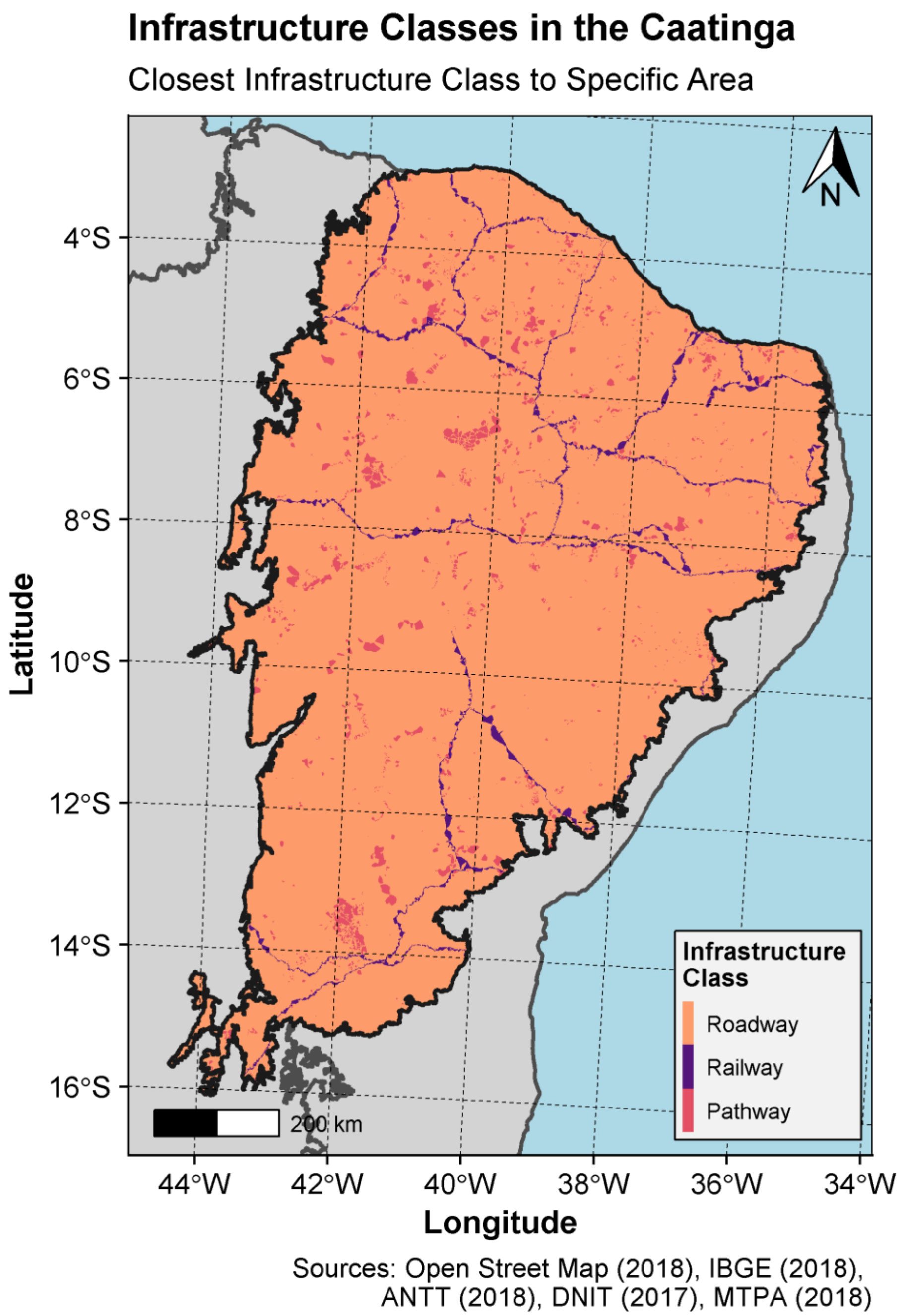


Caatinga areas dominated by specific infrastructure types (for the simplified decision tree, see how variable *RLRL_Infra* was determined in R scripts **3A** in **Data S3**).

**Fig. S54.**

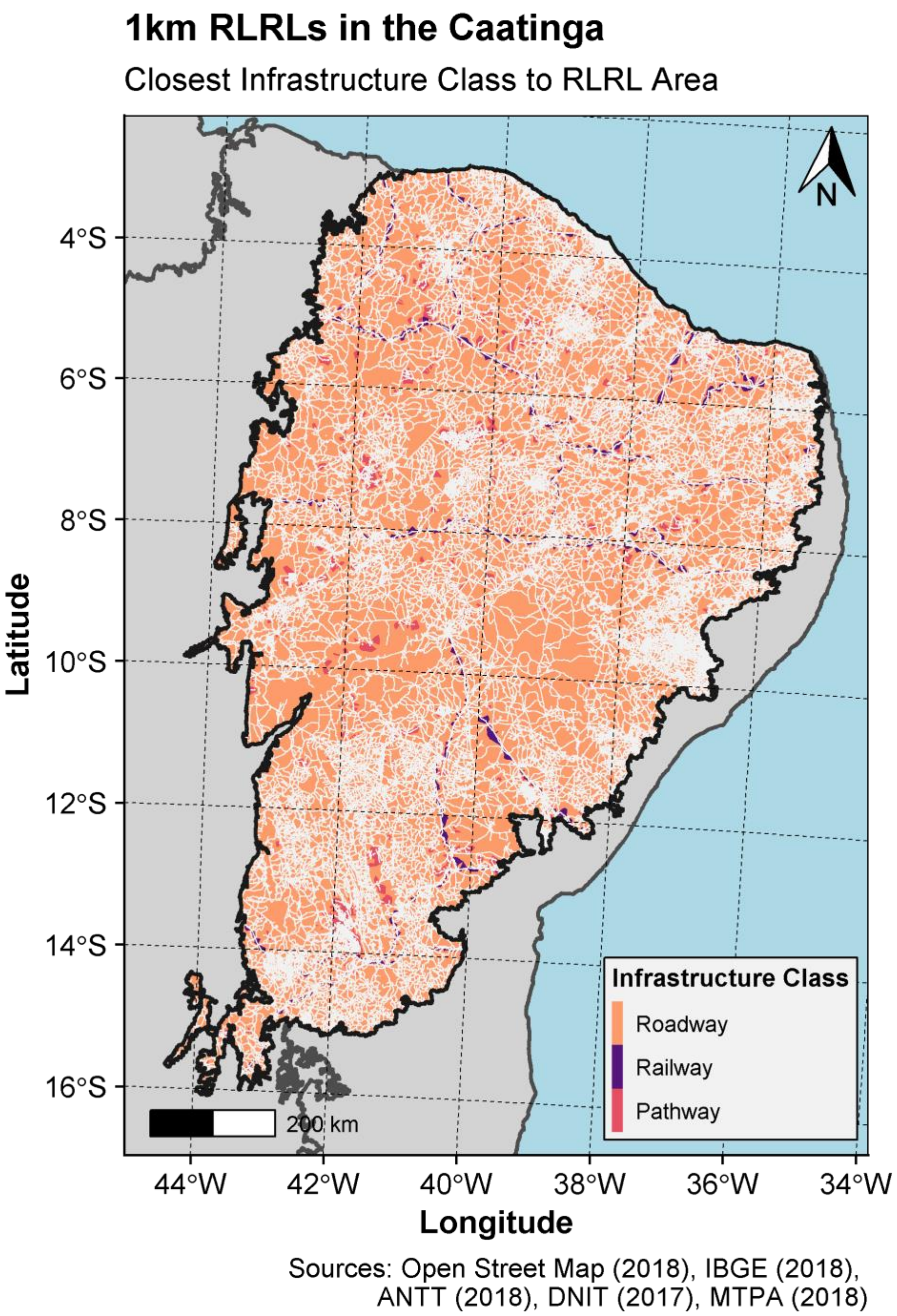


Caatinga 1 km RLRL areas dominated by specific infrastructure types (for the simplified decision tree, see how variable *RLRL_Infra* was determined in R scripts **3A** in **Data S3**).

**Fig. S55.**

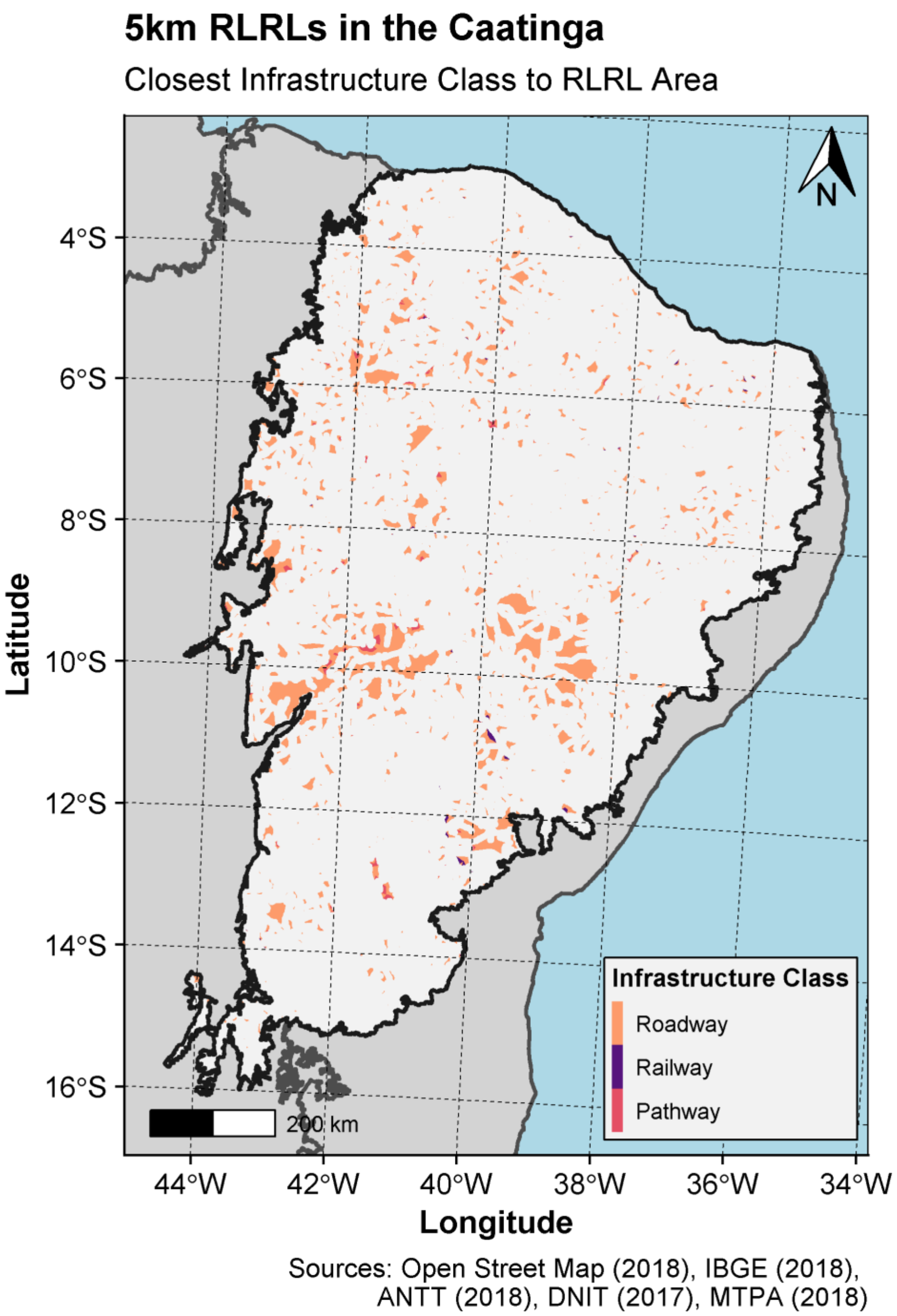


Caatinga 5 km RLRL areas dominated by specific infrastructure types (for the simplified decision tree, see how variable *RLRL_Infra* was determined in R scripts **3A** in **Data S3**).

**Fig. S56.**

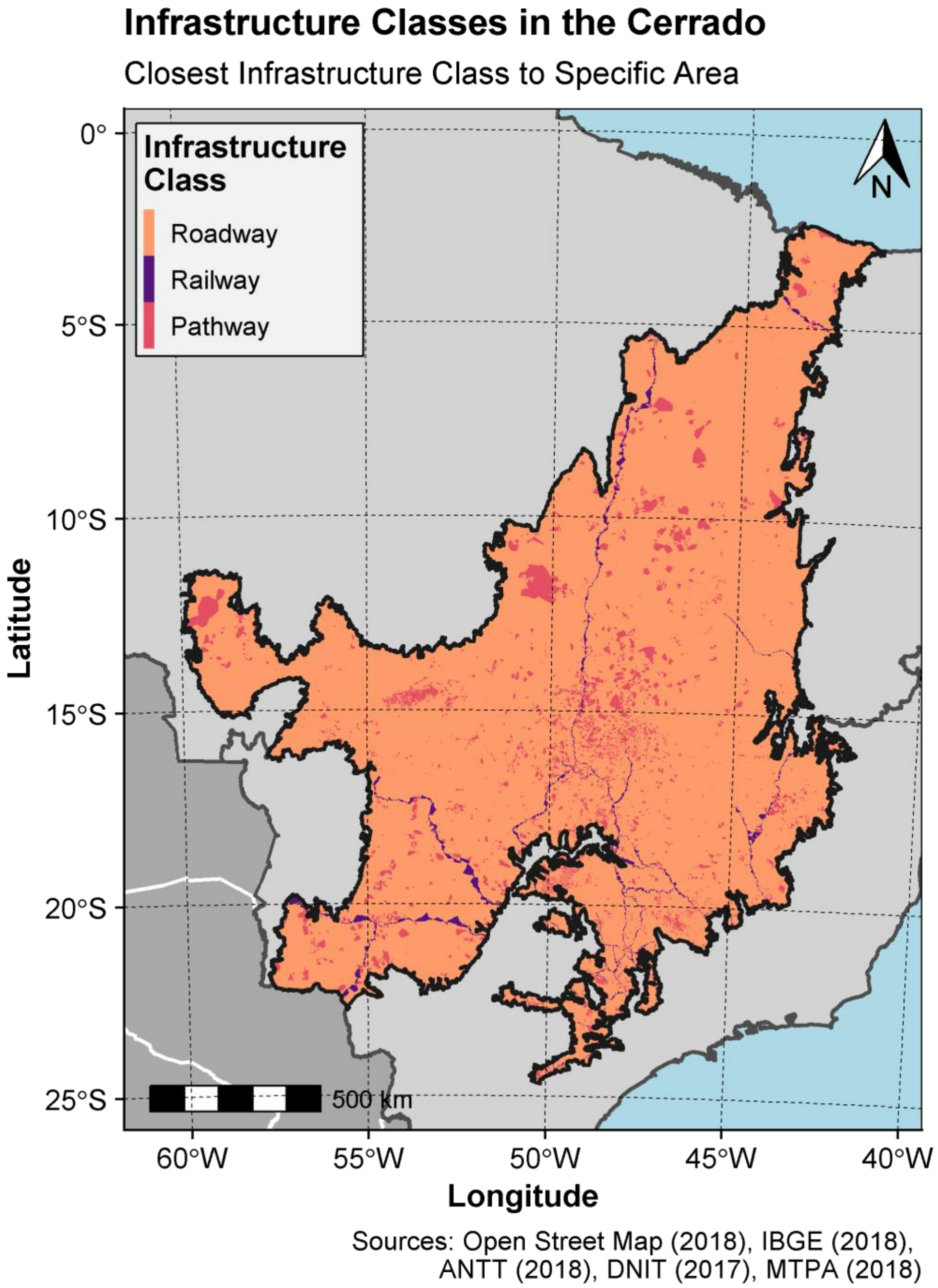


Cerrado areas dominated by specific infrastructure types (for the simplified decision tree, see how variable *RLRL_Infra* was determined in R scripts **3A** in **Data S3**).

**Fig. S57.**

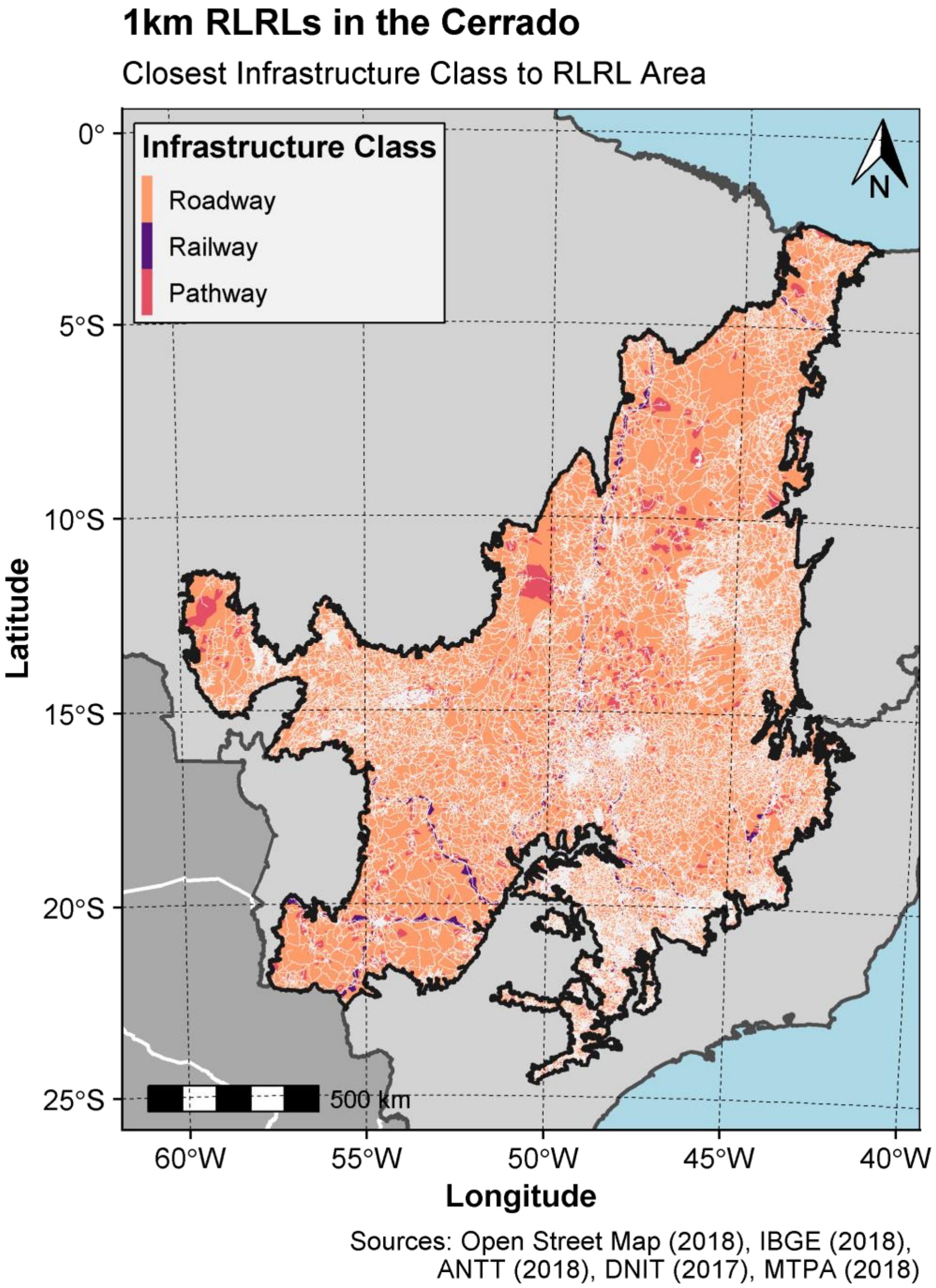


Cerrado 1 km RLRL areas dominated by specific infrastructure types (for the simplified decision tree, see how variable *RLRL_Infra* was determined in R scripts **3A** in **Data S3**).

**Fig. S58.**

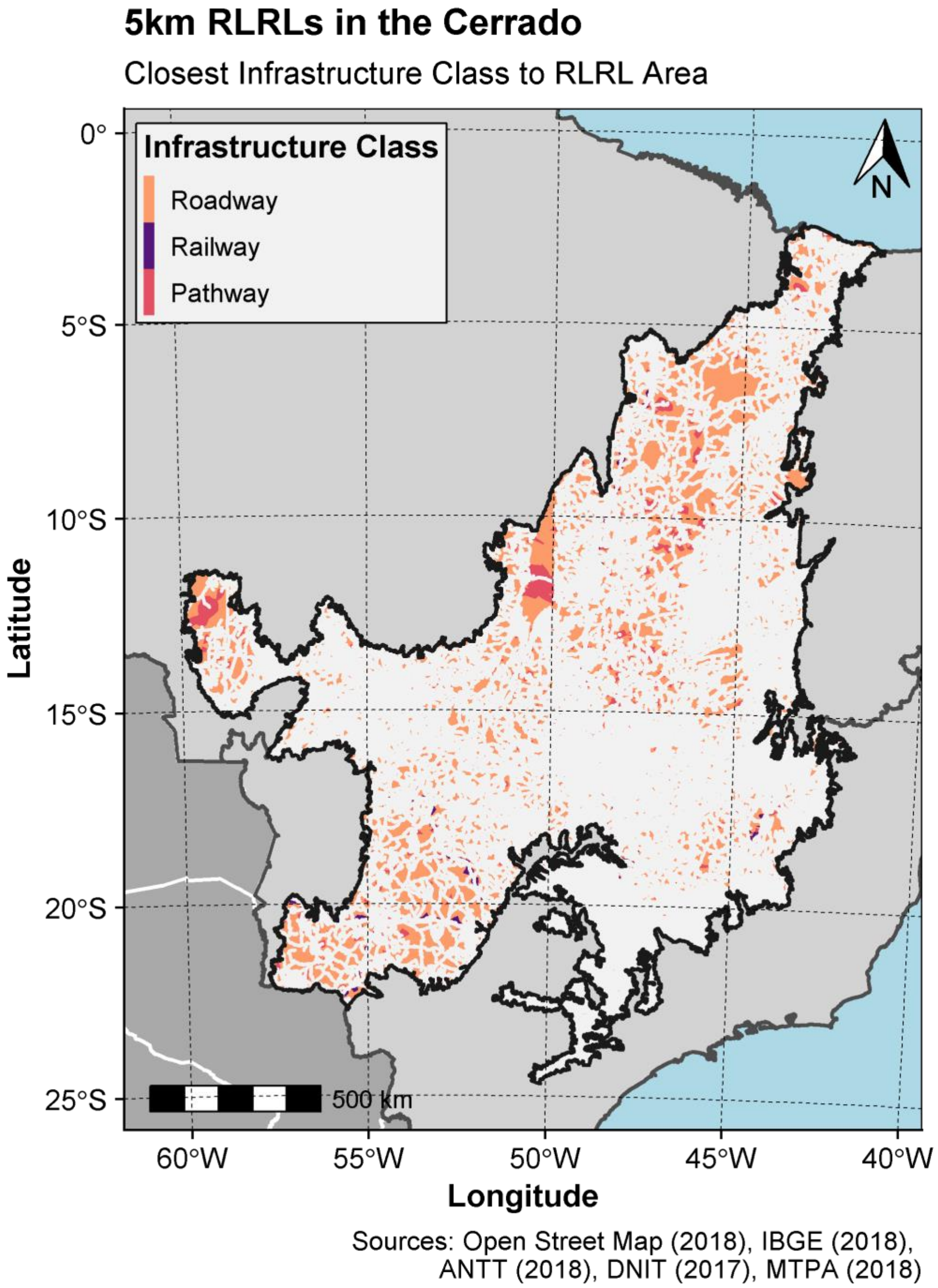


Cerrado 5 km RLRL areas dominated by specific infrastructure types (for the simplified decision tree, see how variable *RLRL_Infra* was determined in R scripts **3A** in **Data S3**).

**Fig. S59.**

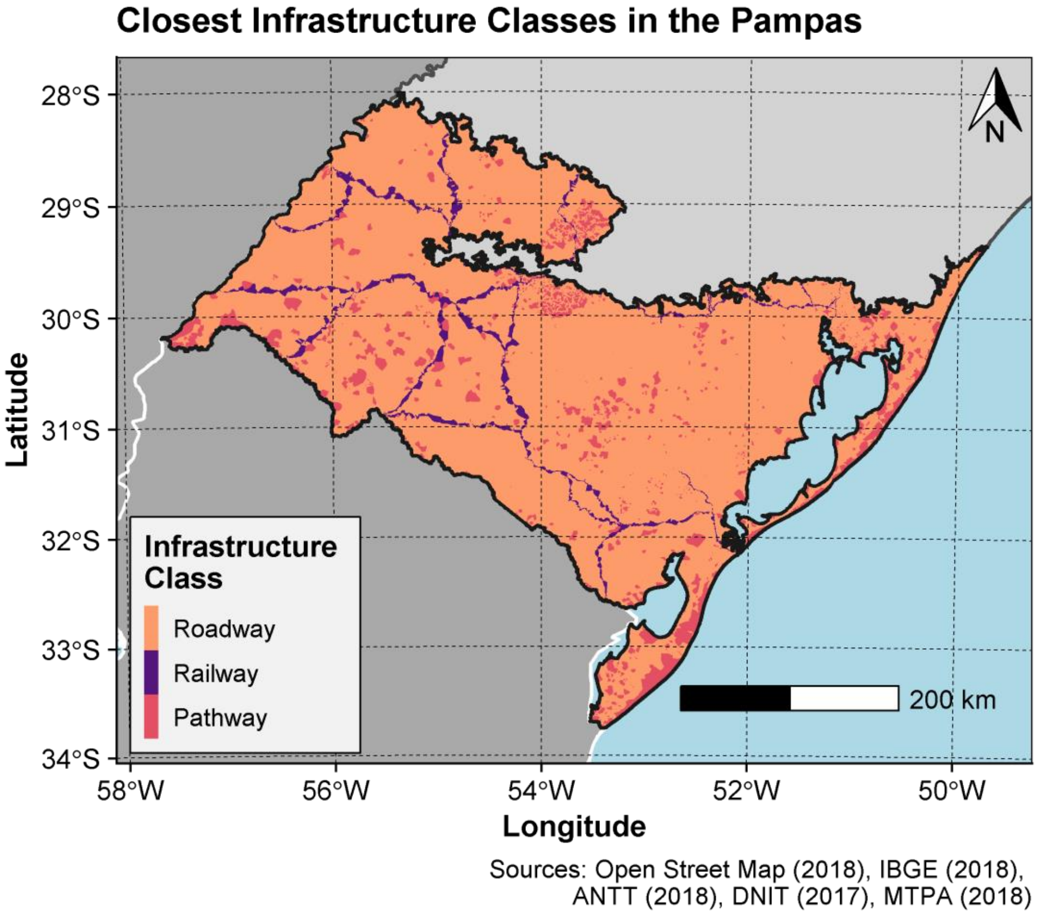


Pampas areas dominated by specific infrastructure types (for the simplified decision tree, see how variable *RLRL_Infra* was determined in R scripts **3A** in **Data S3**).

**Fig. S60.**

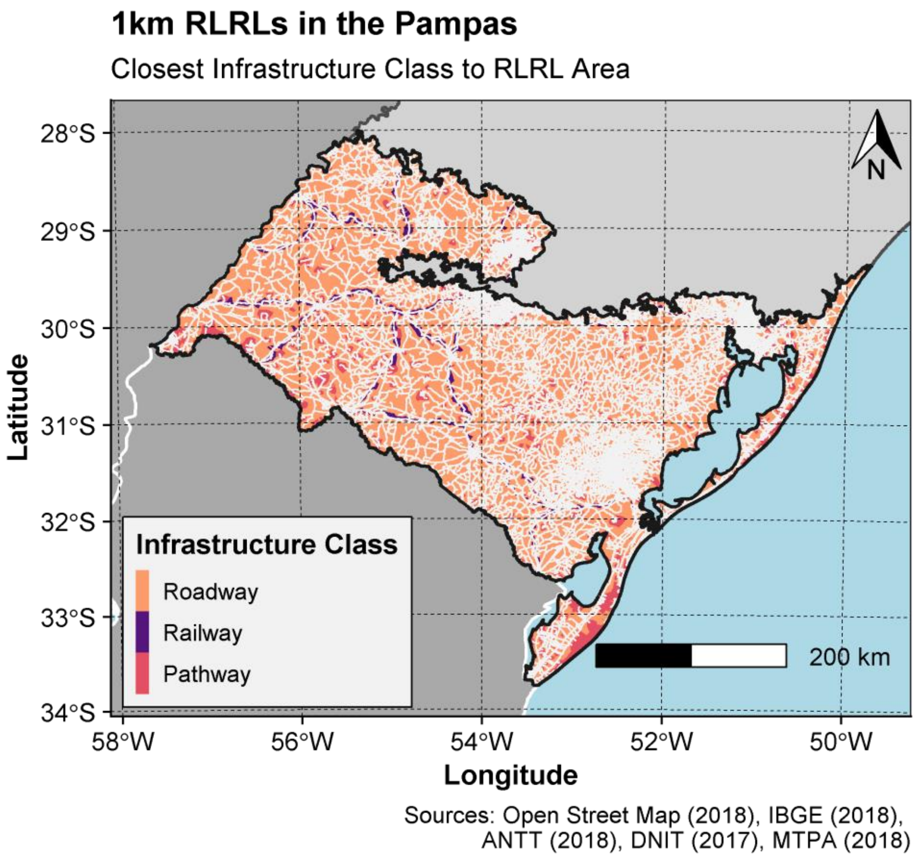


Pampas 1 km RLRL areas dominated by specific infrastructure types (for the simplified decision tree, see how variable *RLRL_Infra* was determined in R scripts **3A** in **Data S3**).

**Fig. S60.**

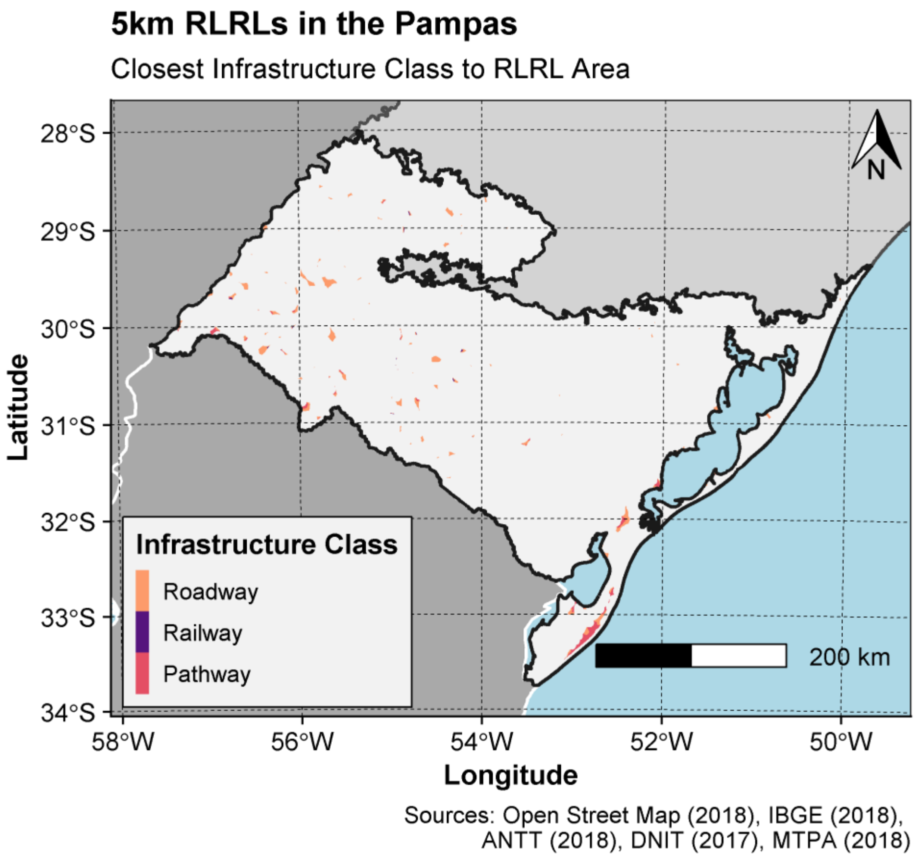


Pampas 5 km RLRL areas dominated by specific infrastructure types (for the simplified decision tree, see how variable *RLRL_Infra* was determined in R scripts **3A** in **Data S3**).

**Fig. S62.**

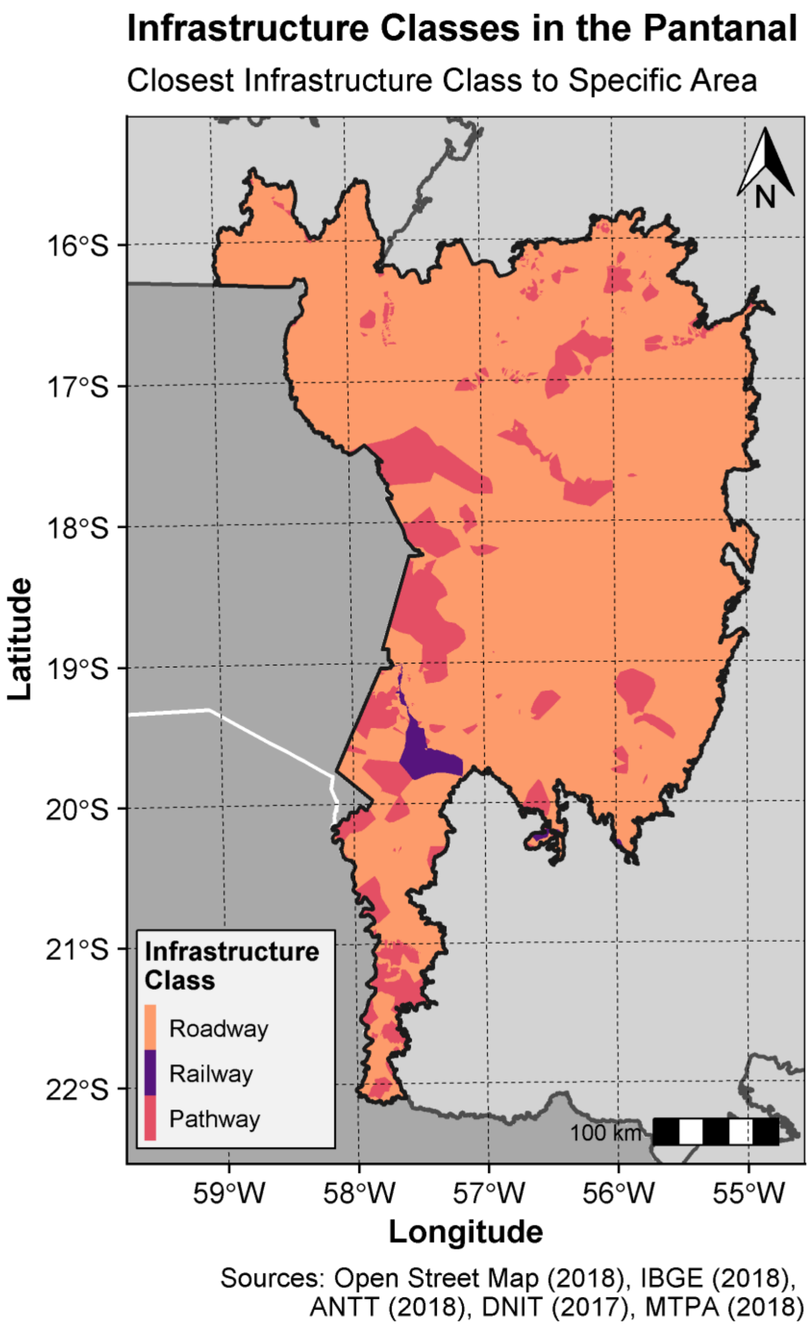


Pantanal areas dominated by specific infrastructure types (for the simplified decision tree, see how variable *RLRL_Infra* was determined in R scripts **3A** in **Data S3**).

**Fig. S63.**

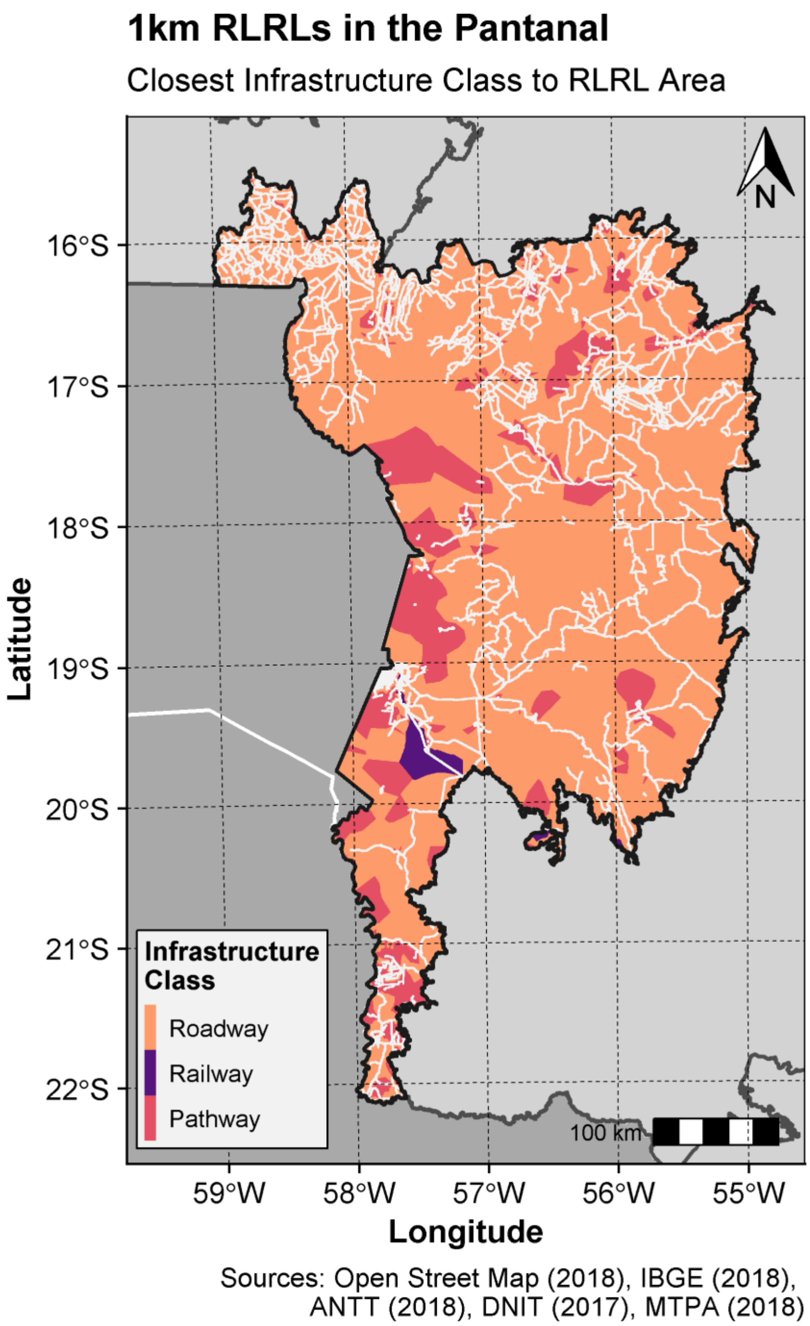


Pantanal 1 km RLRL areas dominated by specific infrastructure types (for the simplified decision tree, see how variable *RLRL_Infra* was determined in R scripts **3A** in **Data S3**).

**Fig. S64.**

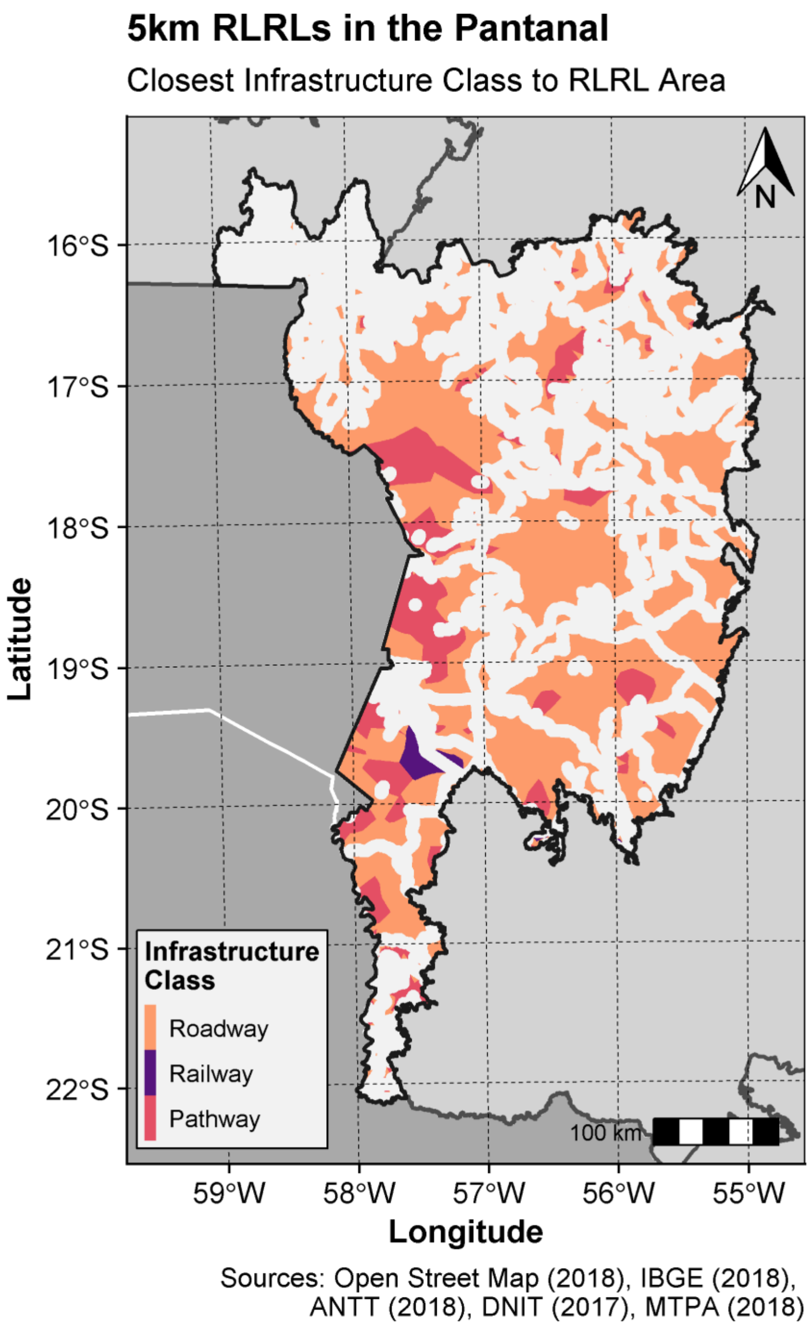


Pantanal 5 km RLRL areas dominated by specific infrastructure types (for the simplified decision tree, see how variable *RLRL_Infra* was determined in R scripts **3A** in **Data S3**).

**Table S1.**

| Study Area Spatial Data Sources | | | | |
|---|---|---|---|---|
| **Spatial Data** | **Source** | **Date** | **Scale** | **Format and other Information** |
| **Territorial Extent of Brazil** *(Used for initial Country wide modeling)* | IBGE, 2017 (*66*) | 2017 | 1:250,000 | Vector Shapefile |
| **Biomes of Brazil** *(MapBiomas improved adaptation of IBGE's official Biome Boundaries)* | Rosa & Project MapBiomas, 2016 (*65*) | 2016 | 1:250,000 | Vector Shapefile |

Model Study Area Spatial Data Sources

**Table S2.**

| **IBGE's Albers Equal Area Conic Projection** (*69*) | | |
|---|---|---|
| Projection: Albers<br>False_Easting: 5000000,0<br>False_Northing: 10000000,0<br>Central_Meridian: -54,0<br>Standard_Parallel_1: -2,0<br>Standard_Parallel_2: -22,0<br>Latitude_Of_Origin: -12,0 | Linear Unit: Meter (1,0)<br>Geographic Coordinate System:<br>GCS_SIRGAS_2000<br>Angular Unit: Degree (0,0174532925199433)<br>Prime Meridian: Greenwich (0,0)<br>Datum: D_SIRGAS_2000 | Spheroid: GRS_1980<br>Semimajor Axis: 6378137,0<br>Semiminor Axis: 6356752,314140356<br>Inverse Flattening: 298,257222101 |

IBGE Projection Configurations on Study Area

**Table S3.**

| Data Name | Source | Scale | Area in HA | Difference to IBGE's National Territory Spatial Data |
|---|---|---|---|---|
| Lim_Unidade_Federacao (Official Shapefile for State and National Borders) | IBGE, 2017 (*66*) | 1:250,000 | 851,419,849 | - |
| Mapa de Biomas do Brasil (Official Shapefile for Brazil's Terrestrial Biomes) | IBGE, 2006 (*73*) | 1:5,000,000 | 848,737,340 | 2,682,508.97 (ha) 26,825.09 ($km^2$) |
| Mapa de Limite dos Biomas 1:250,000 (MapBiomas Adaptation of Brazil's Terrestrial Biomes based on Official IBGE Data) | Rosa & Project MapBiomas, 2016 (*65*) | 1:250,000 | 850,273,851 | 1,145,997.35 (ha) 11,459.97 ($km^2$) |
| Study Area | (Overlapping of IBGE, 2017 (*66*) and Rosa & Project MapBiomas, 2016 (*65*) | 1:250,000 | 849,957,610 | 1,146,997.35 (ha) 11,469.97 ($km^2$) |

Breakdown of Discrepancies in IBGE's Official Territorial Data, Biome Data, and MapBiomas Adaptations of IBGE's Biome Delineations, and the designated Study Area.

**Table S4.**

| Open Street Map Road Variables Included in Input Data | |
|---|---|
| **Automobile Dominated** | a. Motorway & Motorway link<br>b. Trunk & Trunk link<br>c. Primary & Primary link<br>d. Secondary & Secondary link<br>e. Tertiary & Tertiary link<br>f. Unclassified<br>g. Residential<br>h. Service |
| **Not Automobile Dominated** | a. Living Street<br>b. Pedestrian<br>c. Track |
| **Paths Category** | a. Footway<br>b. Bridleway<br>c. Steps<br>d. Path |
| **Others** | a. Cycleway<br>b. Busway |

Open Street Map Roadway Categories Included in the RLRL Modeling OpenStreetMap Wiki, 2019 (*85*)(*99*)

**Table S5.**

| Spatial Data Sources for Modeling Roadless and Railroad-less (RLRL) Areas | | | | |
|---|---|---|---|---|
| **Spatial Data** | **Source** | **Temporal Resolution / Representation Date** | **Scale** | **Format and other Information** |
| **Open Street Map - Roads** *(Brazil's Road Data)* | GeoFabrik & OpenStreetMap, 2018 (*70*) | May 2018 | Unknown *Suspected to be equivalent to or Larger Scale than 1:250,000* | Vector Shapefile |
| **Base Cartográfica Continua do Brasil – BC250** *(National Road Data)* | IBGE, 2017 (*66*) | 2017 | 1:250,000 | Vector Shapefile |
| **Open Street Map - Railroads** *(Brazil's Railroad Data)* | GeoFabrik & OpenStreetMap, 2018(*70*) | May 2018 | Unknown *Suspected to be equivalent to or larger scale than 1:250,000* | Vector Shapefile |
| **Railroads in the Transportation Information Database** *(Ferrovias do Banco de Informações de Transporte – BIT)* | MTPA, 2018 (*74*) | 2018 | Unknown *Suspected 1:5,000,000* | Vector Shapefile |
| **Georeferenced Federal Railroad Network** *(Malha Ferroviária Federal Georreferenciada)* | ANTT, 2016 (*75*) | 2017 | Unknown *Suspected 1:250,000* | Vector Shapefile |
| **Base Cartográfica Continua do Brasil – BC250** *(National Railroad Data)* | IBGE, 2017 (*66*) | 2017 | 1:250,000 | Vector Shapefile |

Spatial Data Sources for Modeling Roadless and Railroad-less (RLRL) Areas

**Table S6.**

| Spatial Data Sources for Analyzing Environmental and Biodiversity Conservation Policy Variables | | | | |
|---|---|---|---|---|
| **Spatial Data** | **Source** | **Temporal Resolution / Representation Date** | **Scale** | **Format and other Information** |
| **Conservation Units – Federal, State and Municipal** *(Unidades de Conservação da Cadastro Nacional de Unidades de Conservação)* | MMA, 2018 (*38*) | 2018 | Varies between 1:5.000 to 1:100.000 | Vector Shapefile |
| **Indigenous Territories** *(Terras Indígenas)* | FUNAI, 2018 (*39*) | 2018 | Unknown *(Suspected 1:250,000)* | Vector Shapefile |
| **Quilombos** *(Maroon Community Lands)* | INCRA, 2018 (*40*) | 2018 | Unknown *(Suspected 1:250,000)* | Vector Shapefile |
| **Priority Areas for Biodiversity Conservation** *(Áreas Prioritárias para Conservação da Biodiversidade)* | MMA, 2018 (*98*) | 2018 | Unknown *(Suspected 1:250,000)* | Vector Shapefile |
| **Native Vegetation Formations, Abiotic Areas, and Anthropogenic Areas** *(Classified Landsat imagery from Project MapBiomes)* | Project MapBiomas, 2018 (*42*) | 2017 | 30-meter resolution upscaled to 250-meter resolution | Raster upscaled and then transformed to Vector Shapefile |

Spatial Data Sources for Analyzing Environmental and Biodiversity Conservation Policy Variables

**Table S7.**

| Geoprocessing Tools | Description |
|---|---|
| **Clip** | To cut the various geographic input data according to the established study area and feature of interest. |
| **Erase** | To erase away various unneeded geographic information and leaving desired geographic input data available for use. |
| **Intersect** | To identify and result in desired combinations of overlapping geographic input variables. |
| **Union** | To create a geographic union of overlapping features and so that they maintain all attribute data. |
| **Merge** | To combine multiple geographic input variables into the same file. |
| **Calculate Geometry** | To calculate the area or distance of geographic variables of interest for the study. |
| **Euclidean Distance** | To generate a raster file showing the continuous distance of areas away from identified Road and Railroad features. |
| **Conversion Tools** | To convert raster data to vector data and vice-versa. |

Basic Geoprocessing Operations for the RLRL Modeling and Results Analysis

**Table S8.**

| Biomes | Biome Area (ha) | 1km RLRL Area (ha) | Percent of Biome Area Covered by 1km RLRL Areas | 5km RLRL Area (ha) | Percent of Biome Area Covered by 5km RLRL Areas |
|---|---|---|---|---|---|
| Amazon | 421,386,509 | 373,200,745 | 88.56% | 287,589,987 | 68.25% |
| Atlantic Forest | 110,596,318 | 41,195,936 | 37.25% | 2,552,609 | 2.31% |
| Caatinga | 83,594,452 | 44,506,033 | 53.24% | 5,032,180 | 6.02% |
| Cerrado | 202,937,475 | 128,064,733 | 63.11% | 31,072,937 | 15.31% |
| Pampas | 16,432,052 | 7,360,547 | 44.79% | 211,301 | 1.29% |
| Pantanal | 15,010,803 | 12,177,264 | 81.12% | 6,290,184 | 41.90% |
| National Total | 849,957,610 | 606,505,258 | 71.36% | 332,749,198 | 39.15% |

RLRL Areas as Percent of Biome Specific Area (Biome Area as Denominator).

**Table S9.**

| Biomes | Biome Area (ha) | Protected 1km RLRL Area (ha) | Percent of Biome Area Covered by Protected 1km RLRL Areas | Protected 5km RLRL Area (ha) | Percent of Biome Area Covered by Protected 5km RLRL Areas |
|---|---|---|---|---|---|
| Amazon | 421,386,509 | 203,096,831 | 48.20% | 186,841,107 | 44.34% |
| Atlantic Forest | 110,596,318 | 4,681,795 | 4.23% | 593,442 | 0.54% |
| Caatinga | 83,594,452 | 4,939,738 | 5.91% | 1,279,743 | 1.53% |
| Cerrado | 202,937,475 | 16,668,070 | 8.21% | 7,944,508 | 3.91% |
| Pampas | 16,432,052 | 295,645 | 1.80% | 17,729 | 0.11% |
| Pantanal | 15,010,803 | 567,538 | 3.78% | 406,979 | 2.71% |
| National Total | 849,957,610 | 230,249,617 | 27.09% | 197,083,508 | 23.19% |

Protected RLRL Areas as Percent of Biome Specific Area (Biome Area as Denominator).

**Table S10.**

| Biomes | Biome Area (ha) | Unprotected* 1km RLRL Area (ha) | Percent of Biome Area Covered by Unprotected* 1km RLRL Areas | Unprotected* 5km RLRL Area (ha) | Percent of Biome Area Covered by Unprotected* 5km RLRL Areas |
|---|---|---|---|---|---|
| Amazon | 421,386,509 | 170,103,914 | 40.37% | 100,748,880 | 23.91% |
| Atlantic Forest | 110,596,318 | 36,514,141 | 33.02% | 1,959,167 | 1.77% |
| Caatinga | 83,594,452 | 39,566,295 | 47.33% | 3,752,437 | 4.49% |
| Cerrado | 202,937,475 | 111,396,662 | 54.89% | 23,128,429 | 11.40% |
| Pampas | 16,432,052 | 7,064,902 | 42.99% | 193,572 | 1.18% |
| Pantanal | 15,010,803 | 11,609,727 | 77.34% | 5,883,205 | 39.19% |
| National Total | 849,957,610 | 376,255,640 | 44.27% | 135,665,690 | 15.96% |

Unprotected RLRL Areas as Percent of Biome Specific Area (Biome Area as Denominator). * Unprotected areas are outside of Legally Protected Areas (LPAs), and therefore are located predominantly on private lands, government owned lands that are not conservation focused, or lands of unknown tenure status.

**Table S11.**

| Biomes | Biome Area (ha) | Biome Level Remaining Native Vegetation (ha) | Percent of Biome Area Covered by Remaining Native Vegetation |
|---|---|---|---|
| Amazon | 421,386,509 | 349,502,078 | 82.94% |
| Atlantic Forest | 110,596,318 | 34,583,015 | 31.27% |
| Caatinga | 83,594,452 | 51,272,144 | 61.33% |
| Cerrado | 202,937,475 | 111,835,766 | 55.11% |
| Pampas | 16,432,052 | 9,379,999 | 57.08% |
| Pantanal | 15,010,803 | 11,799,943 | 78.61% |
| National Total | 849,957,610 | 568,372,944 | 66.87% |

Biome Area and Remaining Native Vegetation (Biome Area as Denominator).

**Table S12.**

| Biomes | Biome Area (ha) | Biome Level Abiotic Areas (ha) | Percent of Biome Area Covered by Abiotic Areas |
|---|---|---|---|
| Amazon | 421,386,509 | 22,146,586 | 5.26% |
| Atlantic Forest | 110,596,318 | 2,172,707 | 1.96% |
| Caatinga | 83,594,452 | 632,632 | 0.76% |
| Cerrado | 202,937,475 | 1,675,307 | 0.83% |
| Pampas | 16,432,052 | 547,128 | 3.33% |
| Pantanal | 15,010,803 | 608,352 | 4.05% |
| National Total | 849,957,610 | 27,782,713 | 3.27% |

Biome Area and Abiotic Areas (Biome Area as Denominator).

**Table S13.**

| Biomes | Biome Area (ha) | Biome Level Anthropic Areas (ha) | Percent of Biome Area Covered by Anthropic Areas |
|---|---|---|---|
| Amazon | 421,386,509 | 49,251,326 | 11.69% |
| Atlantic Forest | 110,596,318 | 73,328,742 | 66.30% |
| Caatinga | 83,594,452 | 31,366,503 | 37.52% |
| Cerrado | 202,937,475 | 89,291,528 | 44.00% |
| Pampas | 16,432,052 | 6,476,154 | 39.41% |
| Pantanal | 15,010,803 | 2,559,710 | 17.05% |
| National Total | 849,957,610 | 252,273,963 | 29.68% |

Biome Area and Anthropic Areas (Biome Area as Denominator).

**Table S14.**

| Biomes | Biome Area (ha) | Biome Level 1km RLRL Native Vegetation (ha) | Percent of Biome Area Covered by 1km RLRL Native Vegetation | Biome Level 5km RLRL Native Vegetation (ha) | Percent of Biome Area Covered by 5km RLRL Native Vegetation |
|---|---|---|---|---|---|
| Amazon | 421,386,509 | 325,266,562 | 77.19% | 267,489,018 | 63.48% |
| Atlantic Forest | 110,596,318 | 15,157,309 | 13.71% | 1,141,769 | 1.03% |
| Caatinga | 83,594,452 | 30,473,237 | 36.45% | 3,709,996 | 4.44% |
| Cerrado | 202,937,475 | 78,301,073 | 38.58% | 22,169,604 | 10.92% |
| Pampas | 16,432,052 | 4,157,750 | 25.30% | 103,901 | 0.63% |
| Pantanal | 15,010,803 | 9,904,395 | 65.98% | 5,240,239 | 34.91% |
| National Total | 849,957,610 | 463,260,327 | 54.50% | 299,854,527 | 35.28% |

RLRL Areas and Remaining Native Vegetation (Biome Area as Denominator).

**Table S15.**

| Biomes | Biome Area (ha) | Biome Level 1km RLRL Abiotic Areas (ha) | Percent of Biome Area Covered by 1km RLRL Abiotic Areas | Biome Level 5km RLRL Abiotic Areas (ha) | Percent of Biome Area Covered by 5km RLRL Abiotic Areas |
|---|---|---|---|---|---|
| Amazon | 421,386,509 | 19,516,817 | 4.63% | 14,265,770 | 3.39% |
| Atlantic Forest | 110,596,318 | 1,387,056 | 1.25% | 219,542 | 0.20% |
| Caatinga | 83,594,452 | 419,596 | 0.50% | 134,569 | 0.16% |
| Cerrado | 202,937,475 | 1,242,764 | 0.61% | 263,219 | 0.13% |
| Pampas | 16,432,052 | 374,655 | 2.28% | 51,632 | 0.31% |
| Pantanal | 15,010,803 | 568,795 | 3.79% | 415,072 | 2.77% |
| National Total | 849,957,610 | 23,509,684 | 2.77% | 15,349,804 | 1.81% |

RLRL Areas and Abiotic Areas (Biome Area as Denominator).

**Table S16.**

| Biomes | Biome Area (ha) | Biome Level 1km RLRL Anthropic Areas (ha) | Percent of Biome Area Covered by 1km RLRL Anthropic Areas | Biome Level 5km RLRL Anthropic Areas (ha) | Percent of Biome Area Covered by 5km RLRL Anthropic Areas |
|---|---|---|---|---|---|
| Amazon | 421,386,509 | 28,083,796 | 6.66% | 5,616,450 | 1.33% |
| Atlantic Forest | 110,596,318 | 24,516,164 | 22.17% | 1,182,255 | 1.07% |
| Caatinga | 83,594,452 | 13,449,077 | 16.09% | 1,170,777 | 1.40% |
| Cerrado | 202,937,475 | 48,448,676 | 23.87% | 8,625,811 | 4.25% |
| Pampas | 16,432,052 | 2,813,918 | 17.12% | 53,878 | 0.33% |
| Pantanal | 15,010,803 | 1,672,611 | 11.14% | 619,814 | 4.13% |
| National Total | 849,957,610 | 118,984,243 | 14.00% | 17,268,987 | 2.03% |

RLRL Areas and Anthropic Areas (Biome Area as Denominator).

**Table S17.**

| Biomes | Biome Area (ha) | Biome Level Protected 1km RLRL Native Vegetation (ha) | Percent of Biome Area Covered by Protected 1km RLRL Native Vegetation | Biome Level Protected 5km RLRL Native Vegetation (ha) | Percent of Biome Area Covered by Protected 5km RLRL Native Vegetation |
|---|---|---|---|---|---|
| Amazon | 421,386,509 | 193,130,032 | 45.83% | 179,536,338 | 42.61% |
| Atlantic Forest | 110,596,318 | 3,337,316 | 3.02% | 520,155 | 0.47% |
| Caatinga | 83,594,452 | 3,933,776 | 4.71% | 983,579 | 1.18% |
| Cerrado | 202,937,475 | 14,963,654 | 7.37% | 7,557,287 | 3.72% |
| Pampas | 16,432,052 | 249,241 | 1.52% | 13,899 | 0.08% |
| Pantanal | 15,010,803 | 475,935 | 3.17% | 330,611 | 2.20% |
| National Total | 849,957,610 | 216,089,953 | 25.42% | 188,941,868 | 22.23% |

RLRL Areas and Remaining Protected Native Vegetation (Biome Area as Denominator).

**Table S18.**

| Biomes | Biome Area (ha) | Biome Level Protected 1km RLRL Abiotic Areas (ha) | Percent of Biome Area Covered by Protected 1km RLRL Abiotic Areas | Biome Level Protected 5km RLRL Abiotic Areas (ha) | Percent of Biome Area Covered by Protected 5km RLRL Abiotic Areas |
|---|---|---|---|---|---|
| Amazon | 421,386,509 | 8,485,189 | 2.01% | 6,631,745 | 1.57% |
| Atlantic Forest | 110,596,318 | 269,000 | 0.24% | 35,350 | 0.03% |
| Caatinga | 83,594,452 | 207,709 | 0.25% | 127,741 | 0.15% |
| Cerrado | 202,937,475 | 289,545 | 0.14% | 117,615 | 0.06% |
| Pampas | 16,432,052 | 18,357 | 0.11% | 1,247 | 0.01% |
| Pantanal | 15,010,803 | 79,876 | 0.53% | 73,478 | 0.49% |
| National Total | 849,957,610 | 9,349,676 | 1.10% | 6,987,177 | 0.82% |

RLRL Areas and Remaining Protected Abiotic Areas (Biome Area as Denominator).

**Table S19.**

| Biomes | Biome Area (ha) | Biome Level Protected 1km RLRL Anthropic Areas (ha) | Percent of Biome Area Covered by Protected 1km RLRL Anthropic Areas | Biome Level Protected 5km RLRL Anthropic Areas (ha) | Percent of Biome Area Covered by Protected 5km RLRL Anthropic Areas |
|---|---|---|---|---|---|
| Amazon | 421,386,509 | 1,334,815 | 0.32% | 554,229 | 0.13% |
| Atlantic Forest | 110,596,318 | 1,057,824 | 0.96% | 35,525 | 0.03% |
| Caatinga | 83,594,452 | 779,447 | 0.93% | 165,327 | 0.20% |
| Cerrado | 202,937,475 | 1,404,928 | 0.69% | 264,549 | 0.13% |
| Pampas | 16,432,052 | 27,787 | 0.17% | 2,545 | 0.02% |
| Pantanal | 15,010,803 | 10,752 | 0.07% | 2,355 | 0.02% |
| National Total | 849,957,610 | 4,615,553 | 0.54% | 1,024,530 | 0.12% |

RLRL Areas and Remaining Protected Anthropic Areas (Biome Area as Denominator).

**Table S20.**

| Biomes | Biome Area (ha) | Biome Level Unprotected 1km RLRL Native Vegetation (ha) | Percent of Biome Area Covered by Unprotected* 1km RLRL Native Vegetation | Biome Level Unprotected 5km RLRL Native Vegetation (ha) | Percent of Biome Area Covered by Unprotected* 5km RLRL Native Vegetation |
|---|---|---|---|---|---|
| Amazon | 421,386,509 | 132,136,531 | 31.36% | 87,952,681 | 20.87% |
| Atlantic Forest | 110,596,318 | 11,819,994 | 10.69% | 621,614 | 0.56% |
| Caatinga | 83,594,452 | 26,539,462 | 31.75% | 2,726,417 | 3.26% |
| Cerrado | 202,937,475 | 63,337,419 | 31.21% | 14,612,317 | 7.20% |
| Pampas | 16,432,052 | 3,908,509 | 23.79% | 90,002 | 0.55% |
| Pantanal | 15,010,803 | 9,428,460 | 62.81% | 4,909,628 | 32.71% |
| National Total | 849,957,610 | 247,170,374 | 29.08% | 110,912,659 | 13.05% |

RLRL Areas and Remaining Unprotected Native Vegetation (Biome Area as Denominator). * Unprotected areas are outside of Legally Protected Areas (LPAs), and therefore are located predominantly on private lands, government owned lands that are not conservation focused, or lands of unknown tenure status.

**Table S21.**

| Biomes | Biome Area (ha) | Biome Level Unprotected 1km RLRL Abiotic Areas (ha) | Percent of Biome Area Covered by Unprotected* 1km RLRL Abiotic Areas | Biome Level Unprotected 5km RLRL Abiotic Areas (ha) | Percent of Biome Area Covered by Unprotected* 5km RLRL Abiotic Areas |
|---|---|---|---|---|---|
| Amazon | 421,386,509 | 11,031,628 | 2.62% | 7,634,025 | 1.81% |
| Atlantic Forest | 110,596,318 | 1,118,056 | 1.01% | 184,192 | 0.17% |
| Caatinga | 83,594,452 | 211,887 | 0.25% | 6,828 | 0.01% |
| Cerrado | 202,937,475 | 953,220 | 0.47% | 145,604 | 0.07% |
| Pampas | 16,432,052 | 356,299 | 2.17% | 50,385 | 0.31% |
| Pantanal | 15,010,803 | 488,919 | 3.26% | 341,594 | 2.28% |
| National Total | 849,957,610 | 14,160,008 | 1.67% | 8,362,627 | 0.98% |

RLRL Areas and Remaining Unprotected Abiotic Areas (Biome Area as Denominator). * Unprotected areas are outside of Legally Protected Areas (LPAs), and therefore are located predominantly on private lands, government owned lands that are not conservation focused, or lands of unknown tenure status.

**Table S22.**

| Biomes | Biome Area (ha) | Biome Level Unprotected 1km RLRL Anthropic Areas (ha) | Percent of Biome Area Covered by Unprotected* 1km RLRL Anthropic Areas | Biome Level Unprotected 5km RLRL Anthropic Areas (ha) | Percent of Biome Area Covered by Unprotected* 5km RLRL Anthropic Areas |
|---|---|---|---|---|---|
| Amazon | 421,386,509 | 26,748,981 | 6.35% | 5,062,222 | 1.20% |
| Atlantic Forest | 110,596,318 | 23,458,339 | 21.21% | 1,146,730 | 1.04% |
| Caatinga | 83,594,452 | 12,669,631 | 15.16% | 1,005,451 | 1.20% |
| Cerrado | 202,937,475 | 47,043,748 | 23.18% | 8,361,262 | 4.12% |
| Pampas | 16,432,052 | 2,786,131 | 16.96% | 51,333 | 0.31% |
| Pantanal | 15,010,803 | 1,661,860 | 11.07% | 617,459 | 4.11% |
| National Total | 849,957,610 | 114,368,690 | 13.46% | 16,244,456 | 1.91% |

RLRL Areas and Remaining Unprotected Anthropic Areas (Biome Area as Denominator). * Unprotected areas are outside of Legally Protected Areas (LPAs), and therefore are located predominantly on private lands, government owned lands that are not conservation focused, or lands of unknown tenure status.

**Table S23.**

| Biomes | Biome Area (ha) | Biome Level Unprotected* PABC Area (ha) | Percent of Biome Area Designated as Unprotected* PABCs |
|---|---|---|---|
| Amazon | 421,386,509 | 98,314,766 | 23.33% |
| Atlantic Forest | 110,596,318 | 15,950,058 | 14.42% |
| Caatinga | 83,594,452 | 26,060,751 | 31.18% |
| Cerrado | 202,937,475 | 63,651,068 | 31.36% |
| Pampas | 16,432,052 | 3,651,346 | 22.22% |
| Pantanal | 15,010,803 | 7,056,083 | 47.01% |
| National Total | 849,957,610 | 214,684,072 | 25.26% |

Biome Areas and Unprotected Priority Areas for Biodiversity Conservation (PABCs) (Biome Area as Denominator). * Unprotected areas are outside of Legally Protected Areas (LPAs), and therefore are located predominantly on private lands, government owned lands that are not conservation focused, or lands of unknown tenure status.

**Table S24.**

| Biomes | Biome Area (ha) | Biome Level Native Vegetation in PABCs (ha) | Percent of Biome Area Covered by PABC Native Vegetation |
|---|---|---|---|
| Amazon | 421,386,509 | 71,690,495 | 17.01% |
| Atlantic Forest | 110,596,318 | 5,794,379 | 5.24% |
| Caatinga | 83,594,452 | 16,545,243 | 19.79% |
| Cerrado | 202,937,475 | 38,420,748 | 18.93% |
| Pampas | 16,432,052 | 2,556,202 | 15.56% |
| Pantanal | 15,010,803 | 5,692,518 | 37.92% |
| National Total | 849,957,610 | 140,699,586 | 16.55% |

Biome Areas and Native Vegetation in Unprotected Priority Areas for Biodiversity Conservation (PABCs) (Biome Area as Denominator).

**Table S25.**

| Biomes | Biome Area (ha) | Biome Level Abiotic Areas in PABCs (ha) | Percent of Biome Area Covered by PABC Abiotic Areas |
|---|---|---|---|
| Amazon | 421,386,509 | 6,655,937 | 1.58% |
| Atlantic Forest | 110,596,318 | 216,277 | 0.20% |
| Caatinga | 83,594,452 | 210,497 | 0.25% |
| Cerrado | 202,937,475 | 643,301 | 0.32% |
| Pampas | 16,432,052 | 60,908 | 0.37% |
| Pantanal | 15,010,803 | 167,308 | 1.11% |
| National Total | 849,957,610 | 7,954,228 | 0.94% |

Biome Areas and Abiotic Areas in Unprotected Priority Areas for Biodiversity Conservation (PABCs) (Biome Area as Denominator).

**Table S26.**

| Biomes | Biome Area (ha) | Biome Level Anthropic Areas in PABCs (ha) | Percent of Biome Area Covered by PABC Anthropic Areas |
|---|---|---|---|
| Amazon | 421,386,509 | 19,831,561 | 4.71% |
| Atlantic Forest | 110,596,318 | 9,873,510 | 8.93% |
| Caatinga | 83,594,452 | 9,186,403 | 10.99% |
| Cerrado | 202,937,475 | 24,556,523 | 12.10% |
| Pampas | 16,432,052 | 1,029,557 | 6.27% |
| Pantanal | 15,010,803 | 1,176,617 | 7.84% |
| National Total | 849,957,610 | 65,654,171 | 7.72% |

Biome Areas and Anthropic Areas in Unprotected Priority Areas for Biodiversity Conservation (PABCs) (Biome Area as Denominator).

**Table S27.**

| Biomes | Biome Area (ha) | Biome Level Overlapping Unprotected* PABCs & 1km RLRL Areas (ha) | Percent of Biome Area Covered by Overlapping Unprotected* PABCs & 1km RLRL Areas | Biome Level Overlapping Unprotected* PABCs & 5km RLRL Areas (ha) | Percent of Biome Area Covered by Overlapping Unprotected* PABCs & 5km RLRL Areas |
|---|---|---|---|---|---|
| Amazon | 421,386,509 | 78,721,412 | 18.68% | 47,501,326 | 11.27% |
| Atlantic Forest | 110,596,318 | 6,442,430 | 5.83% | 432,767 | 0.39% |
| Caatinga | 83,594,452 | 13,702,354 | 16.39% | 1,451,545 | 1.74% |
| Cerrado | 202,937,475 | 41,163,064 | 20.28% | 9,327,073 | 4.60% |
| Pampas | 16,432,052 | 1,715,872 | 10.44% | 37,966 | 0.23% |
| Pantanal | 15,010,803 | 5,709,883 | 38.04% | 2,778,177 | 18.51% |
| National Total | 849,957,610 | 147,455,016 | 17.35% | 61,528,854 | 7.24% |

RLRL Areas and Unprotected Priority Areas for Biodiversity Conservation (PABCs) (Biome Area as Denominator). * Unprotected areas are outside of Legally Protected Areas (LPAs), and therefore are located predominantly on private lands, government owned lands that are not conservation focused, or lands of unknown tenure status.

**Table S28.**

| Biomes | Biome Area (ha) | Biome Level Native Vegetation in Overlapping Unprotected* PABCs & 1km RLRLs (ha) | Percent of Biome Area Covered by Native Vegetation in Overlapping Unprotected* PABCs & 1km RLRL Areas | Biome Level Native Vegetation in Overlapping Unprotected* PABCs & 5km RLRL Areas (ha) | Percent of Biome Area Covered by Native Vegetation in Overlapping Unprotected* PABCs & 5km RLRL Areas |
|---|---|---|---|---|---|
| Amazon | 421,386,509 | 62,226,990 | 14.77% | 41,700,182 | 9.90% |
| Atlantic Forest | 110,596,318 | 2,647,126 | 2.39% | 160,628 | 0.15% |
| Caatinga | 83,594,452 | 9,707,447 | 11.61% | 1,113,756 | 1.33% |
| Cerrado | 202,937,475 | 26,783,483 | 13.20% | 6,747,348 | 3.32% |
| Pampas | 16,432,052 | 1,193,432 | 7.26% | 23,864 | 0.15% |
| Pantanal | 15,010,803 | 4,752,044 | 31.66% | 2,377,982 | 15.84% |
| National Total | 849,957,610 | 107,310,522 | 12.63% | 52,123,760 | 6.13% |

RLRL Areas, Unprotected Priority Areas for Biodiversity Conservation (PABCs) and Remaining Native Vegetation Cover (Biome Area as Denominator). * Unprotected areas are outside of Legally Protected Areas (LPAs), and therefore are located predominantly on private lands, government owned lands that are not conservation focused, or lands of unknown tenure status.

**Table S29.**

| Biomes | Biome Area (ha) | Biome Level Abiotic Areas in Overlapping Unprotected* PABCs & 1km RLRLs (ha) | Percent of Biome Area Covered by Abiotic Areas in Overlapping Unprotected* PABCs & 1km RLRLs | Biome Level Abiotic Areas in Overlapping Unprotected* PABCs & 5km RLRLs (ha) | Percent of Biome Area Covered by Abiotic Areas in Overlapping Unprotected* PABCs & 5km RLRLs |
|---|---|---|---|---|---|
| Amazon | 421,386,509 | 5,629,168 | 1.34% | 3,784,328 | 0.90% |
| Atlantic Forest | 110,596,318 | 124,770 | 0.11% | 18,632 | 0.02% |
| Caatinga | 83,594,452 | 115,013 | 0.14% | 2,963 | 0.00% |
| Cerrado | 202,937,475 | 475,425 | 0.23% | 86,469 | 0.04% |
| Pampas | 16,432,052 | 38,774 | 0.24% | 3,172 | 0.02% |
| Pantanal | 15,010,803 | 152,571 | 1.02% | 101,210 | 0.67% |
| National Total | 849,957,610 | 6,535,721 | 0.77% | 3,996,775 | 0.47% |

RLRL Areas and Unprotected Priority Areas for Biodiversity Conservation (PABCs) and Abiotic Areas (Biome Area as Denominator). * Unprotected areas are outside of Legally Protected Areas (LPAs), and therefore are located predominantly on private lands, government owned lands that are not conservation focused, or lands of unknown tenure status.

**Table S30.**

| Biomes | Biome Area (ha) | Biome Level Anthropic Areas in Overlapping Unprotected* PABCs & 1km RLRLs (ha) | Percent of Biome Area Covered by Anthropic Areas in Overlapping Unprotected* PABCs & 1km RLRLs | Biome Level Anthropic Areas in Overlapping Unprotected* PABCs & 5km RLRLs (ha) | Percent of Biome Area Covered by Anthropic Areas in Overlapping Unprotected* PABCs & 5km RLRLs |
|---|---|---|---|---|---|
| Amazon | 421,386,509 | 10,784,801 | 2.56% | 1,975,653 | 0.47% |
| Atlantic Forest | 110,596,318 | 3,650,992 | 3.30% | 252,376 | 0.23% |
| Caatinga | 83,594,452 | 3,820,441 | 4.57% | 330,184 | 0.39% |
| Cerrado | 202,937,475 | 13,887,704 | 6.84% | 2,490,483 | 1.23% |
| Pampas | 16,432,052 | 481,028 | 2.93% | 10,620 | 0.06% |
| Pantanal | 15,010,803 | 791,473 | 5.27% | 292,918 | 1.95% |
| National Total | 849,957,610 | 33,416,440 | 3.93% | 5,352,233 | 0.63% |

RLRL Areas and Unprotected Priority Areas for Biodiversity Conservation (PABCs) and Anthropic Areas (Biome Area as Denominator). * Unprotected areas are outside of Legally Protected Areas (LPAs), and therefore are located predominantly on private lands, government owned lands that are not conservation focused, or lands of unknown tenure status.

**Table S31.**

| Biomes | Total 1km RLRL Area per Biome (ha) | Protected** 1km RLRL Area (ha) | Percent of 1km RLRL Area Designated as Protected** | Unprotected* 1km RLRL Area (ha) | Percent of 1km RLRL Area that is Unprotected* |
|---|---|---|---|---|---|
| Amazon | 373,200,745 | 203,096,831 | 54.42% | 170,103,914 | 45.58% |
| Atlantic Forest | 41,195,936 | 4,681,795 | 11.36% | 36,514,141 | 88.64% |
| Caatinga | 44,506,033 | 4,939,738 | 11.10% | 39,566,295 | 88.90% |
| Cerrado | 128,064,733 | 16,668,070 | 13.02% | 111,396,662 | 86.98% |
| Pampas | 7,360,547 | 295,645 | 4.02% | 7,064,902 | 95.98% |
| Pantanal | 12,177,264 | 567,538 | 4.66% | 11,609,727 | 95.34% |
| National Total | 606,505,258 | 230,249,617 | 37.96% | 376,255,640 | 62.04% |

Protected and Unprotected 1km RLRL Areas as Percent of Overall 1km RLRL Area per Biome (RLRL Area as Denominator). * Unprotected areas are outside of Legally Protected Areas (LPAs), and therefore are located predominantly on private lands, government owned lands that are not conservation focused, or lands of unknown tenure status. **Protected areas are inside of Legally Protected Areas (LPAs) which include all of Brazil's protected areas in the National System of Conservation Units, titled Indigenous Territories and Maroon Community Lands.

**Table S32.**

| Biomes | Total 5km RLRL Area per Biome (ha) | Protected** 5km RLRL Area (ha) | Proportion of 5km RLRL Area per Biome that is Protected** | Unprotected* 5km RLRL Area (ha) | Proportion of 5km RLRL Area per Biome that is Unprotected* |
|---|---|---|---|---|---|
| Amazon | 287,589,987 | 186,841,107 | 64.97% | 100,748,880 | 35.03% |
| Atlantic Forest | 2,552,609 | 593,442 | 23.25% | 1,959,167 | 76.75% |
| Caatinga | 5,032,180 | 1,279,743 | 25.43% | 3,752,437 | 74.57% |
| Cerrado | 31,072,937 | 7,944,508 | 25.57% | 23,128,429 | 74.43% |
| Pampas | 211,301 | 17,729 | 8.39% | 193,572 | 91.61% |
| Pantanal | 6,290,184 | 406,979 | 6.47% | 5,883,205 | 93.53% |
| National Total | 332,749,198 | 197,083,508 | 59.23% | 135,665,690 | 40.77% |

Protected and Unprotected 5km RLRL Areas as Percent of Overall 5km RLRL Area per Biome (RLRL Area as Denominator). * Unprotected areas are outside of Legally Protected Areas (LPAs), and therefore are located predominantly on private lands, government owned lands that are not conservation focused, or lands of unknown tenure status. **Protected areas are inside of Legally Protected Areas (LPAs) which include all of Brazil's protected areas in the National System of Conservation Units, titled Indigenous Territories and Maroon Community Lands

**Table S33.**

| Biomes | Total 1km RLRL Area per Biome (ha) | Biome Level 1km RLRL Native Vegetation (ha) | Percent of 1km RLRL Area Covered by Native Vegetation |
|---|---|---|---|
| Amazon | 373,200,745 | 325,266,562 | 87.16% |
| Atlantic Forest | 41,195,936 | 15,157,309 | 36.79% |
| Caatinga | 44,506,033 | 30,473,237 | 68.47% |
| Cerrado | 128,064,733 | 78,301,073 | 61.14% |
| Pampas | 7,360,547 | 4,157,750 | 56.49% |
| Pantanal | 12,177,264 | 9,904,395 | 81.34% |
| National Total | 606,505,258 | 463,260,327 | 76.38% |

Native Vegetated 1km RLRL Area as Percent of Overall 1km RLRL Area per Biome (RLRL Area as Denominator).

**Table S34.**

| Biomes | Total 1km RLRL Area per Biome (ha) | Biome Level 1km RLRL Abiotic Areas (ha) | Percent of 1km RLRL Area Covered by Abiotic Areas |
|---|---|---|---|
| Amazon | 373,200,745 | 19,516,817 | 5.23% |
| Atlantic Forest | 41,195,936 | 1,387,056 | 3.37% |
| Caatinga | 44,506,033 | 419,596 | 0.94% |
| Cerrado | 128,064,733 | 1,242,764 | 0.97% |
| Pampas | 7,360,547 | 374,655 | 5.09% |
| Pantanal | 12,177,264 | 568,795 | 4.67% |
| National Total | 606,505,258 | 23,509,684 | 3.88% |

Abiotic Areas 1km RLRL Area as Percent of Overall 1km RLRL Area per Biome (RLRL Area as Denominator).

**Table S35.**

| Biomes | Total 1km RLRL Area per Biome (ha) | Biome Level 1km RLRL Anthropic Areas (ha) | Percent of 1km RLRL Area Covered by Anthropic Areas |
|---|---|---|---|
| Amazon | 373,200,745 | 28,083,796 | 7.53% |
| Atlantic Forest | 41,195,936 | 24,516,164 | 59.51% |
| Caatinga | 44,506,033 | 13,449,077 | 30.22% |
| Cerrado | 128,064,733 | 48,448,676 | 37.83% |
| Pampas | 7,360,547 | 2,813,918 | 38.23% |
| Pantanal | 12,177,264 | 1,672,611 | 13.74% |
| National Total | 606,505,258 | 118,984,243 | 19.62% |

Anthropic Areas 1km RLRL Area as Percent of Overall 1km RLRL Area per Biome (RLRL Area as Denominator).

**Table S36.**

| Biomes | Total 5km RLRL Area per Biome (ha) | Biome Level 5km RLRL Native Vegetation (ha) | Percent of 5km RLRL Area Covered by Native Vegetation |
|---|---|---|---|
| Amazon | 287,589,987 | 267,489,018 | 93.01% |
| Atlantic Forest | 2,552,609 | 1,141,769 | 44.73% |
| Caatinga | 5,032,180 | 3,709,996 | 73.73% |
| Cerrado | 31,072,937 | 22,169,604 | 71.35% |
| Pampas | 211,301 | 103,901 | 49.17% |
| Pantanal | 6,290,184 | 5,240,239 | 83.31% |
| National Total | 332,749,198 | 299,854,527 | 90.11% |

Native Vegetated 5km RLRL Area as Percent of Overall 5km RLRL Area per Biome (RLRL Area as Denominator).

**Table S37.**

| Biomes | Total 5km RLRL Area per Biome (ha) | Biome Level 5km RLRL Abiotic Areas (ha) | Percent of 5km RLRL Area Covered by Abiotic Areas |
|---|---|---|---|
| Amazon | 287,589,987 | 14,265,770 | 4.96% |
| Atlantic Forest | 2,552,609 | 219,542 | 8.60% |
| Caatinga | 5,032,180 | 134,569 | 2.67% |
| Cerrado | 31,072,937 | 263,219 | 0.85% |
| Pampas | 211,301 | 51,632 | 24.44% |
| Pantanal | 6,290,184 | 415,072 | 6.60% |
| National Total | 332,749,198 | 15,349,804 | 4.61% |

Abiotic Areas 5km RLRL Area as Percent of Overall 5km RLRL Area per Biome (RLRL Area as Denominator).

**Table S38.**

| Biomes | Total 5km RLRL Area per Biome (ha) | Biome Level 5km RLRL Anthropic Areas (ha) | Percent of 5km RLRL Area Covered by Abiotic Areas |
|---|---|---|---|
| Amazon | 287,589,987 | 5,616,450 | 4.96% |
| Atlantic Forest | 2,552,609 | 1,182,255 | 8.60% |
| Caatinga | 5,032,180 | 1,170,777 | 2.67% |
| Cerrado | 31,072,937 | 8,625,811 | 0.85% |
| Pampas | 211,301 | 53,878 | 24.44% |
| Pantanal | 6,290,184 | 619,814 | 6.60% |
| National Total | 332,749,198 | 17,268,987 | 4.61% |

Anthropic Areas 5km RLRL Area as Percent of Overall 5km RLRL Area per Biome (RLRL Area as Denominator).

**Table S39.**

| Biomes | Total 1km RLRL Area per Biome (ha) | Biome Level Protected 1km RLRL Native Vegetation (ha) | Percent of 1km RLRL Area Covered by Protected Native Vegetation | Biome Level Unprotected 1km RLRL Native Vegetation (ha) | Percent of 1km RLRL Area Covered by Unprotected Native Vegetation |
|---|---|---|---|---|---|
| Amazon | 373,200,745 | 193,130,032 | 51.75% | 132,136,531 | 35.41% |
| Atlantic Forest | 41,195,936 | 3,337,316 | 8.10% | 11,819,994 | 28.69% |
| Caatinga | 44,506,033 | 3,933,776 | 8.84% | 26,539,462 | 59.63% |
| Cerrado | 128,064,733 | 14,963,654 | 11.68% | 63,337,419 | 49.46% |
| Pampas | 7,360,547 | 249,241 | 3.39% | 3,908,509 | 53.10% |
| Pantanal | 12,177,264 | 475,935 | 3.91% | 9,428,460 | 77.43% |
| National Total | 606,505,258 | 216,089,953 | 35.63% | 247,170,374 | 40.75% |

Protection Status of Native Vegetated 1km RLRL Area as Percent of Overall 1km RLRL Area per Biome (RLRL Area as Denominator). * Unprotected areas are outside of Legally Protected Areas (LPAs), and therefore are located predominantly on private lands, government owned lands that are not conservation focused, or lands of unknown tenure status. **Protected areas are inside of Legally Protected Areas (LPAs) which include all of Brazil's protected areas in the National System of Conservation Units, titled Indigenous Territories and Maroon Community Lands

**Table S40.**

| Biomes | Total 1km RLRL Area per Biome (ha) | Biome Level Protected 1km RLRL Abiotic Areas (ha) | Percent of 1km RLRL Area Covered by Protected Abiotic Areas | Biome Level Unprotected 1km RLRL Abiotic Areas (ha) | Percent of 1km RLRL Area Covered by Unprotected Abiotic Areas |
|---|---|---|---|---|---|
| Amazon | 373,200,745 | 8,485,189 | 2.27% | 11,031,628 | 2.96% |
| Atlantic Forest | 41,195,936 | 269,000 | 0.65% | 1,118,056 | 2.71% |
| Caatinga | 44,506,033 | 207,709 | 0.47% | 211,887 | 0.48% |
| Cerrado | 128,064,733 | 289,545 | 0.23% | 953,220 | 0.74% |
| Pampas | 7,360,547 | 18,357 | 0.25% | 356,299 | 4.84% |
| Pantanal | 12,177,264 | 79,876 | 0.66% | 488,919 | 4.02% |
| National Total | 606,505,258 | 9,349,676 | 1.54% | 14,160,008 | 2.33% |

Protection Status of Abiotic Areas 1km RLRL Area as Percent of Overall 1km RLRL Area per Biome (RLRL Area as Denominator). *Unprotected areas are outside of Legally Protected Areas (LPAs), and therefore are located predominantly on private lands, government owned lands that are not conservation focused, or lands of unknown tenure status. **Protected areas are inside of Legally Protected Areas (LPAs) which include all of Brazil's protected areas in the National System of Conservation Units, titled Indigenous Territories and Maroon Community Lands

**Table S41.**

| Biomes | Total 1km RLRL Area per Biome (ha) | Biome Level Protected 1km RLRL Anthropic Areas (ha) | Percent of 1km RLRL Area Covered by Protected Anthropic Areas | Biome Level Unprotected 1km RLRL Anthropic Areas (ha) | Percent of 1km RLRL Area Covered by Unprotected Anthropic Areas |
|---|---|---|---|---|---|
| Amazon | 373,200,745 | 1,334,815 | 0.36% | 26,748,981 | 7.17% |
| Atlantic Forest | 41,195,936 | 1,057,824 | 2.57% | 23,458,339 | 56.94% |
| Caatinga | 44,506,033 | 779,447 | 1.75% | 12,669,631 | 28.47% |
| Cerrado | 128,064,733 | 1,404,928 | 1.10% | 47,043,748 | 36.73% |
| Pampas | 7,360,547 | 27,787 | 0.38% | 2,786,131 | 37.85% |
| Pantanal | 12,177,264 | 10,752 | 0.09% | 1,661,860 | 13.65% |
| National Total | 606,505,258 | 4,615,553 | 0.76% | 114,368,690 | 18.86% |

Protection Status of Anthropic Areas 1km RLRL Area as Percent of Overall 1km RLRL Area per Biome (RLRL Area as Denominator). *Unprotected areas are outside of Legally Protected Areas (LPAs), and therefore are located predominantly on private lands, government owned lands that are not conservation focused, or lands of unknown tenure status. **Protected areas are inside of Legally Protected Areas (LPAs) which include all of Brazil’s protected areas in the National System of Conservation Units, titled Indigenous Territories and Maroon Community Lands

**Table S42.**

| Biomes | Total 5km RLRL Area per Biome (ha) | Biome Level Protected 5km RLRL Native Vegetation (ha) | Percent of 5km RLRL Area Covered by Protected Native Vegetation | Biome Level Unprotected 5km RLRL Native Vegetation (ha) | Percent of 5km RLRL Area Covered by Unprotected Native Vegetation |
|---|---|---|---|---|---|
| Amazon | 287,589,987 | 179,536,338 | 62.43% | 87,952,681 | 30.58% |
| Atlantic Forest | 2,552,609 | 520,155 | 20.38% | 621,614 | 24.35% |
| Caatinga | 5,032,180 | 983,579 | 19.55% | 2,726,417 | 54.18% |
| Cerrado | 31,072,937 | 7,557,287 | 24.32% | 14,612,317 | 47.03% |
| Pampas | 211,301 | 13,899 | 6.58% | 90,002 | 42.59% |
| Pantanal | 6,290,184 | 330,611 | 5.26% | 4,909,628 | 78.05% |
| National Total | 332,749,198 | 188,941,868 | 56.78% | 110,912,659 | 33.33% |

Protection Status of Native Vegetated 5km RLRL Area as Percent of Overall 5km RLRL Area per Biome (RLRL Area as Denominator). *Unprotected areas are outside of Legally Protected Areas (LPAs), and therefore are located predominantly on private lands, government owned lands that are not conservation focused, or lands of unknown tenure status. **Protected areas are inside of Legally Protected Areas (LPAs) which include all of Brazil's protected areas in the National System of Conservation Units, titled Indigenous Territories and Maroon Community Lands.

**Table S43.**

| Biomes | Total 5km RLRL Area per Biome (ha) | Biome Level Protected 5km RLRL Abiotic Areas (ha) | Percent of 5km RLRL Area Covered by Protected Abiotic Areas | Biome Level Unprotected 5km RLRL Abiotic Areas (ha) | Percent of 5km RLRL Area Covered by Unprotected Abiotic Areas |
|---|---|---|---|---|---|
| Amazon | 287,589,987 | 6,631,745 | 2.31% | 7,634,025 | 2.65% |
| Atlantic Forest | 2,552,609 | 35,350 | 1.38% | 184,192 | 7.22% |
| Caatinga | 5,032,180 | 127,741 | 2.54% | 6,828 | 0.14% |
| Cerrado | 31,072,937 | 117,615 | 0.38% | 145,604 | 0.47% |
| Pampas | 211,301 | 1,247 | 0.59% | 50,385 | 23.85% |
| Pantanal | 6,290,184 | 73,478 | 1.17% | 341,594 | 5.43% |
| National Total | 332,749,198 | 6,987,177 | 2.10% | 8,362,627 | 2.51% |

Protection Status of Abiotic Areas 5km RLRL Area as Percent of Overall 5km RLRL Area per Biome (RLRL Area as Denominator). *Unprotected areas are outside of Legally Protected Areas (LPAs), and therefore are located predominantly on private lands, government owned lands that are not conservation focused, or lands of unknown tenure status. **Protected areas are inside of Legally Protected Areas (LPAs) which include all of Brazil's protected areas in the National System of Conservation Units, titled Indigenous Territories and Maroon Community Lands.

**Table S44.**

| Biomes | Total 5km RLRL Area per Biome (ha) | Biome Level Protected 5km RLRL Anthropic Areas (ha) | Percent of 5km RLRL Area Covered by Protected Anthropic Areas | Biome Level Unprotected 5km RLRL Anthropic Areas (ha) | Percent of 5km RLRL Area Covered by Unprotected Anthropic Areas |
|---|---|---|---|---|---|
| Amazon | 287,589,987 | 554,229 | 0.19% | 5,062,222 | 1.76% |
| Atlantic Forest | 2,552,609 | 35,525 | 1.39% | 1,146,730 | 44.92% |
| Caatinga | 5,032,180 | 165,327 | 3.29% | 1,005,451 | 19.98% |
| Cerrado | 31,072,937 | 264,549 | 0.85% | 8,361,262 | 26.91% |
| Pampas | 211,301 | 2,545 | 1.20% | 51,333 | 24.29% |
| Pantanal | 6,290,184 | 2,355 | 0.04% | 617,459 | 9.82% |
| National Total | 332,749,198 | 1,024,530 | 0.31% | 16,244,456 | 4.88% |

Protection Status of Anthropic Areas 5km RLRL Area as Percent of Overall 5km RLRL Area per Biome (RLRL Area as Denominator). *Unprotected areas are outside of Legally Protected Areas (LPAs), and therefore are located predominantly on private lands, government owned lands that are not conservation focused, or lands of unknown tenure status. **Protected areas are inside of Legally Protected Areas (LPAs) which include all of Brazil’s protected areas in the National System of Conservation Units, titled Indigenous Territories and Maroon Community Lands.

**Table S45.**

| Biomes | Total 1km RLRL Area per Biome (ha) | Biome Level Overlapping Unprotected PABCs & 1km RLRL Areas (ha) | Percent of 1km RLRL Area Designated as an Unprotected PABC |
|---|---|---|---|
| Amazon | 373,200,745 | 78,721,412 | 21.09% |
| Atlantic Forest | 41,195,936 | 6,442,430 | 15.64% |
| Caatinga | 44,506,033 | 13,702,354 | 30.79% |
| Cerrado | 128,064,733 | 41,163,064 | 32.14% |
| Pampas | 7,360,547 | 1,715,872 | 23.31% |
| Pantanal | 12,177,264 | 5,709,883 | 46.89% |
| National Total | 606,505,258 | 147,455,016 | 24.31% |

1km RLRL Areas and Unprotected Priority Areas for Biodiversity Conservation (PABCs) (RLRL Area as Denominator). *Unprotected areas are outside of Legally Protected Areas (LPAs), and therefore are located predominantly on private lands, government owned lands that are not conservation focused, or lands of unknown tenure status.

**Table S46.**

| Biomes | Total 5km RLRL Area per Biome (ha) | Biome Level Overlapping Unprotected PABCs & 5km RLRL Areas (ha) | Percent of 5km RLRL Area Designated as an Unprotected PABC |
|---|---|---|---|
| Amazon | 287,589,987 | 47,501,326 | 16.52% |
| Atlantic Forest | 2,552,609 | 432,767 | 16.95% |
| Caatinga | 5,032,180 | 1,451,545 | 28.85% |
| Cerrado | 31,072,937 | 9,327,073 | 30.02% |
| Pampas | 211,301 | 37,966 | 17.97% |
| Pantanal | 6,290,184 | 2,778,177 | 44.17% |
| National Total | 332,749,198 | 61,528,854 | 18.49% |

5km RLRL Areas and Unprotected Priority Areas for Biodiversity Conservation (PABCs) (RLRL Area as Denominator). *Unprotected areas are outside of Legally Protected Areas (LPAs), and therefore are located predominantly on private lands, government owned lands that are not conservation focused, or lands of unknown tenure status.

**Table S47.**

| Biomes | Total 1km RLRL Area per Biome (ha) | Biome Level Native Vegetation in Overlapping Unprotected PABCs & 1km RLRLs (ha) | Percent of 1km RLRL Area Covered by Native Vegetation in Overlapping Unprotected PABCs |
|---|---|---|---|
| Amazon | 373,200,745 | 62,226,990 | 16.67% |
| Atlantic Forest | 41,195,936 | 2,647,126 | 6.43% |
| Caatinga | 44,506,033 | 9,707,447 | 21.81% |
| Cerrado | 128,064,733 | 26,783,483 | 20.91% |
| Pampas | 7,360,547 | 1,193,432 | 16.21% |
| Pantanal | 12,177,264 | 4,752,044 | 39.02% |
| National Total | 606,505,258 | 107,310,522 | 17.69% |

1km RLRL Areas, Unprotected Priority Areas for Biodiversity Conservation (PABCs) and Remaining Native Vegetation Cover (RLRL Area as Denominator). *Unprotected areas are outside of Legally Protected Areas (LPAs), and therefore are located predominantly on private lands, government owned lands that are not conservation focused, or lands of unknown tenure status.

**Table S48.**

| Biomes | Total 1km RLRL Area per Biome (ha) | Biome Level Abiotic Areas in Overlapping Unprotected PABCs & 1km RLRLs (ha) | Percent of 1km RLRL Area Covered by Abiotic Areas in Overlapping Unprotected PABCs |
|---|---|---|---|
| Amazon | 373,200,745 | 5,629,168 | 1.51% |
| Atlantic Forest | 41,195,936 | 124,770 | 0.30% |
| Caatinga | 44,506,033 | 115,013 | 0.26% |
| Cerrado | 128,064,733 | 475,425 | 0.37% |
| Pampas | 7,360,547 | 38,774 | 0.53% |
| Pantanal | 12,177,264 | 152,571 | 1.25% |
| National Total | 606,505,258 | 6,535,721 | 1.08% |

1km RLRL Areas, Unprotected Priority Areas for Biodiversity Conservation (PABCs) with Abiotic Landcover (RLRL Area as Denominator). *Unprotected areas are outside of Legally Protected Areas (LPAs), and therefore are located predominantly on private lands, government owned lands that are not conservation focused, or lands of unknown tenure status.

**Table S49.**

| Biomes | Total 1km RLRL Area per Biome (ha) | Biome Level Anthropic Areas in Overlapping Unprotected PABCs & 1km RLRLs (ha) | Percent of 1km RLRL Area Covered by Anthropic Areas in Overlapping Unprotected PABCs |
|---|---|---|---|
| Amazon | 373,200,745 | 10,784,801 | 2.89% |
| Atlantic Forest | 41,195,936 | 3,650,992 | 8.86% |
| Caatinga | 44,506,033 | 3,820,441 | 8.58% |
| Cerrado | 128,064,733 | 13,887,704 | 10.84% |
| Pampas | 7,360,547 | 481,028 | 6.54% |
| Pantanal | 12,177,264 | 791,473 | 6.50% |
| National Total | 606,505,258 | 33,416,440 | 5.51% |

1km RLRL Areas, Unprotected Priority Areas for Biodiversity Conservation (PABCs) with Anthropic Landcover (RLRL Area as Denominator). *Unprotected areas are outside of Legally Protected Areas (LPAs), and therefore are located predominantly on private lands, government owned lands that are not conservation focused, or lands of unknown tenure status.

**Table S50.**

| Biomes | Total 5km RLRL Area per Biome (ha) | Biome Level Native Vegetation in Overlapping Unprotected PABCs & 5km RLRL Areas (ha) | Percent of 5km RLRL Area Covered by Native Vegetation in Overlapping Unprotected PABCs |
|---|---|---|---|
| Amazon | 287,589,987 | 41,700,182 | 14.50% |
| Atlantic Forest | 2,552,609 | 160,628 | 6.29% |
| Caatinga | 5,032,180 | 1,113,756 | 22.13% |
| Cerrado | 31,072,937 | 6,747,348 | 21.71% |
| Pampas | 211,301 | 23,864 | 11.29% |
| Pantanal | 6,290,184 | 2,377,982 | 37.80% |
| National Total | 332,749,198 | 52,123,760 | 15.66% |

5km RLRL Areas, Unprotected Priority Areas for Biodiversity Conservation (PABCs) and Remaining Native Vegetation Cover (RLRL Area as Denominator). *Unprotected areas are outside of Legally Protected Areas (LPAs), and therefore are located predominantly on private lands, government owned lands that are not conservation focused, or lands of unknown tenure status.

**Table S51.**

| Biomes | Total 5km RLRL Area per Biome (ha) | Biome Level Abiotic Areas in Overlapping Unprotected PABCs & 5km RLRLs (ha) | Percent of 5km RLRL Area Covered by Abiotic Areas in Overlapping Unprotected PABCs |
|---|---|---|---|
| Amazon | 287,589,987 | 3,784,328 | 1.32% |
| Atlantic Forest | 2,552,609 | 18,632 | 0.73% |
| Caatinga | 5,032,180 | 2,963 | 0.06% |
| Cerrado | 31,072,937 | 86,469 | 0.28% |
| Pampas | 211,301 | 3,172 | 1.50% |
| Pantanal | 6,290,184 | 101,210 | 1.61% |
| National Total | 332,749,198 | 3,996,775 | 1.20% |

5km RLRL Areas, Unprotected Priority Areas for Biodiversity Conservation (PABCs) with Abiotic Area Landcover (RLRL Area as Denominator). *Unprotected areas are outside of Legally Protected Areas (LPAs), and therefore are located predominantly on private lands, government owned lands that are not conservation focused, or lands of unknown tenure status.

**Table S52.**

| Biomes | Total 5km RLRL Area per Biome (ha) | Biome Level Anthropic Areas in Overlapping Unprotected PABCs & 5km RLRLs (ha) | Percent of 5km RLRL Area Covered by Anthropic Areas in Overlapping Unprotected PABCs |
|---|---|---|---|
| Amazon | 287,589,987 | 1,975,653 | 0.69% |
| Atlantic Forest | 2,552,609 | 252,376 | 9.89% |
| Caatinga | 5,032,180 | 330,184 | 6.56% |
| Cerrado | 31,072,937 | 2,490,483 | 8.01% |
| Pampas | 211,301 | 10,620 | 5.03% |
| Pantanal | 6,290,184 | 292,918 | 4.66% |
| National Total | 332,749,198 | 5,352,233 | 1.61% |

5km RLRL Areas, Unprotected Priority Areas for Biodiversity Conservation (PABCs) with Anthropic Area Landcover (RLRL Area as Denominator). *Unprotected areas are outside of Legally Protected Areas (LPAs), and therefore are located predominantly on private lands, government owned lands that are not conservation focused, or lands of unknown tenure status.

**Table S53.**

| Biomes | Biome Level Remaining Native Vegetation (ha) | Biome Level 1km RLRL Native Vegetation (ha) | Percent of Remaining Native Vegetation found in 1km RLRL Areas | Biome Level 5km RLRL Native Vegetation (ha) | Percent of Remaining Native Vegetation found in 5km RLRL Areas |
|---|---|---|---|---|---|
| Amazon | 349,502,078 | 325,266,562 | 93.07% | 267,489,018 | 76.53% |
| Atlantic Forest | 34,583,015 | 15,157,309 | 43.83% | 1,141,769 | 3.30% |
| Caatinga | 51,272,144 | 30,473,237 | 59.43% | 3,709,996 | 7.24% |
| Cerrado | 111,835,766 | 78,301,073 | 70.01% | 22,169,604 | 19.82% |
| Pampas | 9,379,999 | 4,157,750 | 44.33% | 103,901 | 1.11% |
| Pantanal | 11,799,943 | 9,904,395 | 83.94% | 5,240,239 | 44.41% |
| National Total | 568,372,944 | 463,260,327 | 81.51% | 299,854,527 | 52.76% |

Biome’s Remaining Native Vegetation Cover and Percent Found in RLRL Areas (Remaining Native Vegetation as Denominator).

**Table S54.**

| Biomes | Biome Level Remaining Native Vegetation (ha) | Biome Level Protected 1km RLRL Native Vegetation (ha) | Percent of Remaining Native Vegetation found in a Protected 1km RLRL Area | Biome's Total Unprotected 1km RLRL Native Vegetation (ha) | Percent of Remaining Native Vegetation found in an Unprotected 1km RLRL Area |
|---|---|---|---|---|---|
| Amazon | 349,502,078 | 193,130,032 | 55.26% | 132,136,531 | 37.81% |
| Atlantic Forest | 34,583,015 | 3,337,316 | 9.65% | 11,819,994 | 34.18% |
| Caatinga | 51,272,144 | 3,933,776 | 7.67% | 26,539,462 | 51.76% |
| Cerrado | 111,835,766 | 14,963,654 | 13.38% | 63,337,419 | 56.63% |
| Pampas | 9,379,999 | 249,241 | 2.66% | 3,908,509 | 41.67% |
| Pantanal | 11,799,943 | 475,935 | 4.03% | 9,428,460 | 79.90% |
| National Total | 568,372,944 | 216,089,953 | 38.02% | 247,170,374 | 43.49% |

Biome's Remaining Native Vegetation Cover and Proportion Found as either Protected or Unprotected in 1km RLRL Areas (Remaining Native Vegetation as Denominator). *Unprotected areas are outside of Legally Protected Areas (LPAs), and therefore are located predominantly on private lands, government owned lands that are not conservation focused, or lands of unknown tenure status. **Protected areas are inside of Legally Protected Areas (LPAs) which include all of Brazil's protected areas in the National System of Conservation Units, titled Indigenous Territories and Maroon Community Lands.

**Table S55.**

| Biomes | Biome Level Remaining Native Vegetation (ha) | Biome Level Protected 5km RLRL Native Vegetation (ha) | Percent of Remaining Native Vegetation found in a Protected 5km RLRL Area | Biome's Total Unprotected 5km RLRL Native Vegetation (ha) | Percent of Remaining Native Vegetation found in an Unprotected 5km RLRL Area |
|---|---|---|---|---|---|
| Amazon | 349,502,078 | 179,536,338 | 51.37% | 87,952,681 | 25.17% |
| Atlantic Forest | 34,583,015 | 520,155 | 1.50% | 621,614 | 1.80% |
| Caatinga | 51,272,144 | 983,579 | 1.92% | 2,726,417 | 5.32% |
| Cerrado | 111,835,766 | 7,557,287 | 6.76% | 14,612,317 | 13.07% |
| Pampas | 9,379,999 | 13,899 | 0.15% | 90,002 | 0.96% |
| Pantanal | 11,799,943 | 330,611 | 2.80% | 4,909,628 | 41.61% |
| National Total | 568,372,944 | 188,941,868 | 33.24% | 110,912,659 | 19.51% |

Biome’s Remaining Native Vegetation Cover and Proportion Found as either Protected or Unprotected in 5km RLRL Areas (Remaining Native Vegetation as Denominator). *Unprotected areas are outside of Legally Protected Areas (LPAs), and therefore are located predominantly on private lands, government owned lands that are not conservation focused, or lands of unknown tenure status. **Protected areas are inside of Legally Protected Areas (LPAs) which include all of Brazil’s protected areas in the National System of Conservation Units, titled Indigenous Territories and Maroon Community Lands.

**Table S56.**

| Biomes | Biome Level Remaining Native Vegetation (ha) | Biome Level Native Vegetation in PABCs (ha) | Percent of Remaining Native Vegetation found in Unprotected PABCs |
|---|---|---|---|
| Amazon | 349,502,078 | 71,690,495 | 20.51% |
| Atlantic Forest | 34,583,015 | 5,794,379 | 16.75% |
| Caatinga | 51,272,144 | 16,545,243 | 32.27% |
| Cerrado | 111,835,766 | 38,420,748 | 34.35% |
| Pampas | 9,379,999 | 2,556,202 | 27.25% |
| Pantanal | 11,799,943 | 5,692,518 | 48.24% |
| National Total | 568,372,944 | 140,699,586 | 24.75% |

Biome's Remaining Native Vegetation Cover and Proportion Found in Unprotected PABCs (Remaining Native Vegetation as Denominator). *Unprotected areas are outside of Legally Protected Areas (LPAs), and therefore are located predominantly on private lands, government owned lands that are not conservation focused, or lands of unknown tenure status.

**Table S57.**

| Biomes | Biome Level Remaining Native Vegetation (ha) | Biome Level Native Vegetation in Overlapping Unprotected PABCs & 1km RLRLs (ha) | Percent of Remaining Native Vegetation found in in Overlapping Unprotected PABCs & 1km RLRL Areas | Biome Level Native Vegetation in Overlapping Unprotected PABCs & 5km RLRL Areas (ha) | Percent of Remaining Native Vegetation found in Overlapping Unprotected PABCs & 5km RLRL Areas |
|---|---|---|---|---|---|
| Amazon | 349,502,078 | 62,226,990 | 17.80% | 41,700,182 | 11.93% |
| Atlantic Forest | 34,583,015 | 2,647,126 | 7.65% | 160,628 | 0.46% |
| Caatinga | 51,272,144 | 9,707,447 | 18.93% | 1,113,756 | 2.17% |
| Cerrado | 111,835,766 | 26,783,483 | 23.95% | 6,747,348 | 6.03% |
| Pampas | 9,379,999 | 1,193,432 | 12.72% | 23,864 | 0.25% |
| Pantanal | 11,799,943 | 4,752,044 | 40.27% | 2,377,982 | 20.15% |
| National Total | 568,372,944 | 107,310,522 | 18.88% | 52,123,760 | 9.17% |

Biome's Remaining Native Vegetation Cover and Proportion Found in Unprotected PABCs & RLRL Areas (Remaining Native Vegetation as Denominator). *Unprotected areas are outside of Legally Protected Areas (LPAs), and therefore are located predominantly on private lands, government owned lands that are not conservation focused, or lands of unknown tenure status.

**Table S58.**

| Biomes | Biome Level Remaining Native Vegetation (ha) | Biome Level Protected Native Vegetation (ha) | Percent of Remaining Biome Level Native Vegetation that is Protected | Biome Level Unprotected Native Vegetation (ha) | Percent of Remaining Biome Level Native Vegetation that is Unprotected) |
|---|---|---|---|---|---|
| Amazon | 349,502,078 | 196,912,767 | 56.34% | 152,589,310 | 43.66% |
| Atlantic Forest | 34,583,015 | 5,817,039 | 16.82% | 28,765,976 | 83.18% |
| Caatinga | 51,272,144 | 5,733,752 | 11.18% | 45,538,392 | 88.82% |
| Cerrado | 111,835,766 | 18,893,604 | 16.89% | 92,942,163 | 83.11% |
| Pampas | 9,379,999 | 437,431 | 4.66% | 8,942,568 | 95.34% |
| Pantanal | 11,799,943 | 541,933 | 4.59% | 11,258,010 | 95.41% |
| National Total | 568,372,944 | 228,336,526 | 40.17% | 340,036,418 | 59.83% |

Biome's Remaining Protected and Unprotected Native Vegetation Cover (Remaining Native Vegetation as Denominator). *Unprotected areas are outside of Legally Protected Areas (LPAs), and therefore are located predominantly on private lands, government owned lands that are not conservation focused, or lands of unknown tenure status. Protected areas are inside of Legally Protected Areas (LPAs) which include all of Brazil's protected areas in the National System of Conservation Units, titled Indigenous Territories and Maroon Community Lands.*

**Table S59.**

| Biomes | Biome Area (ha) | Biome Level Protected Area*** Coverage (ha) | Percent of Biome Area Protected by an LPA *** | Surplus or Deficit for Brazil's NBSAP Target 11 and CDB Aichi Target 11 (30% Protected for Amazon, 17% for all other Biomes) |
|---|---|---|---|---|
| Amazon | 421,386,509 | 208,401,863 | 49.46% | Surplus 19.46% |
| Atlantic Forest | 110,596,318 | 9,813,436 | 8.87% | ***Deficit*** *(8.13%)* |
| Caatinga | 83,594,452 | 7,385,532 | 8.83% | ***Deficit*** *(8.17%)* |
| Cerrado | 202,937,475 | 22,442,904 | 11.06% | ***Deficit*** *(5.94%)* |
| Pampas | 16,432,052 | 542,339 | 3.30% | ***Deficit*** *(13.70%)* |
| Pantanal | 15,010,803 | 639,249 | 4.26% | ***Deficit*** *(12.74%)* |
| **National Total** | 849,957,610 | 249,225,324 | 29.32%* | Surplus 5.88% |
| **National Total (without the Amazon)** | 428,571,101 | 40,823,460,67 | 9.53%** | **Deficit** *(7.47%)* |

Biome Protected Area Status Compared to Brazil's NBSAP Target 11 and Aichi Target 11 (Biome Area as Denominator).******For the National Total, 23.45% of national area should be legally protected based on proportional area minimums for each biome.* ****** *For the National Level excluding the Amazon, 17% of the combined Area of Brazil's 5 other Biomes should be legally protected based on proportional area minimums for each biome. *** Protected Areas (Legally Protected Areas – LPAs) includes Strictly Protected and Sustainable Use Protected Areas in Brazil's National Conservation Unit System, as well as fully titled Indigenous Territories and Maroon Community Lands.*

**Table S60.**

| Biomes | Biome Area (ha) | Biome Level Protected *** Native Vegetation (ha) | Percent of Biome Area Covered by Protected *** Native Vegetation | Surplus or Deficit for Brazil's NBSAP Target 11 and CDB Aichi Target 11 (30% Protected for Amazon, 17% for all other Biomes) |
|---|---|---|---|---|
| Amazon | 421,386,509 | 196,912,767 | 46.73% | Surplus 16.73% |
| Atlantic Forest | 110,596,318 | 5,817,039 | 5.26% | ***Deficit*** *(11.74%)* |
| Caatinga | 83,594,452 | 5,733,752 | 6.86% | ***Deficit*** *(10.14%)* |
| Cerrado | 202,937,475 | 18,893,604 | 9.31% | ***Deficit*** *(7.69%)* |
| Pampas | 16,432,052 | 437,431 | 2.66% | ***Deficit*** *(14.34%)* |
| Pantanal | 15,010,803 | 541,933 | 3.61% | ***Deficit*** *(13.39%)* |
| **National Total** | 849,957,610 | 228,336,526 | 26.86%***** | Surplus 3.42% |
| **National Total (without the Amazon)** | **428,571,101** | 31,423,759 | 7.33%****** | ***Deficit*** *(9.67%)* |

Biome Protected Area Native Vegetation Status Compared to Brazil's NBSAP Target 11 and Aichi Target 11 (Biome Area as Denominator). ***For the National Total, 23.45% of national area should be legally protected based on proportional area minimums for each biome. ** For the National Level excluding the Amazon, 17% of the combined Area of Brazil's 5 other Biomes should be legally protected based on proportional area minimums for each biome. *** Protected Areas (Legally Protected Areas – LPAs) includes Strictly Protected and Sustainable Use Protected Areas in Brazil's National Conservation Unit System, as well as fully titled Indigenous Territories and Maroon Community Lands.***

**Table S61.**

| Biomes | Intercept (Alpha) | SE (Alpha) | z value (Alpha) | Coefficient (Beta) | SE (Beta) | z value (Beta) | McFadden's pseudo $R^2$ | Hosmer-Lemeshow GOF test |
|---|---|---|---|---|---|---|---|---|
| Amazon | 0.33007*** | 0.00058 | 565.1 | 0.25964*** | 0.00016 | 1668.7 | 0.27139 | <2.2e-16 |
| Atlantic Forest | -0.8881*** | 0.00067 | -1324.0 | 0.19186*** | 0.00038 | 507.0 | 0.01201 | <2.2e-16 |
| Caatinga | 0.32505*** | 0.00080 | 405.9 | 0.21835*** | 0.00039 | 552.4 | 0.02161 | <2.2e-16 |
| Cerrado | -0.1486*** | 0.00048 | -311.3 | 0.14148*** | 0.00014 | 1020.7 | 0.03186 | <2.2e-16 |
| Pampas | 0.47058*** | 0.00183 | 257.0 | -0.0089*** | 0.00116 | -7.7 | 0.00002 | <2.2e-16 |
| Pantanal | 1.02158*** | 0.00239 | 427.3 | 0.11671*** | 0.00044 | 264.7 | 0.09479 | <2.2e-16 |
| P-values: | 0.0001 *** / 0.001 ** / 0.01 * / 0.05 '' | | | | | | | |

Coefficient table for Principal Logistic Regression Model (***Model 1*** – See Materials and Methods: Statistical Analysis)

*The resulting logistic regression intercepts in this model represents the log-odds of encountering native vegetation at the side of transportation infrastructure. The resulting logistic regression coefficients in this model represent the expected change in the log-odds of encountering native vegetation per kilometer increase in distance away from transportation infrastructure.*

**Table S62.**

| Biomes | Intercept (Alpha) | SE (Alpha) | z value (Alpha) | Coefficient (Beta) | SE (Beta) | z value (Beta) | McFadden's pseudo $R^2$ | Hosmer-Lemeshow GOF test |
|---|---|---|---|---|---|---|---|---|
| Amazon | 0.20095*** | 0.00061 | 330.8 | 0.20798*** | 0.00016 | 1277.1 | 0.16925 | <2.2e-16 |
| Atlantic Forest | -0.9441*** | 0.00071 | -1321.3 | 0.12148*** | 0.00042 | 288.1 | 0.00422 | <2.2e-16 |
| Caatinga | 0.26497*** | 0.00083 | 318.3 | 0.21718*** | 0.00043 | 509.6 | 0.01921 | <2.2e-16 |
| Cerrado | -0.2055*** | 0.00050 | -409.4 | 0.10944*** | 0.00015 | 730.5 | 0.01564 | <2.2e-16 |
| Pampas | 0.45278*** | 0.00186 | 244.0 | -0.0269*** | 0.00118 | -22.8 | 0.00016 | <2.2e-16 |
| Pantanal | 0.99941*** | 0.00241 | 414.3 | 0.11403*** | 0.00045 | 255.1 | 0.04266 | <2.2e-16 |
| P-values: | 0.0001 *** / 0.001 ** / 0.01 * / 0.05 " | | | | | | | |

Coefficient table for Unprotected Biome Areas Logistic Regression Model (***Model 2*** – See Materials and Methods: Statistical Analysis)

*The resulting logistic regression intercepts in this model represents the log-odds of encountering native vegetation at the side of transportation infrastructure only in **unprotected** parts of biomes. The resulting logistic regression coefficients in this model represent the expected change in the log-odds of encountering native vegetation per kilometer increase in distance away from transportation infrastructure only in **unprotected** parts of biomes.*

**Table S63.**

| Biomes | Intercept (Alpha) | SE (Alpha) | z value (Alpha) | Coefficient (Beta) | SE (Beta) | z value (Beta) | McFadden's pseudo R2 | Hosmer-Lemeshow GOF test |
|---|---|---|---|---|---|---|---|---|
| Amazon | 2.47382*** | 0.00294 | 842.0 | 0.22219*** | 0.00052 | 424.6 | 0.22656 | <2.2e-16 |
| Atlantic Forest | -0.0673*** | 0.00249 | -27.0 | 0.59231*** | 0.00174 | 340.0 | 0.09890 | <2.2e-16 |
| Caatinga | 1.43545*** | 0.00349 | 411.0 | 0.09714*** | 0.00108 | 90.0 | 0.02161 | <2.2e-16 |
| Cerrado | 0.97158*** | 0.00218 | 445.9 | 0.31189*** | 0.00080 | 389.2 | 0.12334 | <2.2e-16 |
| Pampas | 1.70634*** | 0.01558 | 109.6 | 0.24687*** | 0.00932 | 26.5 | 0.01383 | <2.2e-16 |
| Pantanal | 2.88905*** | 0.03127 | 92.4 | 0.17697*** | 0.00626 | 28.3 | 0.09825 | <2.2e-16 |
| P-values: | 0.0001 *** / 0.001 ** / 0.01 * / 0.05 " | | | | | | | |

Coefficient table for Protected Biome Areas Logistic Regression Model (***Model 3*** – See Materials and Methods: Statistical Analysis)

*The resulting logistic regression intercepts in this model represents the log-odds of encountering native vegetation at the side of transportation infrastructure only in* ***protected*** *parts of biomes. The resulting logistic regression coefficients in this model represent the expected change in the log-odds of encountering native vegetation per kilometer increase in distance away from transportation infrastructure only in* ***protected*** *parts of biomes.*

**Table S64.**

| Biomes | Intercept (Alpha) | SE (Alpha) | z value (Alpha) | Coefficient (Beta) | SE (Beta) | z value (Beta) | McFadden's pseudo R2 | Hosmer-Lemeshow GOF test |
|---|---|---|---|---|---|---|---|---|
| Amazon | 0.27138*** | 0.00062 | 440.7 | 0.25096*** | 0.00016 | 1547.6 | 0.26462 | <2.2e-16 |
| Atlantic Forest | -0.8995*** | 0.00070 | -1284.3 | 0.18783*** | 0.00039 | 478.4 | 0.01153 | <2.2e-16 |
| Caatinga | 0.30751*** | 0.00082 | 374.0 | 0.22293*** | 0.00041 | 549.4 | 0.02257 | <2.2e-16 |
| Cerrado | -0.1302*** | 0.00050 | -261.4 | 0.13546*** | 0.00015 | 932.8 | 0.02795 | <2.2e-16 |
| Pampas | 0.51496*** | 0.00197 | 262.1 | -0.0409*** | 0.00124 | -33.0 | 0.00036 | <2.2e-16 |
| Pantanal | 1.01437*** | 0.00250 | 405.8 | 0.10837*** | 0.00046 | 233.5 | 0.03867 | <2.2e-16 |
| P-values: | 0.0001 *** / 0.001 ** / 0.01 * / 0.05 " | | | | | | | |

Coefficient table for Road only Logistic Regression Model (***Model 4*** – See Materials and Methods: Statistical Analysis)

*The resulting logistic regression intercepts in this model represents the log-odds of encountering native vegetation at the side of **roads** only in parts of biomes where **roads** are the closest infrastructure type. The resulting logistic regression coefficients in this model represent the expected change in the log-odds of encountering native vegetation per kilometer increase in distance away from **roads** only in parts of biomes where **roads** are the closest infrastructure type.*

**Table S65.**

| Biomes | Intercept (Alpha) | SE (Alpha) | z value (Alpha) | Coefficient (Beta) | SE (Beta) | z value (Beta) | McFadden's pseudo R2 | Hosmer-Lemeshow GOF test |
|---|---|---|---|---|---|---|---|---|
| Amazon | 0.54107*** | 0.00685 | 78.95 | 0.06044*** | 0.00083 | 73.22 | 0.03369 | <2.2e-16 |
| Atlantic Forest | -0.8653*** | 0.00460 | -188.02 | 0.20820*** | 0.00328 | 63.54 | 0.00977 | <2.2e-16 |
| Caatinga | 0.46961*** | 0.00535 | 87.78 | 0.05409*** | 0.00278 | 19.45 | 0.02257 | <2.2e-16 |
| Cerrado | -0.1302*** | 0.00050 | -261.40 | 0.13546*** | 0.00015 | 932.80 | 0.02795 | <2.2e-16 |
| Pampas | 0.52613*** | 0.00970 | 54.23 | 0.02841*** | 0.00677 | 4.20 | 0.00036 | <2.2e-16 |
| Pantanal | 1.40622*** | 0.03733 | 37.67 | 0.37074*** | 0.01337 | 27.73 | 0.15162 | <2.2e-16 |
| P-values: | 0.0001 *** / 0.001 ** / 0.01 * / 0.05 " | | | | | | | |

Coefficient table for Railroad only Logistic Regression Model (***Model 5*** – See Materials and Methods: Statistical Analysis)

*The resulting logistic regression intercepts in this model represents the log-odds of encountering native vegetation at the side of* ***railroads*** *only in parts of biomes where* ***railroads*** *are the closest infrastructure type. The resulting logistic regression coefficients in this model represent the expected change in the log-odds of encountering native vegetation per kilometer increase in distance away from* ***railroads*** *only in parts of biomes where* ***railroads*** *are the closest infrastructure type.*

**Table S66.**

| Biomes | Intercept (Alpha) | SE (Alpha) | z value (Alpha) | Coefficient (Beta) | SE (Beta) | z value (Beta) | McFadden's pseudo R2 | Hosmer-Lemeshow GOF test |
|---|---|---|---|---|---|---|---|---|
| Amazon | 0.76149*** | 0.00200 | 380.60 | 0.35999*** | 0.00066 | 542.30 | 0.31097 | <2.2e-16 |
| Atlantic Forest | -0.7217*** | 0.00274 | -263.30 | 0.27035*** | 0.00167 | 162.30 | 0.02375 | <2.2e-16 |
| Caatinga | 0.80759*** | 0.00490 | 164.79 | 0.17194*** | 0.00243 | 70.67 | 0.01275 | <2.2e-16 |
| Cerrado | -0.2368*** | 0.00195 | -121.50 | 0.22477*** | 0.00059 | 377.90 | 0.09838 | <2.2e-16 |
| Pampas | 0.03404*** | 0.00615 | 5.54 | 0.26808*** | 0.00397 | 67.61 | 0.01751 | <2.2e-16 |
| Pantanal | 1.11918*** | 0.00856 | 130.80 | 0.15883*** | 0.00148 | 107.30 | 0.09479 | <2.2e-16 |
| P-values: | 0.0001 *** / 0.001 ** / 0.01 * / 0.05 '' | | | | | | | |

Coefficient table for Pathway only Logistic Regression Model (***Model 6*** – See Materials and Methods: Statistical Analysis)

*The resulting logistic regression intercepts in this model represents the log-odds of encountering native vegetation at the side of* ***pathways*** *only in parts of biomes where* ***pathways*** *are the closest infrastructure type. The resulting logistic regression coefficients in this model represent the expected change in the log-odds of encountering native vegetation per kilometer increase in distance away from* ***pathways*** *only in parts of biomes where* ***pathways*** *are the closest infrastructure type.*

**Table S67.**

| Biomes | (A) Initial Probability Biome-Wide Models | (B) Initial Probability Protected Areas Models | (C) Initial Probability Unprotected Areas Models | Difference in Initial Probability between (B) and (A) | Difference in Initial Probability between (B) and (C) | Difference in Initial Probability between (A) and (C) |
|---|---|---|---|---|---|---|
| Amazon | 58.18% | 92.23% | 55.01% | 34.05% | 37.22% | 3.17% |
| Atlantic Forest | 29.15% | 48.32% | 28.01% | 19.17% | 20.31% | 1.14% |
| Caatinga | 58.06% | 80.77% | 56.59% | 22.72% | 24.19% | 1.47% |
| Cerrado | 46.29% | 72.54% | 44.88% | 26.25% | 27.66% | 1.41% |
| Pampas | 61.55% | 84.64% | 61.13% | 23.08% | 23.51% | 0.42% |
| Pantanal | 73.53% | 94.73% | 73.09% | 21.20% | 21.64% | 0.43% |

Derived probabilities (and the differences between them) from the resulting estimated intercepts of the Biome-Wide Logit Models, the Protected Areas Logit Models, and the Unprotected Areas Logit Models. *Probabilities were derived from the intercept's inverse logit*.

**Table S68.**

| Biomes | Road Dominated Areas (ha) | Railway Dominated Areas (ha) | Pathway Dominated Areas (ha) |
|---|---|---|---|
| Amazon | 349,610,806 | 1,138,025 | 71,042,419 |
| Atlantic Forest | 102,774,569 | 2,274,006 | 6,058,125 |
| Caatinga | 79,571,119 | 1,695,700 | 2,536,188 |
| Cerrado | 187,428,063 | 3,023,850 | 13,153,825 |
| Pampas | 14,421,938 | 572,275 | 1,539,306 |
| Pantanal | 13,041,881 | 148,931 | 1,895,538 |

Biome area’s closest infrastructure class - Areas of each biome that are closest to one of the three transportation infrastructure classes.

**Table S69.**

| Biomes | Road Dominated Areas (ha) | Railway Dominated Areas (ha) | Pathway Dominated Areas (ha) |
|---|---|---|---|
| Amazon | 307,409,650 | 886,381 | 65,470,113 |
| Atlantic Forest | 38,990,113 | 624,888 | 2,104,269 |
| Caatinga | 42,619,975 | 764,294 | 1,319,269 |
| Cerrado | 118,648,806 | 1,641,763 | 8,271,938 |
| Pampas | 6,446,406 | 226,681 | 733,506 |
| Pantanal | 10,438,794 | 123,875 | 1,667,506 |

Biome’s 1 km RLRL Area’s closest infrastructure class – Proportion of 1km RLRL Areas of each biome that are closest to one of the three transportation infrastructure classes.

**Table S70.**

| Biomes | Road Dominated Areas (ha) | Railway Dominated Areas (ha) | Pathway Dominated Areas (ha) |
|---|---|---|---|
| Amazon | 233,504,150 | 478,838 | 54,674,906 |
| Atlantic Forest | 2,473,588 | 23,506 | 184,500 |
| Caatinga | 4,857,475 | 53,575 | 174,444 |
| Cerrado | 27,993,369 | 352,275 | 2,897,613 |
| Pampas | 149,275 | 3,175 | 56,388 |
| Pantanal | 5,123,619 | 71,494 | 1,094,063 |

Biome’s 5 km RLRL Area’s closest infrastructure class – Proportion of 5 km RLRL Areas of each biome that are closest to one of the three transportation infrastructure classes.

**Data S1.** Excel File – Resulting Data - Conservation Opportunities and Challenges in Brazil's Roadless and Railroad-Less Areas.xlsx

**Data S2.** Spatial Model Data – Spatial Data *(Shapefiles, Tiff, and GeoTiff files including All Modeled Results, Cleaned Pre-Processed Input Data, and Original Raw Input Data),* Model Scripts *(Python 2.7)*, ArcGIS Models, Supporting information, and Instructions in text files (*.txt*): https://doi.org/10.5281/zenodo.4555070

**Data S3.** Logistic Regression Analysis and Input Data – includes some Spatial Data *(Shapefiles, Tiff, GeoTiff [including Euclidean Distance Modeled Results] and tabular delimited text file [csv] files)* to thus transform the spatial data and use it as tabular data for input in the statistical analyses. Also includes the Model Scripts *(R scripts)* specifically written and executed in the R Studio environment, and Instructions in text files (*.txt*): https://doi.org/10.5281/zenodo.5617958

## REFERENCES AND NOTES


1. P. L. Ibisch, M. T. Hoffmann, S. Kreft, G. Pe'er, V. Kati, L. Biber-Freudenberger, D. A. DellaSala, M. M. Vale, P. R. Hobson, N. Selva, A global map of roadless areas and their conservation status. *Science* **354**, 1423–1427 (2016).

2. J. N. Popp, S. P. Boyle, Railway ecology: Underrepresented in science? *Basic Appl. Ecol.* **19**, 84–93 (2017).

3. R. A. P. Dornas, F. Z. Teixeira, G. Gonsioroski, R. A. A. Nóbrega, Strain by the train: Patterns of toad fatalities on a Brazilian Amazonian railroad. *Sci. Total Environ.* **660**, 493–500 (2019).

4. L. Borda-de-Água, R. Barrientos, P. Beja, H. M. Pereira, *Railway Ecology* (Springer Berlin Heidelberg, 2017).

5. G. K. Heilig, Neglected dimensions of global land-use change: Reflections and data. *Population Dev. Rev.* **20**, 831–859 (1994).

6. W. F. Laurance, G. R. Clements, S. Sloan, C. S. O'Connell, N. D. Mueller, M. Goosem, O. Venter, D. P. Edwards, B. Phalan, A. Balmford, R. Van Der Ree, I. B. Arrea, A global strategy for road building. *Nature* **513**, 229–232 (2014).

7. W. F. Laurance, in *Handbook of Road Ecology*, R. Van Der Ree, D. J. Smith, C. Grilo, Eds. (John Wiley & Sons Ltd., 2015; https://doi.org/10.1002/9781118568170.ch2), pp. 10–15.

8. C. P. Barber, M. A. Cochrane, C. M. Souza, W. F. Laurance, Roads, deforestation, and the mitigating effect of protected areas in the Amazon. *Biol. Conserv.* **177**, 203–209 (2014).

9. P. M. Fearnside, The roles and movements of actors in the deforestation of Brazilian Amazonia. *Ecol. Soc.* **13**, 23 (2008).

10. N. Selva, A. Switalski, S. Kreft, P. L. Ibisch, in *Handbook of Road Ecology*, R. Van Der Ree, D. J. Smith, C. Grilo, Eds. (John Wiley & Sons Ltd., 2015; https://doi.org/10.1002/9781118568170.ch3), pp. 16–26.

11. E. Dinerstein, C. Vynne, E. Sala, A. R. Joshi, S. Fernando, T. E. Lovejoy, J. Mayorga, D. Olson, G. P. Asner, J. E. M. Baillie, N. D. Burgess, K. Burkart, R. F. Noss, Y. P. Zhang, A. Baccini, T. Birch, N. Hahn, L. N. Joppa, E. Wikramanayake, A global deal for nature: Guiding principles, milestones, and targets. *Sci. Adv.* **5**, eaaw2869 (2019).

12. A. J. Hansen, P. Burns, J. Ervin, S. J. Goetz, M. Hansen, O. Venter, J. E. M. Watson, P. A. Jantz, A. L. S. Virnig, K. Barnett, R. Pillay, S. Atkinson, C. Supples, S. Rodríguez-Buritica, D. Armenteras, A policy-driven framework for conserving the best of Earth's remaining moist tropical forests. *Nat Ecol Evol.* **4**, 1377–1384 (2020).

13. W. Steffen, J. Rockström, K. Richardson, T. M. Lenton, C. Folke, D. Liverman, C. P. Summerhayes, A. D. Barnosky, S. E. Cornell, M. Crucifix, J. F. Donges, I. Fetzer, S. J. Lade, M. Scheffer, R. Winkelmann, H. J. Schellnhuber, Trajectories of the Earth system in the anthropocene. *Proc. Natl. Acad. Sci.* **115**, 8252–8259 (2018).

14. I. R. Leal, J. M. C. Da Silva, M. Tabarelli, T. E. Lacher, Changing the course of biodiversity conservation in the Caatinga of Northeastern Brazil. *Conserv. Biol.* **19**, 701–706 (2005).

15. S. R. Freitas, T. J. Hawbaker, J. P. Metzger, Effects of roads, topography, and land use on forest cover dynamics in the Brazilian Atlantic Forest. *For. Ecol. Manage.* **259**, 410–417 (2010).

16. Empresa de Planejamento e Logistica, *Relatório Executivo: Plano Nacional de Logística PNL - 2025* (Empresa de Planejamento e Logística S.A., Brasília, Distrito Federal, 2018); www.epl.gov.br/plano-nacional-de-logistica-pnl.

17. Brazil, *Lei n° 5.917, de 10 de Setembro de 1973* (1973); www.planalto.gov.br/ccivil_03/LEIS/L5917.htm#anexo.

18. R. K. de Oliveira, C. V. Andreoli, P. da Mata Cavalcante, in *Corporate Social Responsibility in Brazil: The Future is Now*, C. Stehr, N. Dziatzko, F. Struve, Eds. (CSR, Sustainability, Ethics & Governance, Springer International Publishing AG, 2019; https://doi.org/10.1007/978-3-319-90605-8_10), pp. 213–240.

19. B. B. N. Strassburg, A. E. Latawiec, L. G. Barioni, C. A. Nobre, V. P. da Silva, J. F. Valentim, M. Vianna, E. D. Assad, When enough should be enough: Improving the use of current agricultural lands could meet production demands and spare natural habitats in Brazil. *Glob. Environ. Chang.* **28**, 84–97 (2014).

20. Brazil, *Decreto n° 2.519, de 16 de março de 1998* (1998); www.planalto.gov.br/ccivil_03/decreto/D2519.htm.

21. Brazil, *Lei nº 12.187, de 29 de dezembro de 2009* (2009); www.planalto.gov.br/ccivil_03/_ato2007-2010/2009/lei/l12187.htm.

22. Brazil, *Decreto nº 9.073, de 5 de junho de 2017* (2017); www.planalto.gov.br/ccivil_03/_ato2015-2018/2017/decreto/D9073.htm.

23. IPEA, *Agenda 2030 ODS-Metas Nacionais dos Objetivos de Desenvolvimento Sustentável* (Instituto de Pesquisa Econômica Aplicada, 2018), p. 546.

24. R. Dave, C. Saint-Laurent, L. Murray, G. Antunes Daldegan, R. Brouwer, C. A. de Mattos Scaramuzza, L. Raes, S. Simonit, M. Catapan, G. García Contreras, A. Ndoli, C. Karangwa, N. Perera, S. Hingorani, T. Pearson, *Second Bonn Challenge Progress Report: Application of the Barometer in 2018* (IUCN, International Union for Conservation of Nature, 2019); https://portals.iucn.org/library/node/48446.

25. Brazil, *Intended Nationally Determined Contribution towards achieving the Objective of the United Nations Framework Convention on Climate Change* (2016); www4.unfccc.int/submissions/INDC/Published%20Documents/Brazil/1/BRAZIL%20iNDC%20english%20FINAL.pdf.

26. M. M. Vale, E. Berenguer, M. Argollo de Menezes, E. B. Viveiros de Castro, L. Pugliese de Siqueira, R. de C. Q. Portela, The COVID-19 pandemic as an opportunity to weaken environmental protection in Brazil. *Biol. Conserv.* **255**, 108994 (2021).

27. V. M. Azevedo-Santos, P. M. Fearnside, C. S. Oliveira, A. A. Padial, F. M. Pelicice, D. P. Lima, D. Simberloff, T. E. Lovejoy, A. L. B. Magalhães, M. L. Orsi, A. A. Agostinho, F. A.

Esteves, P. S. Pompeu, W. F. Laurance, M. Petrere, R. P. Mormul, J. R. S. Vitule, Removing the abyss between conservation science and policy decisions in Brazil. *Biodivers. Conserv.* **26**, 1745–1752 (2017).

28. R. Rajão, B. Soares-filho, F. Nunes, J. Börner, L. Machado, D. Assis, A. Oliveira, L. Pinto, V. Ribeiro, L. Rausch, H. Gibbs, The rotten apples of Brazil's agribusiness. *Science* **369**, 246–248 (2020).

29. Brazil, *Brazil's new First Nationally Determined Contribution towards Achieving the Objective of the United Nations Framework Convention on Climate Change* (2020); www4.unfccc.int/sites/NDCStaging/Pages/Party.aspx?party=BRA.

30. K. Yamamoto, M. J. Woods, Brazilian NDC reduces the country's climate ambition. *WWF* (2020); wwf.panda.org/wwf_news/?1173241/WWF-Brazil-NDC.

31. L. Ferrante, M. Gomes, P. M. Fearnside, Amazonian indigenous peoples are threatened by Brazil's Highway BR-319. *Land Use Policy* **94**, 104548 (2020).

32. P. Potapov, M. C. Hansen, L. Laestadius, S. Turubanova, A. Yaroshenko, C. Thies, W. Smith, I. Zhuravleva, A. Komarova, S. Minnemeyer, E. Esipova, The last frontiers of wilderness: Tracking loss of intact forest landscapes from 2000 to 2013. *Sci. Adv.* **3**, e1600821 (2017).

33. A. C. Malvestio, T. B. Fischer, M. Montaño, The consideration of environmental and social issues in transport policy, plan and programme making in Brazil: A systems analysis. *J. Clean. Prod.* **179**, 674–689 (2018).

34. S. G. Rabello Quadros, C. D. Nassi, An evaluation on the criteria to prioritize transportation infrastructure investments in Brazil. *Transp. Policy* **40**, 8–16 (2015).

35. S. Díaz, J. Settele, E. S. Brondízio, H. T. Ngo, J. Agard, A. Arneth, P. Balvanera, K. A. Brauman, S. H. M. Butchart, K. M. A. Chan, L. A. Garibaldi, K. Ichii, J. Liu, S. M. Subramanian, G. F. Midgley, P. Miloslavich, Z. Molnár, D. Obura, A. Pfaff, S. Polasky, A. Purvis, J. Razzaque, B. Reyers, R. R. Chowdhury, Y.-J. Shin, I. Visseren-Hamakers, K. J.

Willis, C. N. Zayas, Pervasive human-driven decline of life on Earth points to the need for transformative change. *Science* **366**, eaax3100 (2019).

36. C. Wyborn, M. C. Evans, Conservation needs to break free from global priority mapping. *Nat. Ecol. Evol.* **5**, 1322–1324 (2021).

37. G. Schmidt-Traub, National climate and biodiversity strategies are hamstrung by a lack of maps. *Nat. Ecol. Evol.* **5**, 1325–1327 (2021).

38. MMA, Cadastro Nacional de Unidades de Conservação (2018); www.mma.gov.br/areas-protegidas/cadastro-nacional-de-ucs/dados-georreferenciados.

39. FUNAI, Terras Indígenas/Terras Indígenas em Estudos 2018 (2018); www.funai.gov.br/index.php/shape.

40. INCRA, Quilombolas Brasil. *I3Geo Geodados* (2018); http://acervofundiario.incra.gov.br/geodownload/geodados.php.

41. MMA, *Portaria nº 463, de 18 de dezembro de 2018* (Brazil, 2018); www.in.gov.br/materia/-/asset_publisher/Kujrw0TZC2Mb/content/id/55881195/do1-2018-12-19-portaria-n-463-de-18-de-dezembro-de-2018-55880954.

42. Project MapBiomas, *Collection 3.0 of Brazilian Land Cover & Use Map Series* (2018); www.mapbiomas.org.

43. MMA, *National Biodiversity Strategy and Action Plan* (Ministério do Meio Ambiente, 2017), pp. 1–10.

44. R. Dave, C. Saint-Laurent, M. Moraes, S. Simonit, L. Raes, C. Karangwa, *Bonn Challenge Barometer of Progress: Spotlight Report 2017* (International Union for Conservation of Nature and Natural Resources, Gland, Switzerland, 2017), p. 36.

45. E. Rodríguez Mega, ‘Apocalyptic’ fires are ravaging the world’s largest tropical wetland. *Nature* **586**, 20–21 (2020).

46. I. Amigo, When will the Amazon hit a tipping point? *Nature* **578**, 505–507 (2020).

47. L. Ferrante, P. M. Fearnside, Brazil's new president and 'ruralists' threaten Amazonia's environment, traditional peoples and the global climate. *Envir. Conserv.* **46**, 261–263 (2019).

48. L. Ferrante, R. I. Barbosa, L. Duczmal, P. M. Fearnside, Brazil's planned exploitation of Amazonian indigenous lands for commercial agriculture increases risk of new pandemics. *Reg. Environ. Change* **21**, 81 (2021).

49. Brazil, *Lei nº 12.651, de 25 de Maio de 2012* (Brazil, 2012); www.planalto.gov.br/ccivil_03/_Ato2011-2014/2012/Lei/L12651.htm#art83.

50. J. Chiavari, C. Leme Lopes, J. Nardi de Araujo, *Onde estamos na implementação do código florestal? Radiografia do car e do pra nos estados brasileiros* (Climate Policy Initiative & INPUT Brasil, 2020); www.climatepolicyinitiative.org/publication/where-are-we-at-implementing-the-forest-code-an-x-ray-of-the-car-and-the-pra-in-brazilian-states/.

51. P. Amaral, T. Reis, R. del Giudice, *Guia prático para a análise do atendimento ao Código Florestal* (Observatório do Código Floresta, 2017); https://observatorioflorestal.org.br/en/guia-pratico-para-a-analise-do-atendimento-ao-codigo-florestal/.

52. M. E. Skidmore, F. Moffette, L. Rausch, M. Christie, J. Munger, H. K. Gibbs, Cattle ranchers and deforestation in the Brazilian Amazon: Production, location, and policies. *Glob. Environ. Chang.* **68**, 102280 (2021).

53. P. Gasparinetti, T. Vilela, *Implementando Mercados de Cotas de Reserva Ambiental (CRA) nos Estados Brasileiros: Desafios e Oportunidades para as Regulamentações Estaduais* (2018); www.conservation-strategy.org/sites/default/files/field-file/PORT_documento_de_discussao_CRA_CSF_Fev2018.pdf.

54. Á. Fernández-Llamazares, J. Helle, J. Eklund, A. Balmford, R. Mónica Moraes, V. Reyes-García, M. Cabeza, New law puts Bolivian biodiversity hotspot on road to deforestation. *Curr. Biol.* **28**, R15–R16 (2018).

55. R. E. Golden Kroner, S. Qin, C. N. Cook, R. Krithivasan, S. M. Pack, O. D. Bonilla, K. A. Cort-Kansinally, B. Coutinho, M. Feng, M. I. Martínez Garcia, Y. He, C. J. Kennedy, C. Lebreton, J. C. Ledezma, T. E. Lovejoy, D. A. Luther, Y. Parmanand, C. A. Ruíz-Agudelo, E. Yerena, V. Morón Zambrano, M. B. Mascia, The uncertain future of protected lands and waters. *Science* **364**, 881–886 (2019).

56. M. Rodrigues, Brazilian road proposal threatens famed biodiversity hotspot. *Nature* **596**, 473–474 (2021).

57. C. A. Nobre, G. Sampaio, L. S. Borma, J. C. Castilla-Rubio, J. S. Silva, M. Cardoso, Land-use and climate change risks in the Amazon and the need of a novel sustainable development paradigm. *Proc. Natl. Acad. Sci.* **113**, 10759–10768 (2016).

58. G. E. Overbeck, J. M. Hermann, B. O. Andrade, I. I. Boldrini, K. Kiehl, A. Kirmer, C. Koch, J. Kollmann, S. T. Meyer, S. C. Müller, C. Nabinger, G. E. Pilger, J. P. P. Trindade, E. Vélez-Martin, E. A. Walker, D. G. Zimmermann, V. D. Pillar, Restoration Ecology in Brazil—Time to step out of the forest. *Natureza a Conservacao* **11**, 92–95 (2013).

59. C. Grilo, M. R. Coimbra, R. C. Cerqueira, P. Barbosa, R. A. P. Dornas, L. O. Gonçalves, F. Z. Teixeira, I. P. Coelho, B. R. Schmidt, D. L. K. Pacheco, G. Schuck, I. B. Esperando, J. A. Anza, J. Beduschi, N. R. Oliveira, P. F. Pinheiro, A. Bager, H. Secco, M. Guerreiro, C. F. Carvalho, A. C. Veloso, A. E. I. Custódio, O. Marçal, G. Ciocheti, J. Assis, M. C. Ribeiro, B. S. S. Francisco, J. J. Cherem, T. C. Trigo, M. M. A. Jardim, I. C. Franceschi, C. Espinosa, F. P. Tirelli, V. J. Rocha, M. L. Sekiama, G. P. Barbosa, H. R. Rossi, T. C. Moreira, M. Cervini, C. A. Rosa, L. G. Silva, C. M. M. Ferreira, A. César, J. Casella, S. L. Mendes, J. Zina, D. F. O. Bastos, R. A. T. Souza, P. A. Hartmann, A. C. G. Deffaci, J. Mulinari, S. C. Luzzi, T. Rezzadori, C. Kolcenti, T. X. Reis, V. S. C. Fonseca, C. F. Giorgi, R. P. Migliorini, C. B. Kasper, C. Bueno, M. Sobanski, A. P. F. G. Pereira, F. A. G. Andrade, M. E. B. Fernandes, L. L. C. Corrêa, A. Nepomuceno, A. Banhos, W. Hannibal, R. Fonseca, L. A. Costa, E. P. Medici, A. Croce, K. Werther, J. P. Oliveira, J. M. Ribeiro, M. Santi, A. E. Kawanami, L. Perles, C. Couto, D. S. Figueiró, E. Eizirik, A. A. Correia, F. M. Corrêa, D. Queirolo, A. L. Quagliatto, B. H. Saranholi, P. M. Galetti, K. G. Rodriguez-Castro, V. S. Braz, F. G. R.

França, G. Buss, J. A. Rezini, M. B. Lion, C. C. Cheida, A. C. R. Lacerda, C. H. Freitas, F. Venâncio, C. H. Adania, A. F. Batisteli, C. G. Z. Hegel, J. A. Mantovani, F. H. G. Rodrigues, T. Bagatini, N. H. A. Curi, L. Emmert, R. H. Erdmann, R. R. G. F. Costa, A. Martinelli, C. V. F. Santos, A. Kindel, BRAZIL ROAD-KILL: A data set of wildlife terrestrial vertebrate road-kills. *Ecology* **99**, 2625 (2018).

60. N. D. Jackson, L. Fahrig, Relative effects of road mortality and decreased connectivity on population genetic diversity. *Biol. Conserv.* **144**, 3143–3148 (2011).

61. R. Beuchle, R. C. Grecchi, Y. E. Shimabukuro, R. Seliger, H. D. Eva, E. Sano, F. Achard, Land cover changes in the Brazilian Cerrado and Caatinga biomes from 1990 to 2010 based on a systematic remote sensing sampling approach. *Appl. Geography* **58**, 116–127 (2015).

62. E. E. Sano, A. A. Rodrigues, E. S. Martins, G. M. Bettiol, M. M. C. Bustamante, A. S. Bezerra, A. F. Couto, V. Vasconcelos, J. Schüler, E. L. Bolfe, Cerrado ecoregions: A spatial framework to assess and prioritize Brazilian savanna environmental diversity for conservation. *J. Environ. Manage.* **232**, 818–828 (2019).

63. M. J. Talty, K. Mott Lacroix, G. H. Aplet, R. T. Belote, Conservation value of national forest roadless areas. *Conservat. Sci. Prac.* **2**, e288 (2020).

64. J. D. Johnston, J. B. Kilbride, G. W. Meigs, C. J. Dunn, R. E. Kennedy, Does conserving roadless wildland increase wildfire activity in western U.S. national forests? *Environ. Res. Lett.* **16**, 084040 (2021).

65. M. Rosa, *Project MapBiomas, Mapa de Limite dos Biomas 1:1.000.000. Version 2.0* (2016); http://mapbiomas.org/pages/database/reference_maps.

66. IBGE, Base Cartográfica Continua do Brasil, 1:250.000 - BC250. *Versão 2017* (2017); ftp://geoftp.ibge.gov.br/cartas_e_mapas/bases_cartograficas_continuas/bc250/versao2017/shapefile/.

67. ESRI, *ArcGIS 10.4.1 for Desktop* (Environmental Systems Research Institute, Redlands, CA, USA, 2016); http://desktop.arcgis.com/en/.

68. R Core Team, *R: A Language and Environment for Statistical Computing* (R Foundation for Statistical Computing, Vienna, Austria, 2021); www.R-project.org/.

69. IBGE, *Brazilian Territorial Area* (2015); www.ibge.gov.br/english/geociencias/cartografia/default_territ_area.shtm.

70. Geofabrik, *OpenStreetMap, OpenStreetMap data for Brazil* (2018); http://download.geofabrik.de/south-america/brazil-latest-free.shp.zip.

71. M. L. Richardson, B. A. Wilson, D. A. S. Aiuto, J. E. Crosby, A. Alonso, F. Dallmeier, G. K. Golinski, A review of the impact of pipelines and power lines on biodiversity and strategies for mitigation. *Biodivers. Conserv.* **26**, 1801–1815 (2017).

72. CIA, Brazil. *The World Factbook* (2018); www.cia.gov/library/publications/the-world-factbook/geos/print_br.html.

73. IBGE, *Mapa de Biomas do Brasil* (2006); ftp://geoftp.ibge.gov.br/informacoes_ambientais/estudos_ambientais/biomas/.

74. MTPA, Banco de Informações de Transportes (2018); http://www.transportes.gov.br/bit.html.

75. ANTT, Malha Ferroviária Federal Georreferenciada. *Declaração de Rede* (2016); www.antt.gov.br/ferrovias/Declaracao_de_Rede.html.

76. A. Pfaff, J. Robalino, C. Sandoval, D. Herrera, Protected area types, strategies and impacts in Brazil's Amazon: Public protected area strategies do not yield a consistent ranking of protected area types by impact. *Philos. Trans. R. Soc. B Biol. Sci.* **370**, 20140273 (2015).

77. A. T. Tesfaw, A. Pfaff, R. E. Golden Kroner, S. Qin, R. Medeiros, M. B. Mascia, Land-use and land-cover change shape the sustainability and impacts of protected areas. *Proc. Natl. Acad. Sci.* **115**, 2084–2089 (2018).

78. E. N. Kere, J. Choumert, P. Combes Motel, J. L. Combes, O. Santoni, S. Schwartz, Addressing contextual and location biases in the assessment of protected areas effectiveness on deforestation in the Brazilian Amazônia. *Ecol. Econ.* **136**, 148–158 (2017).

79. Brazil, *Lei n° 9.985, de 18 de Julho de 2000* (2000); www.planalto.gov.br/ccivil_03/leis/L9985.htm.

80. T. Carranza, A. Balmford, V. Kapos, A. Manica, Protected area effectiveness in reducing conversion in a rapidly vanishing ecosystem: The Brazilian Cerrado. *Conserv. Lett.* **7**, 216–223 (2014).

81. J. Hargrave, K. Kis-Katos, Economic causes of deforestation in the Brazilian Amazon: A panel data analysis for the 2000s. *Environ. Resource Econ.* **54**, 471–494 (2013).

82. E. M. Nogueira, A. M. Yanai, S. S. de Vasconcelos, P. M. L. de Alencastro Graça, P. M. Fearnside, Carbon stocks and losses to deforestation in protected areas in Brazilian Amazonia. *Reg. Environ. Change* **18**, 261–270 (2017).

83. C. Nolte, A. Agrawal, K. M. Silvius, B. S. Soares-Filho, Governance regime and location influence avoided deforestation success of protected areas in the Brazilian Amazon. *Proc. Natl. Acad. Sci.* **110**, 4956–4961 (2013).

84. B. Soares-Filho, P. Moutinho, D. Nepstad, A. Anderson, H. Rodrigues, R. Garcia, L. Dietzsch, F. Merry, M. Bowman, L. Hissa, R. Silvestrini, C. Maretti, Role of Brazilian Amazon protected areas in climate change mitigation. *Proc. Natl. Acad. Sci.* **107**, 10821–10826 (2010).

85. W. Walker, A. Baccini, S. Schwartzman, S. Ríos, M. A. Oliveira-Miranda, C. Augusto, M. R. Ruiz, C. S. Arrasco, B. Ricardo, R. Smith, C. Meyer, J. C. Jintiach, E. V. Campos, Forest carbon in Amazonia: The unrecognized contribution of indigenous territories and protected natural areas. *Carbon Manage.* **5**, 479–485 (2014).

86. E. M. Nogueira, A. M. Yanai, S. S. de Vasconcelos, P. M. L. de Alencastro Graça, P. M. Fearnside, Brazil's Amazonian protected areas as a bulwark against regional climate change. *Regional Environ. Change.* **18**, 573–579 (2018).

87. I. Tritsch, F. M. Le Tourneau, Population densities and deforestation in the Brazilian Amazon: New insights on the current human settlement patterns. *Appl. Geogr.* **76**, 163–172 (2016).

88. Brazil, *Decreto n° 1.775, de 8 de janeiro de 1996* (1996); www.planalto.gov.br/ccivil_03/decreto/D1775.htm.

89. Brazil, *Decreto nº 4.887, de 20 de novembro de 2003* (2003); www.planalto.gov.br/ccivil_03/decreto/2003/d4887.htm.

90. R. D. Costa, in *Insecure Land Rights in Brazil: Consequences for Rural Areas and Challenges for Improvement* (Climate Policy Initiative, 2016), pp. 1–17.

91. R. D. Costa, J. Chiavari, C. Leme Lopes, J. Campos, Mariana; Roche, *Evolution of Land Rights in Rural Brazil: Frameworks for Understanding Pathways for Improvement* (Climate Policy Initiative, 2017); https://climatepolicyinitiative.org/publication/evolution-of-land-rights-in-rural-brazil/.

92. FUNAI, Demarcação de Terras Indígenas (2018), pp. 12–14.

93. INCRA, *Instrução Normativa n° 56, de 7 de outubro de 2009* (Instituto Nacional de Colonização e Reforma Agrária, 2009).

94. INCRA, *Instrução Normativa n° 57, de 20 de outubro de 2009* (Instituto Nacional de Colonização e Reforma Agrária, 2009).

95. A. BenYishay, S. Heuser, D. Runfola, R. Trichler, Indigenous land rights and deforestation: Evidence from the Brazilian Amazon. *J. Environ. Eco. Manage.* **86**, 29–47 (2017).

96. Brazil, *Decreto n° 9.082, de 26 de Junho de 2017* (2017); www.planalto.gov.br/ccivil_03/_Ato2015-2018/2017/Decreto/D9082.htm%20n.

97. FBMC, *Proposta Inicial de Implementação da Contribuição Nacionalmente Determinada do Brasil (NDC)* (Fórum Brasileiro de Mudança do Clima, 2017); https://drive.google.com/file/d/1puFdkXpY3Ms8yyMPB7z2mIen7dDcFiha/view.

98. MMA, 2ª Atualização das Áreas Prioritárias para Conservação da Biodiversidade 2016/2018 (2018); http://areasprioritarias.mma.gov.br/2-atualizacao-das-areas-prioritarias.

99. OpenStreetMap Wiki, Key:highway (2019); https://wiki.openstreetmap.org/w/index.php?title=Key:highway&oldid=1787212.
